# *ortho*-Selective C–H addition of *N*,*N*-dimethyl anilines to alkenes by a yttrium catalyst†
†Electronic supplementary information (ESI) available. See DOI: 10.1039/c6sc00833j


**DOI:** 10.1039/c6sc00833j

**Published:** 2016-04-26

**Authors:** Guoyong Song, Gen Luo, Juzo Oyamada, Yi Luo, Zhaomin Hou

**Affiliations:** a Organometallic Chemistry Laboratory and RIKEN Center for Sustainable Resource Science , RIKEN , Wako , Saitama 351-0198 , Japan . Email: houz@riken.jp; b Beijing Key Laboratory of Lignocellulosic Chemistry , Beijing Forestry University , Beijing 100083 , China; c State Key Laboratory of Fine Chemicals , School of Pharmaceutical Science and Technology , Dalian University of Technology , Dalian 116024 , China . Email: luoyi@dlut.edu.cn

## Abstract

The efficient and selective *ortho*-alkylation of *N*,*N*-dimethyl anilines *via* C–H addition to alkenes was achieved for the first time using a cationic half-sandwich yttrium catalyst. This protocol constitutes a straightforward and atom-economical route for the synthesis of a new family of tertiary aniline derivatives with branched alkyl substituents, which are otherwise difficult to obtain. DFT calculation studies suggest that the interaction between the yttrium atom and the NMe_2_ group plays an important role and the intramolecular C–H activation through a σ-bond metathesis pathway is the rate-determining step, which is consistent with the experimental KIE observations.

## Abstract 2

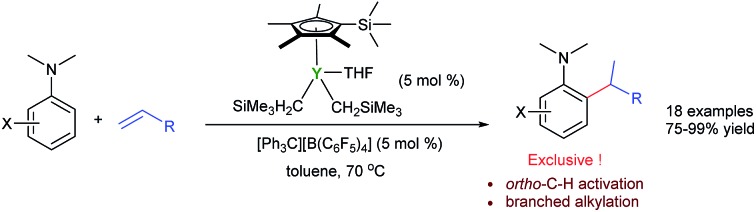
The efficient and selective *ortho*-alkylation of *N*,*N*-dimethyl anilines *via* C–H addition to alkenes has been achieved for the first time using a cationic half-sandwich yttrium catalyst.

## Introduction

Tertiary aniline is among the most important aromatic structural motifs in pharmaceuticals, fluorescent dyes, natural products, and organic functional materials.^1^ Therefore, the development of efficient, atom-economical processes for the synthesis of aniline-containing compounds through the direct C–H functionalization of aniline has received intensive attention.^2^–^7^ Among the possible approaches, catalytic C–H addition to alkenes is the most straightforward and atom-economical method for the preparation of alkylated aniline derivatives. However, the direct C–H alkylation of tertiary anilines has met with limited success to date, mainly because of the low activity of a dialkylamino group to serve as a directing group (DG) for transition-metal catalysed C–H activation and the easy β-H elimination of transition metal alkyl species.

**Scheme 1:**
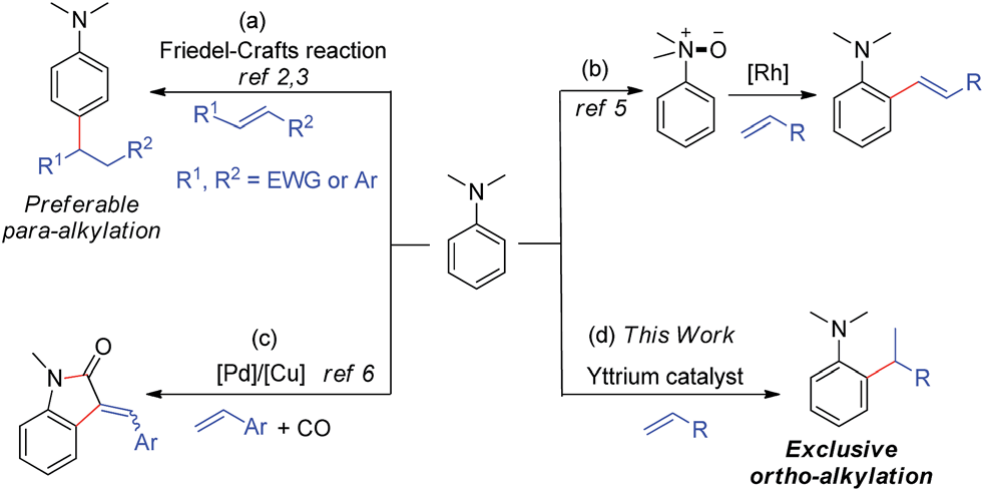
Catalytic C–H functionalization of *N*,*N*-dimethylaniline.

A few Friedel–Crafts reactions of dialkylanilines with activated electrophilic alkenes were reported.^2^,^3^ These reactions all likely proceeded through classic EAS (Electrophilic Aromatic Substitution) mechanisms, which favour *para*-selectivity for dialkylanilines and required either aryl substituents^2^ or strong electron-withdrawing groups,^3^ such as CHO, CO, and NO_2_, at the C=C double bond (Scheme 1a).

It is well known that the use of a directing group (DG) could lead to the *ortho*-selective C–H alkylation of arenes with alkenes in the presence of transition metal catalysts through cyclometalation.^8^ However, it is difficult for dialkylamino groups, such as NMe_2_ bonded directly to an aromatic ring to act as a DG for the activation of an *ortho* C–H bond by a late transition metal catalyst, because the reaction would require the formation of an unfavourable four-membered, cyclometallated intermediate.^4^–^7^ Shi and co-workers used a dimethylaminomethylene moiety (CH_2_NMe_2_) as a DG for the *ortho* C–H alkenylation of *N*,*N*-dimethylbenzylamines in the presence of a palladium catalyst through the formation of a five-membered palladacycle intermediate.^4^ You and co-workers used N(O)Me_2_ as an oxidizing DG for the rhodium-catalysed *ortho* C–H alkenylation of tertiary anilines (Scheme 1b).^5^ Lei and co-workers reported the palladium/copper-catalysed *ortho* C–H alkenylation/*N*-dealkylative carbonylation of *N*,*N*-dialkyl anilines with styrenes and CO, with partial loss of the tertiary amino moiety (Scheme 1c).^6^ To the best of our knowledge, the catalytic *ortho*-selective C–H functionalization (either alkylation or alkenylation) of a tertiary aniline has not been reported previously.^7^ Therefore, the search for new catalyst systems to achieve a more selective and efficient C–H functionalization of tertiary anilines is of great interest and importance.

Cationic half-sandwich rare earth alkyl complexes have recently emerged as a new class of highly efficient olefin polymerization catalysts.^9^ These catalysts can also efficiently catalyse the regio- and stereospecific C–H addition of some aromatic compounds, such as pyridines and anisoles, to alkenes with substrate scope and selectivity different from those of late transition metal catalysts.9a,^10^ In many cases, a four-membered heteroatom-containing metallacycle could work well for *ortho*-selective aromatic C–H activation and functionalization,10b,c thanks to the strong Lewis acidity or heteroatom (such as O and N) affinity of rare-earth metal ions. These results encouraged us to examine whether rare earth catalysts could work for the *ortho*-selective C–H alkylation of tertiary anilines with alkenes through the assistance of an interaction between the amino group and the rare earth metal ion.

Herein, we report the highly efficient, *ortho*-selective C–H addition of a wide range of *N*,*N*-dimethyl anilines to alkenes catalysed by a cationic half-sandwich yttrium alkyl complex. This transformation represents the first example of *ortho*-specific C–H alkylation of *N*,*N*-dialkyl anilines with alkenes, efficiently affording a new family of alkylated tertiary aniline derivatives, which are otherwise difficult to prepare. DFT studies were also performed to elucidate the reaction mechanism.

## Results and discussion

### Optimization studies

**Chart 1:**
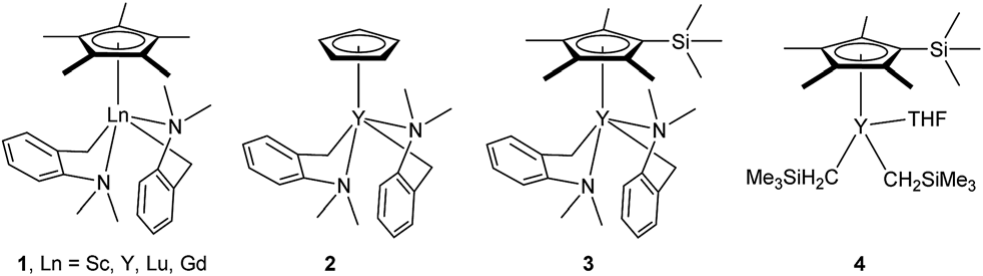
Selected examples of half-sandwich rare-earth dialkyl complexes.

At first, we examined the reaction of *N*,*N*-dimethylaniline (**5a**) with 1-octene (**6a**) using a series of half-sandwich rare-earth dialkyl complexes (Chart 1)^11^ in combination with an equivalent of [Ph_3_C][B(C_6_F_5_)_4_]. Significant influences of the ligands and the metal ions of the catalysts were observed (Table 1), suggesting that an appropriate metal/ligand combination is highly important for this reaction. Among the catalysts examined, the C_5_Me_4_SiMe_3_-ligated yttrium bis(trimethylsilylmethyl) complex **4** showed the highest activity, affording the corresponding branched, *ortho*-C–H alkylation product **7a** in 95% yield at 70 °C in 16 h with a 5 mol% catalyst loading (Table 1, entry 6).^12^ No *para*- or *meta*-alkylation product was observed, in contrast to what was observed in the Friedel–Crafts alkylation of tertiary anilines.^2^,^3^ The present *ortho*-selective alkylation suggests that the interaction between the NMe_2_ group and the metal centre should play an important role. It is also worth noting that only the branched alkylation product was observed in this reaction, standing in sharp contrast with late transition metal-catalysed C–H alkylation of aromatic compounds with 1-alkenes, which always gave the linear isomer as the predominant product.^8^ The analogous Sc, Gd or Lu complexes were not effective for this reaction under similar conditions (Table 1, entry 1). The neutral complex **4** or [Ph_3_C][B(C_6_F_5_)_4_] alone did not show any catalytic activity, suggesting that a cationic half-sandwich yttrium alkyl species is essential in the present transformation.

**Table 1:** *ortho* C–H alkylation of *N*,*N*-dimethylaniline with 1-octene by half-sandwich rare earth catalysts^*a*^

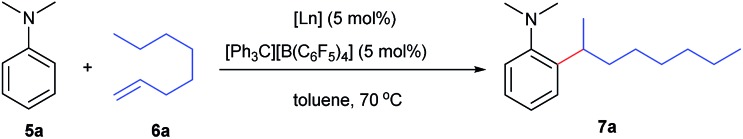				
Entry	Ln (cat.)	**6a** (equiv.)	Time (h)	Yield^*b*^ (%)
1	**1**-Sc, Lu or Gd	20	24	0
2	**1**-Y	20	24	76
3	**1**-Y	5	24	47
4	**2**	20	24	41
5	**3**	20	24	21
6	**4**	5	16	95 (93^*c*^)

^*a*^Reaction conditions: **5a** (0.4 mmol), **6a** (5 or 20 equiv.), [Ln] (5 mol%), [Ph_3_C][B(C_6_F_5_)_4_] (5 mol%), toluene (1.5 mL).

^*b*^NMR yield.

^*c*^Isolated yield.

### Substrate scope

The **4**/[Ph_3_C][B(C_6_F_5_)_4_] combination was then chosen as a catalyst to examine the reaction of *N*,*N*-dimethylaniline with other alkenes. 1-Hexene (**6b**), allylcyclohexane (**6c**) and 4-methyl-1-pentene (**6d**) could also be used as efficient alkylation agents, affording exclusively the corresponding branched *ortho*-C–H alkylation products in good yields (Table 2, entries 1–3). Similarly, the reaction of norbornene (**6e**) with *N*,*N*-dimethylaniline yielded quantitatively the *ortho*-norbornylation product **7e** (Table 2, entry 4). This reaction is in sharp contrast with the gold-catalysed Friedel–Crafts alkylation of *N*,*N*-dimethylaniline with norbornene, which afforded a mixture of *ortho*- and *para*-norbornylation products.^2^ Internal alkenes, such as 2-octene; or 1,1-disubstituted alkenes, such as α-methylstyrene; are not applicable under the same conditions, probably because of steric hindrance. No C–H alkylation was observed when an enone was used. These results suggest that the reaction mechanism of the present yttrium-catalysed alkylation should be distinctly different from those of the Lewis-acid-catalysed Friedel–Crafts reactions. The reactions of styrene and 1,3-cyclohexadiene with *N*,*N*-dimethylaniline gave oligomer products under similar conditions, probably because of their relatively high activity towards polymerization.^9^,^13^,^14^

**Table 2:** Yttrium-catalysed *ortho*-C–H alkylation of tertiary anilines with alkenes^*a*^

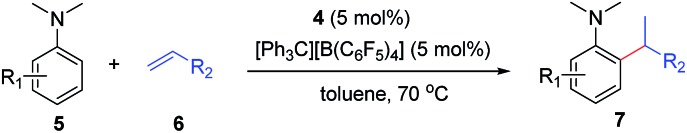											
Entry	**5**	**6**	Time	Product	Yield (%)	Entry	**5**	**6**	Time	Product	Yield (%)
1	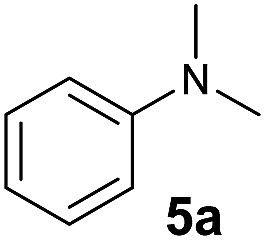	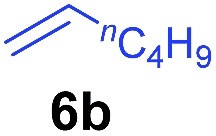	18 h	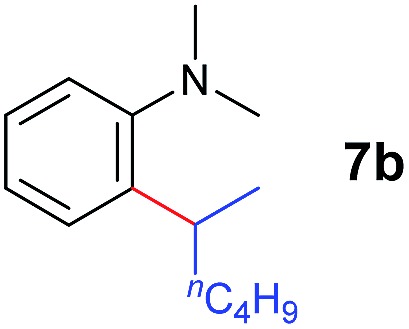	94%	10	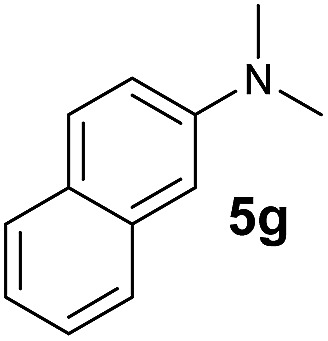	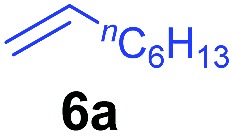	24 h	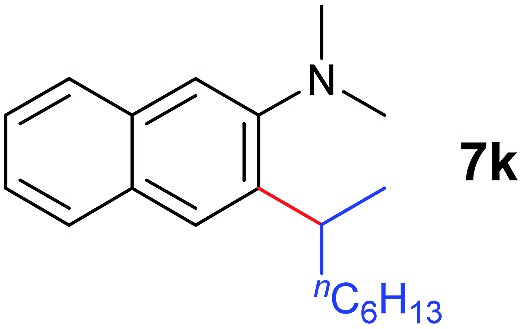	86%
2^*b*^	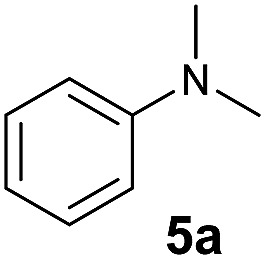	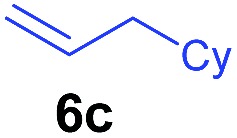	48 h	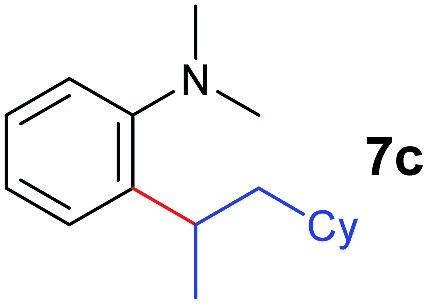	86%	11^*d*^	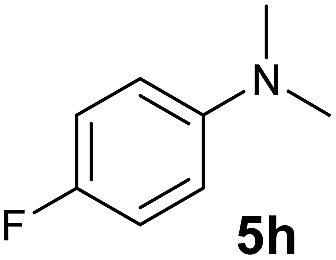	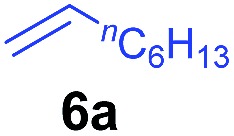	48 h	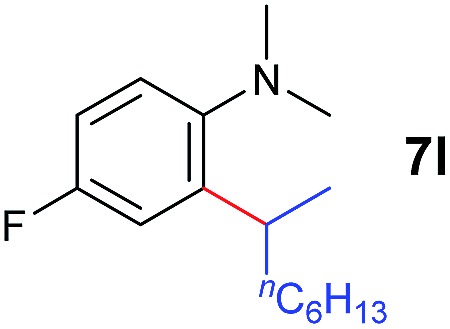	82%
3	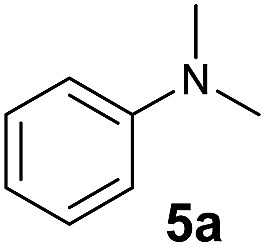	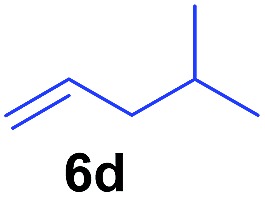	72 h	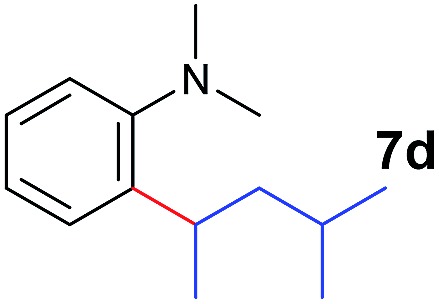	75%	12^*d*^	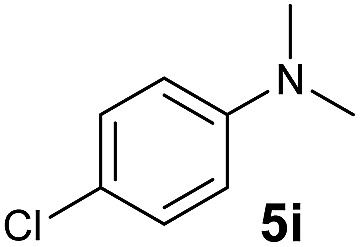	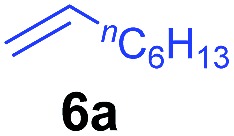	48 h	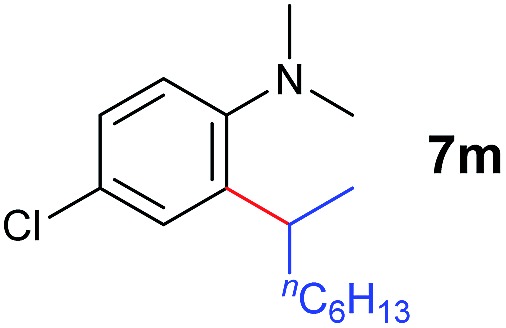	75%
4^*c*^	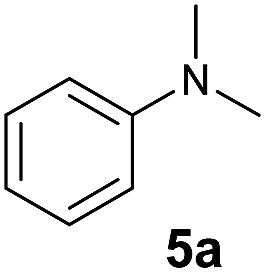	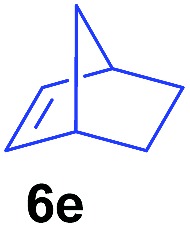	3 h	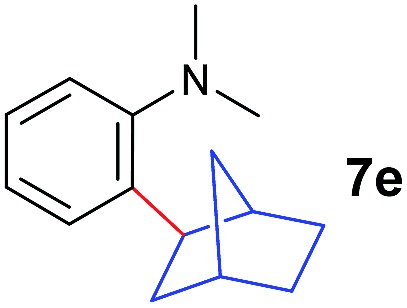	99%	13^*d*^	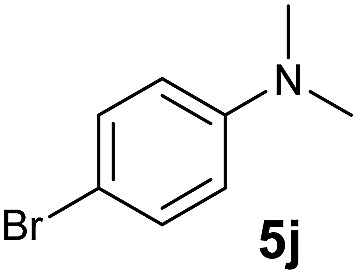	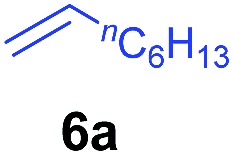	36 h	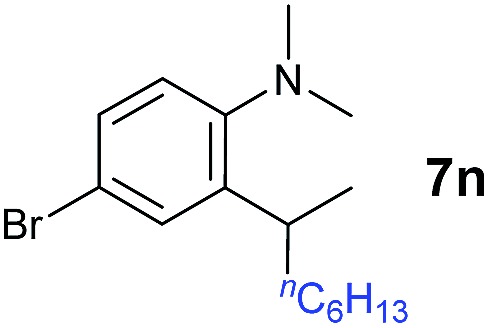	85%
5	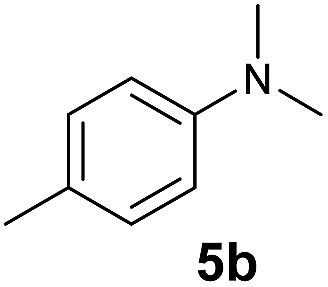	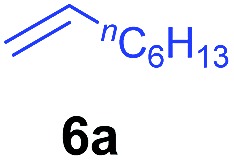	16 h	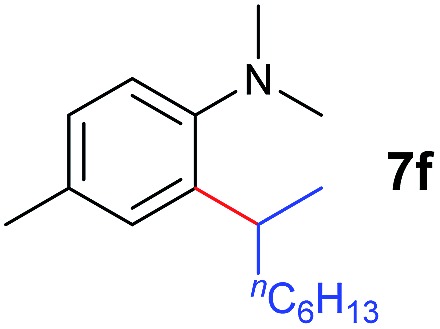	92%	14^*d*^	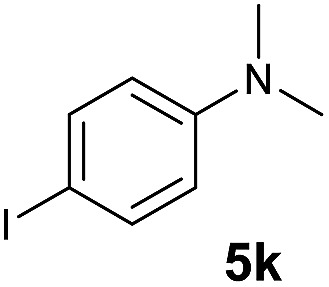	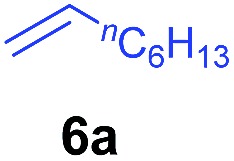	24 h	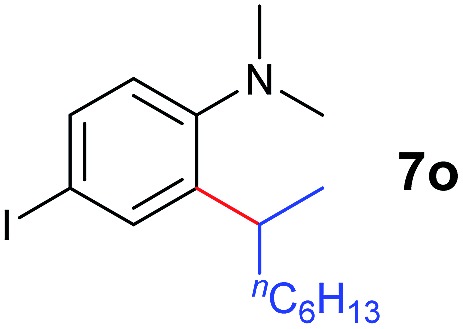	83%
6	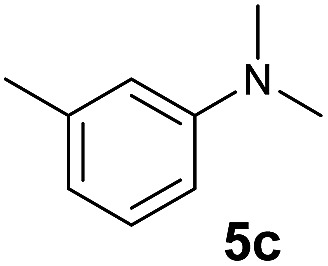	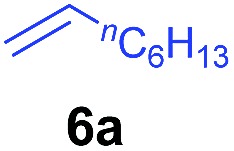	12 h	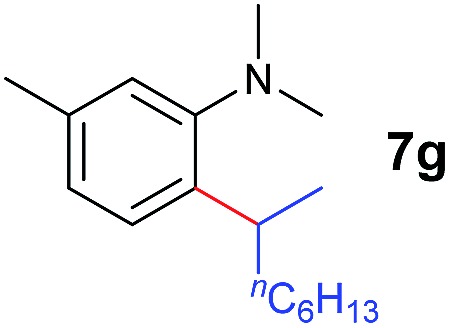	99%	15	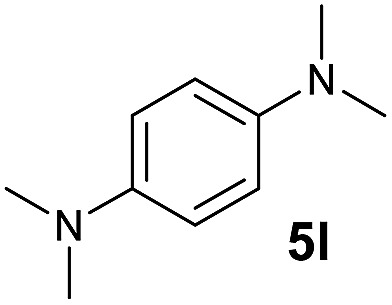	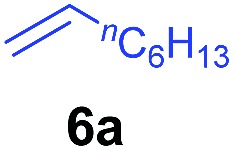	16 h	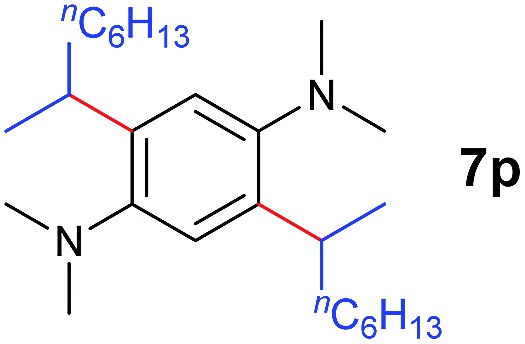	99%
7	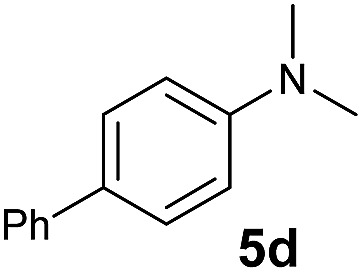	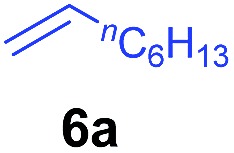	18 h	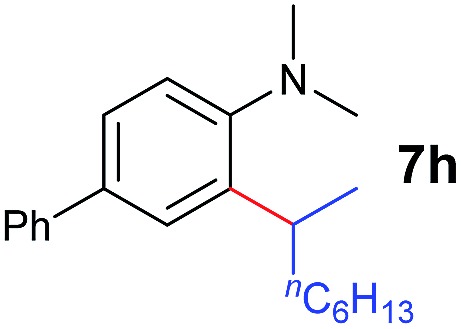	93%	16	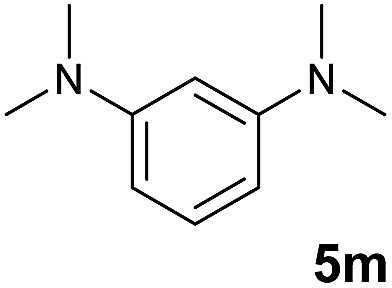	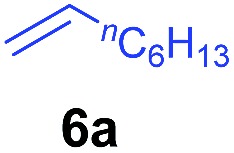	24 h	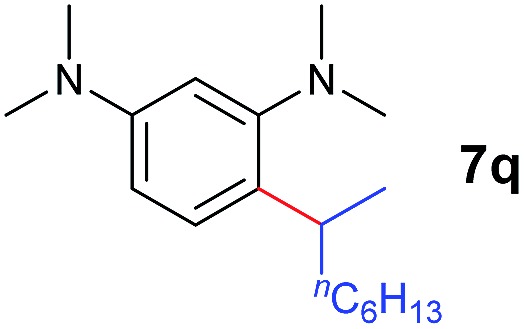	85%
8	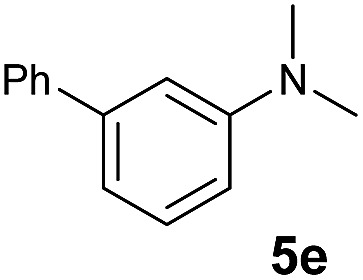	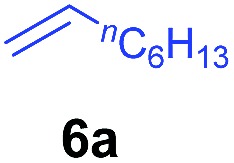	10 h	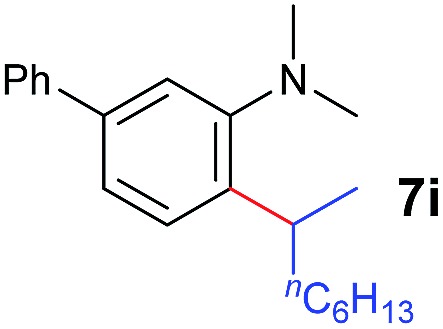	94%	17	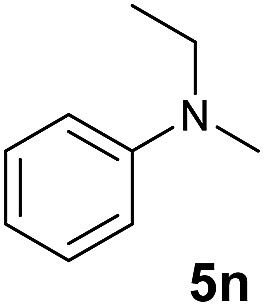	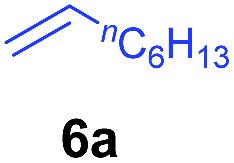	24 h	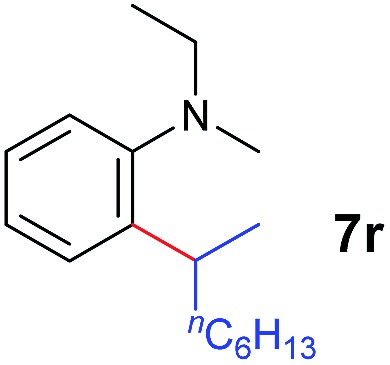	94%
9	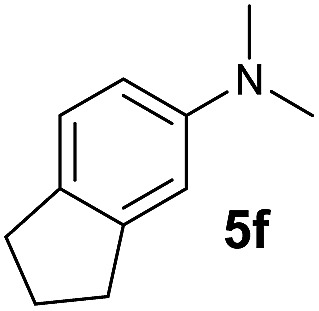	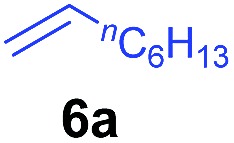	48 h	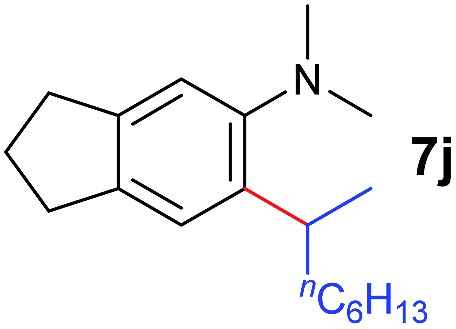	82%						

^*a*^Reaction conditions: **5** (0.4 mmol), **6** (2.0 mmol), catalyst **4** (5 mol%), [Ph_3_C][B(C_6_F_5_)_4_] (5 mol%), toluene (1.5 mL), isolated yields.

^*b*^Cy = cyclohexyl.

^*c*^Norbornene **6f**: 0.8 mmol.

^*d*^Catalyst **4** (8 mol%), [Ph_3_C][B(C_6_F_5_)_4_] (8 mol%).



1

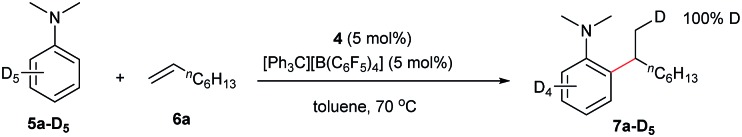



2

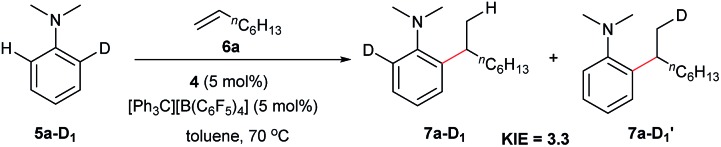



3

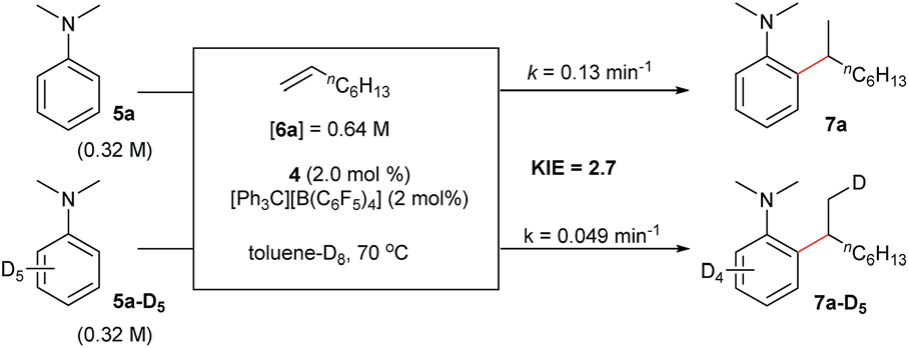




A broad range of *N*,*N*-dimethylaniline derivatives containing methyl, aryl and ring-fused substituents could be alkylated efficiently at the *ortho* position with 1-octene by the present yttrium catalyst, affording exclusively the corresponding branched alkylation products (Table 2, entries 5–10). Halogen (F, Cl, Br, and I)-substituted dimethylanilines (such as **5h–5k**) are compatible with the catalyst, yielding selectively the corresponding C–H alkylation products (Table, entries 11–14). No dehalogenation was observed. In the case of *N*,*N*,*N*′,*N*′-tetramethyl-*p*-phenylenediamine (**5l**), the alkylation reaction at an *ortho* position of each NMe_2_ group took place, selectively affording the branched *para*-dialkylation product **7p** in 97% yield (Table 2, entry 15). When *N*,*N*,*N*′,*N*′-tetramethyl-*m*-phenylenediamine (**5m**) was used as a substrate, the reaction took place selectively at a less hindered *ortho* C–H bond, yielding the mono-alkylation product **7q** exclusively (Table 2, entry 16). The formation of a dialkylation product was not observed.^15^ In addition to *N*,*N*-dimethylaniline, *N*-ethyl-*N*-methylaniline could also be selectively alkylated at the *ortho* C–H position by 1-octene, affording **7r** in 90% yield in a branched fashion (Table 2, entry 17). The alkylation reaction of *N*,*N*-dimethyl-*o*-toluidine, *N*,*N*-diethylaniline or 1-phenylpiperidine with 1-octene did not take place under the same conditions.^16^

### Deuterated experiments

The reaction of *N*,*N*-dimethylaniline-d_5_ (**5a-D_5_**) with 1-octene catalysed by **4**/[Ph_3_C][B(C_6_F_5_)_4_] afforded the C–D addition product **7a-D_5_**, in which a deuterium atom was incorporated selectively to the methyl group (eqn (1)). The reaction of **5a-D_1_** with 1-octene yielded a mixture of C–H and C–D alkylation products with a KIE (kinetic isotope effect) value of 3.3 (eqn (2)). The measurements of the initial rates of the two parallel reactions of **5a** and **5a-D_5_** with 1-octene gave a KIE value of 2.7 (eqn (3)). These results suggest that C–H activation may be involved in the rate-determining step of this transformation.^17^

### Computational (DFT) studies

**Scheme 2:**
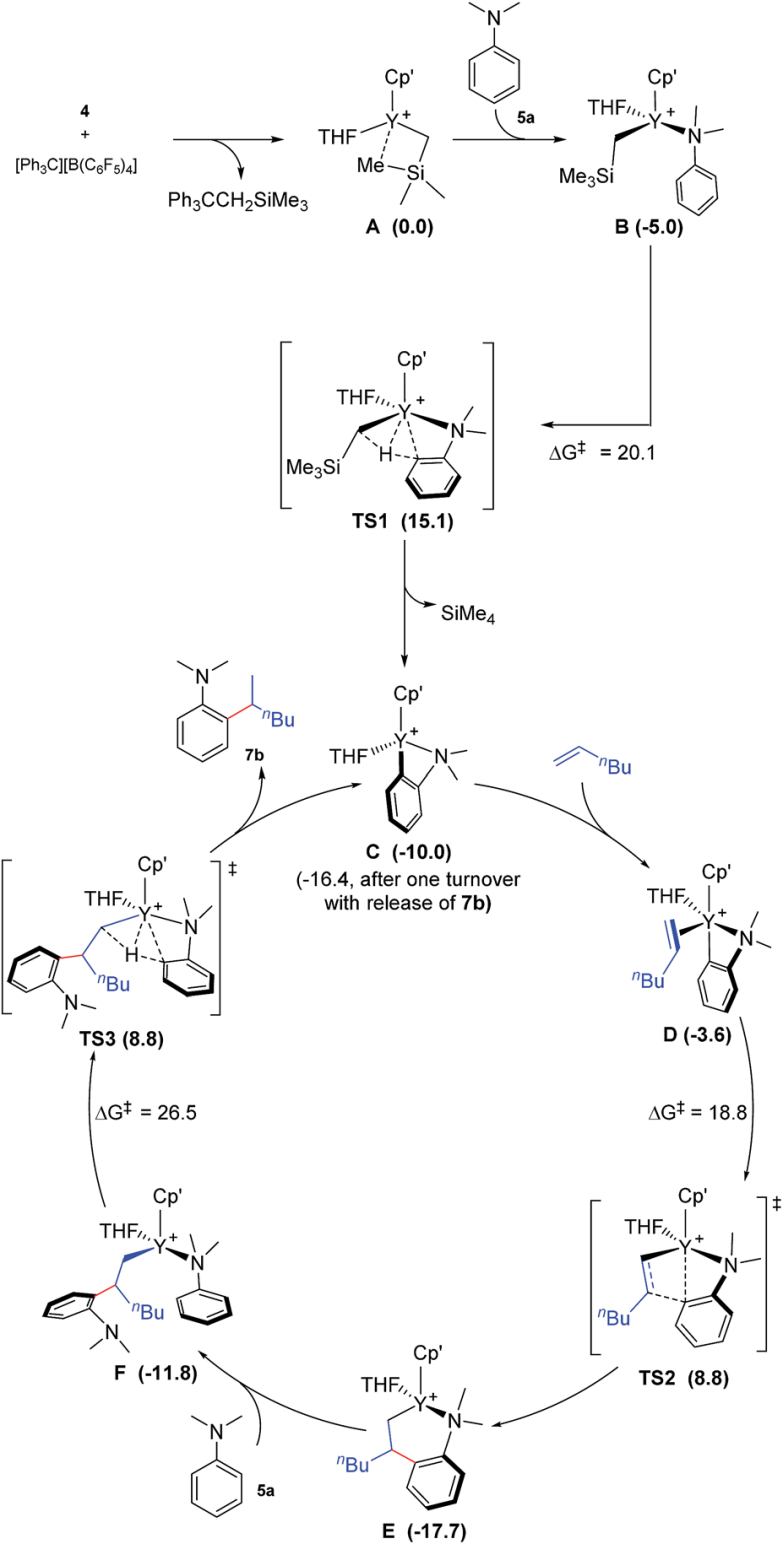
Possible catalytic cycle with the calculated free energy in parenthesis (kcal mol^–1^). Cp′ = C_5_Me_4_SiMe_3_. The energy values are relative to **A**.

**Fig. 1:**
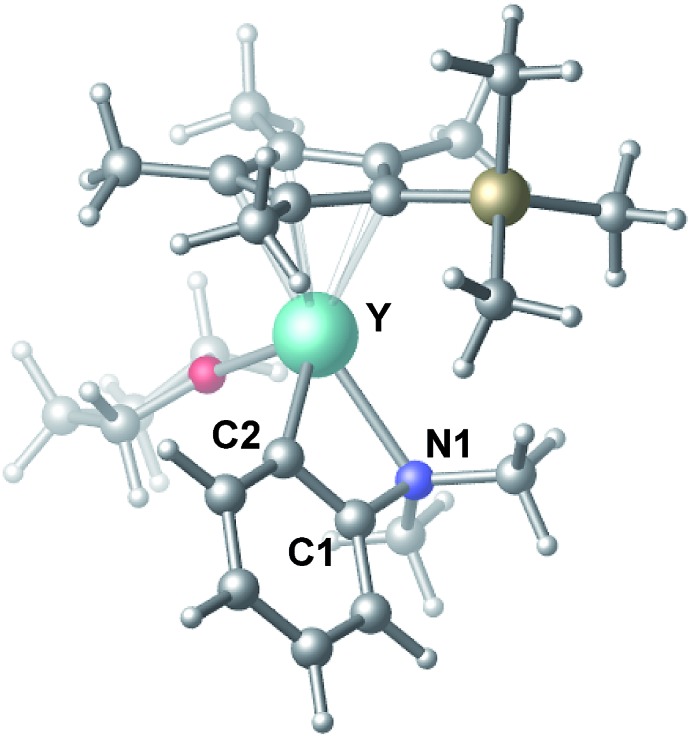
Optimized structure of **C**. Selected distances (Å) and angles (°): Y–N1 2.490; Y–C2 2.325; N1–Y–C2 61.18.

**Fig. 2:**
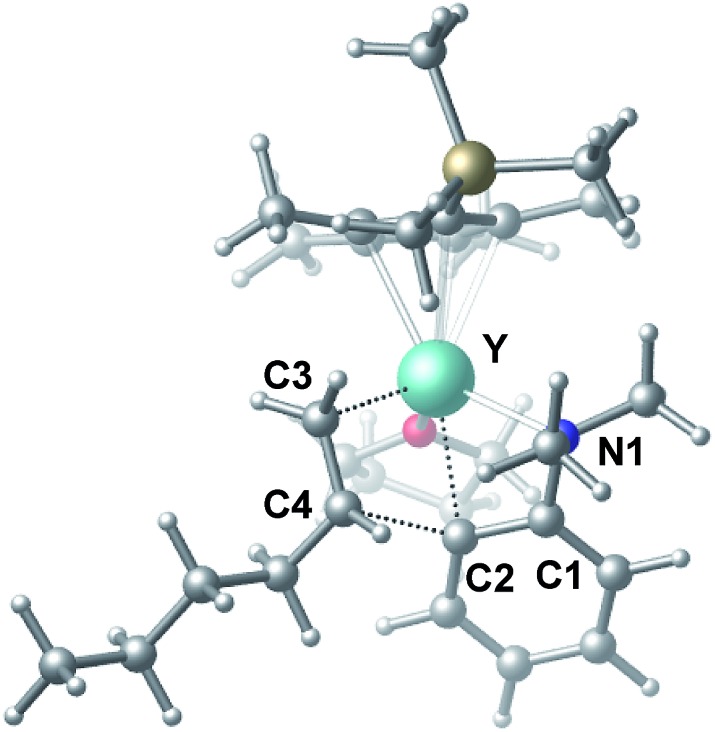
Optimized structure of the transition state **TS2**. Selected distances (Å) and angles (°): Y–N1 2.528; Y–C2 2.502; Y–C3 2.416; Y–C4 2.874; C4–C2 2.224; N1–Y–C2 58.18; C2–Y–C3 77.60.

**Fig. 3:**
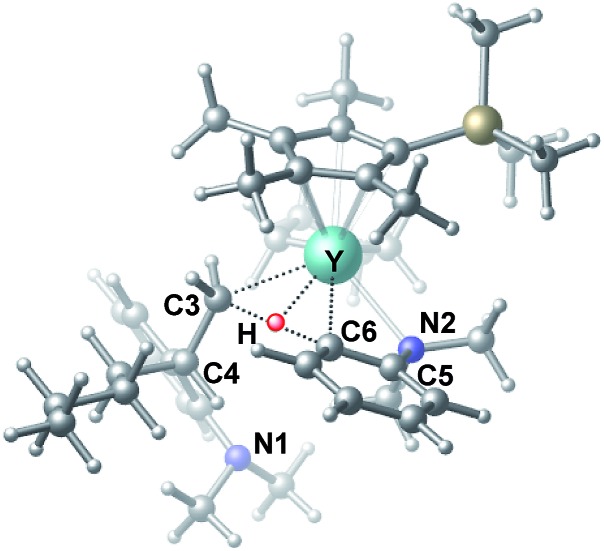
Optimized structure of the transition state **TS3**. Selected distances (Å) and angles (°): Y–N2 2.542; Y–C6 2.478; Y–C3 2.519; Y–H 2.064; C6–H 1.420; N2–Y–C6 57.73; C6–Y–C3 70.95.

To gain more insight into the mechanism of this transformation, we performed DFT calculations on the reaction of *N*,*N*-dimethylaniline with 1-hexene (Scheme 2). The Gibbs free energy at 343.15 K in toluene (solvent) is shown in Scheme 1.^18^ The coordination of *N*,*N*-dimethylaniline to a cationic yttrium alkyl species **A**^19^ generated from the reaction of **4** with [Ph_3_C][B(C_6_F_5_)_4_] gives **B**, which then undergoes proton transfer *via* a four-centre transition state **TS1** to afford an *o*-dimethylaminophenyl yttrium species **C** with the release of SiMe_4_.^20^ The dimethylaniline unit in **C** is bonded to the Y atom in a chelating fashion with both the NMe_2_ group and the *ortho* carbon atom (Fig. 1).^21^ The energy barrier for this process is 20.1 kcal mol^–1^. The coordination of the C=C double bond of 1-hexene to the Y atom in **C** can take place to form **D**, in which the ^*n*^Bu group in 1-hexene is oriented away from the C_5_Me_4_SiMe_3_ ligand to avoid steric repulsion.^22^ The 1,2-insertion of 1-hexene into yttrium–phenyl bond would be sterically favoured, thus giving a six-membered metallacycle complex **E** through a four-centre transition state **TS2** with an energy barrier of 18.8 kcal mol^–1^. Like the agostic interaction existing in the insertion transition state of olefin polymerization,^9^ the coordination of NMe_2_ to Y plays an important role in stabilizing the structure of **TS2** (Fig. 2). The coordination of *N*,*N*-dimethylaniline (**5a**) to **E** affords **F**, which then undergoes intramolecular C–H activation through **TS3** to give the species **C**, with the release of the final branched alkylation product **7b**. Similar to **TS1**, **TS3** also has a four-centre structure involving proton transfer, which is indicative of a one-step σ-bond metathesis C–H activation process (Fig. 3). This proton transfer process has the highest energy barrier (Δ*G*^‡^ = 26.5 kcal mol^–1^) in this catalytic cycle, which is in agreement with the experimental observations of KIE. The net energy of this catalytic process is exothermic by 16.4 kcal mol^–1^ after one turnover. It should also be noted that the THF ligand is always ligated to the yttrium centre in the whole reaction process (see ESI†).

## Conclusions

By using an yttrium catalyst, we achieved for the first time the *ortho*-selective C–H alkylation of tertiary anilines with 1-alkenes. This protocol features exclusive *ortho*-regioselectivity, excellent atom economy, broad substrate scope, and certain functional group tolerance, efficiently affording a new family of tertiary aniline derivatives with branched alkyl substituents. DFT studies showed that the interaction between the amino group in the aniline substrate and the yttrium atom in the catalyst plays an important role in the *ortho* selective C–H activation of the aniline moiety. The intramolecular σ-bond metathesis reaction between an yttrium alkyl species and an *ortho* C–H bond of the aniline moiety is the rate-determining step, which is in agreement with the experimental KIE observations. This study demonstrates that rare earth metal catalysts can promote C–H functionalization, which is difficult to achieve with late transition metal catalysts, due to the unique heteroatom affinity of the rare earth metal ions.

## Supplementary Material

Supplementary information

## References

[cit1] (a) Waser M., in Progress in the Chemistry of Organic Natural Products, ed. A. D. Kinghorn, H. Falk and J. Kobayashi, Springer, New York, 2012, vol. 96.

[cit2] Hu X., Martin D., Melaimi M., Bertrand G. (2014). J. Am. Chem. Soc..

[cit3] Paras N. A., MacMillan D. W. C. (2002). J. Am. Chem. Soc..

[cit4] Cai G., Fu Y., Li Y., Wan X., Shi Z. (2007). J. Am. Chem. Soc..

[cit5] Huang X., Huang J., Du C., Zhang X., Song F., You J. (2013). Angew. Chem., Int. Ed..

[cit6] Shi R., Lu L., Zhang H., Chen B., Sha Y., Liu C., Lei A. (2013). Angew. Chem., Int. Ed..

[cit7] Prades A., Corberán R., Poyatos M., Peris E. (2009). Chem.–Eur. J..

[cit8] Murai S., Kakiuchi F., Sekine S., Tanaka Y., Kamatani A., Sonoda M., Chatani N. (1993). Nature.

[cit9] Nishiura M., Guo F., Hou Z. (2015). Acc. Chem. Res..

[cit10] Guan B.-T., Hou Z. (2011). J. Am. Chem. Soc..

[cit11] Shima T., Nishiura M., Hou Z. (2011). Organometallics.

[cit12] The deprotonation of *N*,*N*-dimethylaniline by the aminobenzyl complexes **1–3**, which possess a relatively stable five-membered metallacycle, would give a less stable, four-membered metallacycle species like C shown in Scheme 2. This might account for the lower activity of **1–3** than that of the analogous trimethylsilylmethyl complex **4**

[cit13] Luo Y., Baldamus J., Hou Z. (2004). J. Am. Chem. Soc..

[cit14] The DFT calculations suggest that the continuous insertion of styrene into the newly formed yttrium–carbon bond is more favourable than the deportation of *N*,*N*-dimethylaniline, thus leading to the formation of oligomer products. For more details, see ESI.

[cit15] The reason why a dialkylation product was not formed is not yet clear

[cit16] The DFT calculations showed that in the case of *N*,*N*-dimethyl-*o*-toluidine, the deprotonation of a benzylic C(sp^3^)–H is more favoured than that of a C(sp^2^)–H bond, but the insertion of an alkene into the Y–C bond in the resulting C(sp^3^)–H activation product is more difficult. For more details, see ESI.

[cit17] Simmons E. M., Hartwig J. F. (2012). Angew. Chem., Int. Ed..

[cit18] For DFT calculations on rare-earth-catalysed C–H addition of pyridines to alkenes, see: Luo G. Luo Y. Qu J. Hou Z., Organometallics, 2012, 31 , 3930 .

[cit19] For the X-ray crystal structure of an analogous cationic half-sandwich scandium complex, see: Li X. Nishiura M. Hu L. Mori K. Hou Z., J. Am. Chem. Soc., 2009, 131 , 13870 . 19728718 10.1021/ja9056213

[cit20] Release of SiMe_4_ was observed by ^1^H NMR

[cit21] The analogues of **C**, formed by deprotonation of pyridines with cationic half-sandwich rare earth alkyls, have been previously isolated and structurally characterized (see ref. 10*c* and *e*)

[cit22] Gladysz J. A., Boone B. J. (1997). Angew. Chem., Int. Ed..

