# Supplementary material for: ortho-Selective C–H addition of N,N-dimethyl anilines to alkenes by a yttrium catalyst
Source: Chem Sci. 2016 Apr 26;7(8):5265–70. doi: 10.1039/c6sc00833j (PMC6020524; doi:10.1039/c6sc00833j)
Supplement: Supplementary file 1 [file SC-007-C6SC00833J-s001.pdf]

Electronic Supplementary Material (ESI) for Chemical Science.
This journal is © The Royal Society of Chemistry 2016

Ortho-Selective C–H Addition of *N,N*-Dimethyl Anilines to Alkenes by a Yttrium Catalyst

Guoyong Song,[a,b] Gen Luo,[c] Juzo Oyamada,[a] Yi Luo,*[c] and Zhaomin Hou*[a,c]

[a]Organometallic Chemistry Laboratory and RIKEN Center for Sustainable Resource Science, RIKEN, Wako, Saitama 351-0198, Japan. Email: houz@riken.jp

[b]Beijing Key Laboratory of Lignocellulosic Chemistry, Beijing Forestry University, Beijing 100083, China

[c]State Key Laboratory of Fine Chemicals, School of Pharmaceutical Science and Technology, Dalian University of Technology, Dalian 116024, China. E-mail: luoyi@dlut.edu.cn

Contents

1	General Information	S2
2	Yttrium-catalysed C–H Addition of Tertiary Anilines to alkenes	S3
3	Kinetic Isotope Effect (KIE) Experiment	S10
4	DFT Calculation of C–H Addition of *N,N*-Dimethylaniline to 1-Hexene	S12
5	Energy Profile for the Reaction of *N,N*-Dimethylaniline with Styrene by a Yttrium Catalyst	S16
6	Comparison of the sp^3 and sp^2 C−H Activation of *N,N*-dimethyl-*o*-toluidine and Subsequent Alkene Insertion	S17
7	Optimized Cartesian Coordinates	S18
8	NMR Spectra	S76

1. General Information

All manipulations of air- and moisture-sensitive compounds were performed under a nitrogen atmosphere by use of standard Schlenk techniques or a nitrogen atmosphere in an mBRAUN Labmaster 130 glovebox. Hexane, toluene and THF were obtained from Kanato Chemical Co., purified by an Mbraun SPS-800 Solvent Purification System and dried over fresh sodium chips in a glovebox. All reagents were obtained commercially and distilled from CaH_2 before use. Tertiary anilines were prepared according to the literature procedures[1] and distilled from CaH_2 or sodium before use. Complexes **1-4** were synthesized according to the literature procedures[2] and stored in a glove-box. $[Ph_3C][B(C_6F_5)_4]$ was purchased from Strem and used without purification. Silica gel column chromatography was performed with Silica Gel 60 N (spherical, neutral, 40−50 μm) obtained from Kanato Chemical Co. ^{1}H NMR and ^{13}C NMR spectra of organic products were recorded on a Bruker 500 MHz instrument. Data are reported as follows: chemical shift in ppm (δ), multiplicity (s = singlet, d = doublet, t = triplet, q = quartet, m = multiplet, br = broad signal), coupling constant (Hz), and integration. High-resolution MS were obtained on a Bruker micrOTOF-Q III (ESI$^+$).

Reference

1. a) R. Shi, L. Lu, H. Zhang, B. Chen, Y. Sha, C. Liu, A. Lei. *Angew. Chem., Int. Ed.* **2013**, *52*, 10582. b) X. Huang, J. Huang, C. Du, X. Zhang, F. Song, J. You. *Angew. Chem., Int. Ed.* **2013**, *52*, 12970.
2. a) T. Shima, M. Nishiura, Z. Hou, *Organometallics* **2011**, *30*, 2513; b) M. Nishiura, J. Baldamus, T. Shima, K. Mori, Z. Hou, *Chem. Eur. J.* **2011**, *17*, 5033.

2. Yttrium-Catalyzed C–H Addition of Tertiary Anilines to Alkenes

Representative procedure: In a glovebox, $[Ph_3C][B(C_6F_5)_4]$ (17.4 mg, 0.0189 mmol, 5 mol %) was added to a stirred toluene solution (1.5 mL) of complex **4** ($[C_5Me_4(SiMe_3)Y(CH_2SiMe_3)_2(THF)]$) (10 mg, 0.0189 mmol, 5 mol %) in a Schlenk tube. After 15 min, *N,N*-dimethylaniline (**5a**) (46 mg, 0.38 mmol) and 1-octene (**6a**) (210 mg, 1.9 mmol, 5 equiv) were added. The closed tube was taken outside and heated at 70 °C. After completion of the reaction (monitored by TLC), the mixture was cooled to room temperature and concentrated under reduced pressure. The residue was purified by silica gel column chromatography (hexane/EtOAc), yielding 82.0 mg of **7a** (93% yield) as a colorless oil.

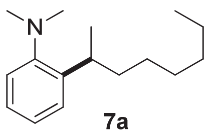

7a

***N,N*-dimethyl-2-(octan-2-yl)aniline (7a)**, colorless oil, 16 h, 93%.

^{1}H NMR (500 MHz, $CDCl_3$) δ 7.22 (d, *J* = 8.1 Hz, 1H), 7.17 – 7.12 (m, 2H), 7.10 – 7.06 (m, 1H), 3.43 – 3.37 (m, 1H), 2.67 (s, 6H), 1.61 – 1.51 (m, 2H), 1.35 – 1.11 (m, 11H), 0.88 (t, *J* = 7.0 Hz, 3H).

^{13}C NMR (126 MHz, $CDCl_3$) δ 152.40, 143.58, 126.63, 126.01, 123.93, 119.69, 45.76, 38.53, 31.84, 31.63, 29.45, 27.78, 22.69, 22.20, 14.09.

HRMS (ESI): calcd for $C_{16}H_{27}N$ $[M + H]^+$ 234.2216, found 234.2212.

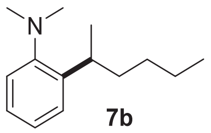

7b

2-(Hexan-2-yl)-*N,N*-dimethylaniline (7b), colorless oil, 18 h, 94%.

^{1}H NMR (500 MHz, $CDCl_3$) δ 7.21 (d, *J* = 7.6 Hz, 1H), 7.19 – 7.12 (m, 2H), 7.10 – 7.05 (m, 1H), 3.44 – 3.36 (m, 1H), 2.67 (s, 6H), 1.57 – 1.53 (m, 2H), 1.33 – 1.22 (m, 3H), 1.21 (d, *J* = 6.9 Hz, 3H), 1.17 – 1.10 (m, 1H), 0.86 (t, *J* = 7.2 Hz, 3H).

^{13}C NMR (126 MHz, $CDCl_3$) δ 152.41, 143.60, 126.63, 126.01, 123.94, 119.70, 45.76, 38.24, 31.64, 30.05, 22.83, 22.21, 14.05.

HRMS (ESI): calcd for $C_{14}H_{24}N$ $[M + H]^+$ 206.1903, found 206.1907.

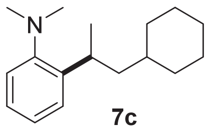

7c

2-(1-Cyclohexylpropan-2-yl)-*N,N*-dimethylaniline (7c), pale yellow oil, 48 h, 86%.

^{1}H NMR (500 MHz, $CDCl_3$) δ 7.21 (d, *J* = 7.5 Hz, 1H), 7.18 – 7.10 (m, 2H), 7.07 (t, *J* = 6.9 Hz, 1H), 3.67 – 3.51 (m, 1H), 2.66 (s, 6H), 1.86 (d, *J* = 12.7 Hz, 1H), 1.70 – 1.51 (m, 5H), 1.48 – 1.36 (m, 2H), 1.23 – 1.04 (m, 7H), 0.92 – 0.77 (m, 2H).

^{13}C NMR (126 MHz, $CDCl_3$) δ 152.40, 143.84, 126.81, 125.99, 123.99, 119.82, 46.81, 45.75, 35.43, 33.69, 33.61, 28.50, 26.77, 26.41, 26.38, 22.46.

HRMS (ESI): calcd for $C_{17}H_{28}N$ $[M + H]^+$ 246.2216, found 246.2217.

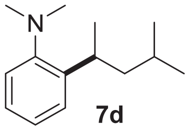

7d

***N,N*-dimethyl-2-(4-methylpentan-2-yl)aniline (7d)**, pale yellow oil, 72 h, 75%.

^{1}H NMR (500 MHz, $CDCl_3$) δ 7.21 (d, *J* = 7.2 Hz, 1H), 7.17 – 7.11 (m, 2H), 7.10 – 7.04 (m, 1H), 3.60 – 3.50 (m, 1H), 2.67 (s, 6H), 1.50 – 1.33 (m, 3H), 1.20 (d, *J* = 6.9 Hz, 3H), 0.90 (d, *J* = 6.2 Hz, 3H), 0.85 (d, *J* = 6.3 Hz, 3H).

^{13}C NMR (126 MHz, $CDCl_3$) δ 152.43, 143.55, 126.80, 125.99, 123.92, 119.78, 48.24, 45.72, 29.31, 25.84, 22.94, 22.62, 22.47.

HRMS (ESI): calcd for $C_{14}H_{24}N$ $[M + H]^+$ 206.1903, found 206.1897.

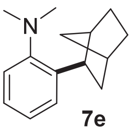

7e

2-(Bicyclo[2.2.1]heptan-2-yl)-N,N-dimethylaniline (7e), Colorless oil, 2.0 equiv norbornene was used in this reaction, 3 h, 99%.

^{1}H NMR (500 MHz, $CDCl_3$) δ 7.32 (dd, *J* = 7.8, 1.2 Hz, 1H), 7.20 – 7.12 (m, 2H), 7.09 – 7.04 (m, 1H), 3.30 – 3.21 (m, 1H), 2.70 (s, 6H), 2.39 (br, 1H), 2.25 (br, 1H), 1.97 – 1.84 (m, 1H), 1.81 – 1.72 (m, 1H), 1.68 – 1.52 (m, 3H), 1.48 – 1.44 (m, 1H), 1.36 – 1.32 (m, 1H), 1.29 – 1.26 (m, 1H).

^{13}C NMR (126 MHz, $CDCl_3$) δ 152.75, 143.59, 126.78, 126.02, 123.64, 119.83, 45.62, 43.52, 41.08, 40.49, 36.88, 36.76, 31.38, 28.63.

HRMS (ESI): calcd for $C_{15}H_{22}N$ $[M + H]^+$ 216.1747, found 216.1749.

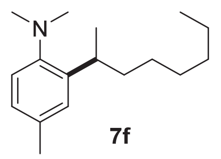

7f

***N,N*,4-trimethyl-2-(octan-2-yl)aniline (7f)**, pale yellow oil, 16 h, 92%

^{1}H NMR (500 MHz, $CDCl_3$) δ: 7.06 (d, *J* = 8.0 Hz, 1H), 7.03 (s, 1H), 6.98 (d, *J* = 8.0 Hz, 1H), 3.43 – 3.36 (m, 1H), 2.65 (s, 6H), 2.33 (s, 3H), 1.65 – 1.50 (m, 2H), 1.30 – 1.18 (m, 11H), 0.89 (t, *J* = 6.7 Hz, 3H).

^{13}C NMR (126 MHz, $CDCl_3$) δ 149.91, 143.51, 133.25, 127.27, 126.67, 119.67, 45.95, 38.48, 31.85, 31.54, 29.46, 27.83, 22.70, 22.20, 21.04, 14.10.

HRMS (ESI): calcd for $C_{17}H_{30}N$ $[M + H]^+$ 248.2373, found 248.2377.

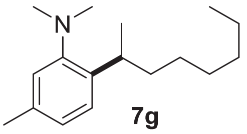

7g

***N,N*,5-trimethyl-2-(octan-2-yl)aniline (7g)**, colorless oil, 12 h, 99%.

^{1}H NMR (500 MHz, $CDCl_3$) δ 7.11 (d, *J* = 7.8 Hz, 1H), 6.95 (s, 1H), 6.90 (d, *J* = 7.7 Hz, 1H), 3.37 – 3.32 (m, 1H), 2.67 (s, 6H), 2.33 (s, 3H), 1.59 – 1.49 (m, 2H), 1.29 – 1.16 (m, 11H), 0.88 (t, *J* = 6.8 Hz, 3H).

^{13}C NMR (126 MHz, $CDCl_3$) δ 152.24, 140.46, 135.44, 126.49, 124.68, 120.42, 45.77, 38.54, 31.85, 31.39, 29.47, 27.82, 22.70, 22.31, 21.13, 14.10.

HRMS (ESI): calcd for $C_{17}H_{30}N$ $[M + H]^+$ 248.2373, found 248.2372.

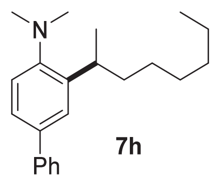

7h

***N,N*-dimethyl-3-(octan-2-yl)-[1,1'-biphenyl]-4-amine (7h)**, pale yellow oil, 18 h, 93%

^{1}H NMR (500 MHz, $CDCl_3$) δ 7.61 (dd, *J* = 8.3, 1.2 Hz, 2H), 7.49 – 7.44 (m, 3H), 7.41 (dd, *J* = 8.2, 2.3 Hz, 1H), 7.34 (t, *J* = 7.4 Hz, 1H), 7.22 (d, *J* = 8.2 Hz, 1H), 3.48 – 3.41 (m, 1H), 2.74 (s, 6H), 1.65 – 1.59 (m, 2H), 1.43 – 1.15 (m, 11H), 0.90 (t, *J* = 7.0 Hz, 3H).

^{13}C NMR (126 MHz, $CDCl_3$) δ 151.73, 143.67, 141.46, 136.52, 128.61, 126.92, 126.66, 125.57, 124.73, 119.81, 45.73, 38.58, 31.83, 31.79, 29.44, 27.80, 22.70, 22.25, 14.13.

HRMS (ESI): calcd for $C_{22}H_{32}N$ $[M + H]^+$ 310.2529, found 310.2535.

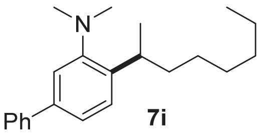

7i

***N,N*-dimethyl-4-(octan-2-yl)-[1,1'-biphenyl]-3-amine (7i)**, 10 h, 94%.

^{1}H NMR (500 MHz, $CDCl_3$) δ 7.67 (d, *J* = 7.2 Hz, 2H), 7.49 (t, *J* = 7.7 Hz, 2H), 7.43 (d, *J* = 1.5 Hz, 1H), 7.41 – 7.37 (m, 2H), 7.35 (d, *J* = 8.0 Hz, 1H), 3.53 – 3.46 (m, 1H), 2.80 (s, 6H), 1.71 – 1.60 (m, 2H), 1.45 – 1.22 (m, 11H), 0.96 (t, *J* = 6.9 Hz, 3H).

^{13}C NMR (126 MHz, $CDCl_3$) δ 152.73, 142.65, 141.46, 139.05, 128.62, 127.08, 126.99, 126.89, 122.68, 118.58, 45.77, 38.55, 31.86, 31.58, 29.46, 27.82, 22.71, 22.20, 14.11.

HRMS (ESI): calcd for $C_{22}H_{32}N$ $[M + H]^+$ 310.2529, found 310.2531.

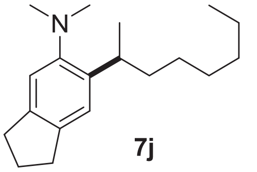

7j

***N,N*-dimethyl-6-(octan-2-yl)-2,3-dihydro-1H-inden-5-amine (7j)**, colorless oil, 48 h, 82%.

^{1}H NMR (500 MHz, $CDCl_3$) δ 7.08 (s, 1H), 7.06 (s, 1H), 3.41 – 3.34 (m, 1H), 2.90 – 2.86 (m, 4H), 2.65 (s, 6H), 2.13 – 1.96 (m, 2H), 1.59 – 1.48 (m, 2H), 1.36 – 1.15 (m, 11H), 0.88 (t, *J* = 7.0 Hz, 3H).

^{13}C NMR (126 MHz, $CDCl_3$) δ 150.81, 141.80, 141.70, 139.69, 122.20, 115.86, 46.12, 38.62, 32.81, 32.57, 31.85, 31.63, 29.50, 27.89, 25.55, 22.71, 22.41, 14.11.

HRMS (ESI): calcd for $C_{19}H_{32}N$ $[M + H]^+$ 274.2529, found 274.2535.

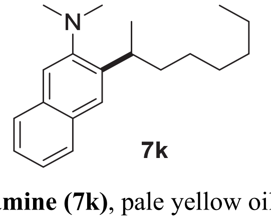

7k

***N,N*-dimethyl-3-(octan-2-yl)naphthalen-2-amine (7k)**, pale yellow oil, 24 h, 86%.

^{1}H NMR (500 MHz, $CDCl_3$) δ 7.78 – 7.74 (m, 2H), 7.68 (s, 1H), 7.47 (s, 1H), 7.42 – 7.36 (m, 2H), 3.53 – 3.46 (m, 1H), 2.81 (s, 6H), 1.74 – 1.64 (m, 2H), 1.43 – 1.18 (m, 11H), 0.91 (t, *J* = 6.8 Hz, 3H).

^{13}C NMR (126 MHz, $CDCl_3$) δ 151.81, 143.85, 132.50, 130.82, 127.04, 126.59, 125.40, 125.06, 124.25, 115.98, 45.73, 39.14, 31.99, 31.85, 29.46, 27.79, 22.69, 22.57, 14.09.

HRMS (ESI): calcd for $C_{20}H_{30}N$ $[M + H]^+$ 284.2373, found 284.2392.

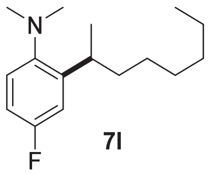

7l

4-Fluoro-*N,N*-dimethyl-2-(octan-2-yl)aniline (7l), colorless oil, 8 mol % of complex **4** and $[Ph_3C][B(C_6F_5)_4]$ were used in this reaction, 48 h, 82%.

^{1}H NMR (500 MHz, $CDCl_3$) δ 7.09 (dd, *J* = 8.7, 5.5 Hz, 1H), 6.87 (dd, *J* = 10.3, 3.0 Hz, 1H), 6.81 (td, *J* = 8.4, 3.0 Hz, 1H), 3.47 – 3.34 (m, 1H), 2.61 (s, 6H), 1.56 – 1.46 (m, 2H), 1.38 – 1.07 (m, 11H), 0.86 (t, *J* = 7.0 Hz, 3H).

^{13}C NMR (126 MHz, $CDCl_3$) δ 159.87 (d, $J_{F\text{-}C}$ = 241.2 Hz), 148.40 (s), 146.29 (d, $J_{F\text{-}C}$ = 6.5 Hz), 121.34 (d, $J_{F\text{-}C}$ = 8.4 Hz), 113.10 (d, $J_{F\text{-}C}$ = 21.6 Hz), 112.43 (d, $J_{F\text{-}C}$ = 21.9 Hz), 46.08, 38.32, 31.90, 31.85, 29.41, 27.68, 22.71, 22.18, 14.13.

HRMS (ESI): calcd for $C_{16}H_{27}FN$ $[M + H]^+$ 284.2373, found 284.2392.

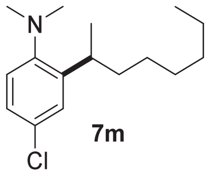

7m

4-Chloro-*N,N*-dimethyl-2-(octan-2-yl)aniline (7m), pale yellow oil, 8 mol % of complex **4** and $[Ph_3C][B(C_6F_5)_4]$ were used in this reaction, 48 h, 75%

^{1}H NMR (500 MHz, $CDCl_3$) δ 7.14 (d, *J* = 2.5 Hz, 1H), 7.09 (dd, *J* = 8.5, 2.5 Hz, 1H), 7.03 (d, *J* = 8.5 Hz, 1H), 3.40 – 3.27 (m, 1H), 2.62 (s, 6H), 1.56 – 1.44 (m, 2H), 1.31 – 1.10 (m, 11H), 0.86 (t, *J* = 7.0 Hz, 3H).

^{13}C NMR (126 MHz, $CDCl_3$) δ 151.03, 145.73, 129.21, 126.83, 126.01, 121.23, 45.69, 38.42, 31.84, 31.82, 29.36, 27.68, 22.68, 22.04, 14.09.

HRMS (ESI): calcd for $C_{16}H_{27}ClN$ $[M + H]^+$ 268.1827, found 268.1831.

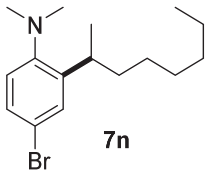

7n

4-Bromo-*N,N*-dimethyl-2-(octan-2-yl)aniline (7n), pale yellow oil, 8 mol % of complex **4** and $[Ph_3C][B(C_6F_5)_4]$ were used in this reaction, 36 h, 85%.

^{1}H NMR (500 MHz, $CDCl_3$) δ 7.28 (d, *J* = 2.4 Hz, 1H), 7.23 (dd, *J* = 8.5, 2.4 Hz, 1H), 6.98 (d, *J* = 8.5 Hz, 1H), 3.37 – 3.30 (m, 1H), 2.63 (s, 6H), 1.53 – 1.45 (m, 2H), 1.31 – 1.10 (m, 11H), 0.87 (t, *J* = 7.0 Hz, 3H).

^{13}C NMR (126 MHz, $CDCl_3$) δ 151.52, 146.13, 129.80, 128.98, 121.65, 117.13, 45.59, 38.43, 31.82, 31.80, 29.33, 27.66, 22.66, 22.01, 14.08.

HRMS (ESI): calcd for $C_{16}H_{27}ClN$ $[M + H]^+$ 268.1827, found 268.1831.

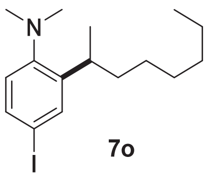

7o

4-Iodo-N,N-dimethyl-2-(octan-2-yl)aniline (7o), pale yellow oil, 8 mol % of complex **4** and $[Ph_3C][B(C_6F_5)_4]$ were used in this reaction, 24 h, 83%.

^{1}H NMR (500 MHz, $CDCl_3$) δ 7.46 (d, *J* = 2.2 Hz, 1H), 7.42 (dd, *J* = 8.4, 2.2 Hz, 1H), 6.85 (d, *J* = 8.4 Hz, 1H), 3.32 – 3.25 (m, 1H), 2.62 (s, 6H), 1.53 – 1.46 (m, 2H), 1.32 – 1.09 (m, 11H), 0.87 (t, *J* = 7.0 Hz, 3H).

^{13}C NMR (126 MHz, $CDCl_3$) δ 152.26, 146.42, 135.86, 135.04, 122.03, 88.18, 45.49, 38.44, 31.79, 31.69, 29.32, 27.66, 22.66, 21.99, 14.09.

HRMS (ESI): calcd for $C_{16}H_{27}IN$ $[M + H]^+$ 360.1183, found 360.1186.

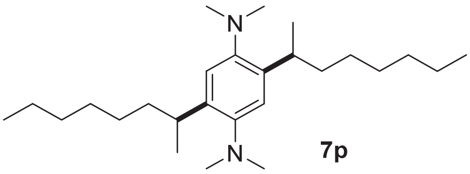

7p

***N^1,N^1,N^4,N^4*-tetramethyl-2,5-di(octan-2-yl)benzene-1,4-diamine (7p)**, pale yellow oil, 16 h, 99%.

^{1}H NMR (500 MHz, $CDCl_3$) δ 6.95 (s, 2H), 3.35 – 3.30 (m, 2H), 2.63 (br, 12H), 1.62 – 1.46 (m, 4H), 1.31 – 1.12 (m, 22H), 0.90 – 0.75 (m, 6H).

^{13}C NMR (126 MHz, $CDCl_3$) δ 148.39, 148.32, 141.08, 140.94, 117.64, 117.54, 46.07, 46.04, 38.55, 38.44, 31.85, 31.78, 31.69, 29.47, 29.42, 27.84, 27.74, 22.69, 22.66, 22.21, 22.13, 14.11.

HRMS (ESI): calcd for $C_{26}H_{49}N_2$ $[M + H]^+$ 389.3890, found 389.3882

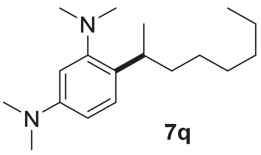

7q

***N^1,N^1,N^3,N^3*-tetramethyl-4-(octan-2-yl)benzene-1,3-diamine (7q)**, pale yellow oil, 24 h, 85%.

^{1}H NMR (500 MHz, C_6D_6) δ 7.18 (d, *J* = 8.5 Hz, 1H), 6.58 (d, *J* = 2.6 Hz, 1H), 6.54 (dd, *J* = 8.5, 2.6 Hz, 1H), 3.67 – 3.40 (m, 1H), 2.62 (s, 6H), 2.60 (s, 6H), 1.74 – 1.59 (m, 2H), 1.43 – 1.20 (m, 11H), 0.86 (t, *J* = 7.0 Hz, 3H).

^{13}C NMR (126 MHz, $CDCl_3$) δ 153.06, 149.29, 131.97, 127.13, 108.94, 104.63, 45.76, 40.91, 38.70, 31.93, 31.07, 29.55, 27.91, 22.76, 22.42, 14.15.

HRMS (ESI): calcd for $C_{18}H_{33}N_2$ $[M + H]^+$ 277.2638, found 277.2641.

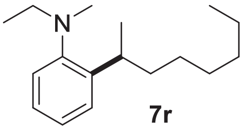

7r

***N*-ethyl-*N*-methyl-2-(octan-2-yl)aniline (7r)**, pale yellow oil, 24 h, 94%.

^{1}H NMR (500 MHz, $CDCl_3$) δ 7.23 (dd, *J* = 7.6, 1.5 Hz, 1H), 7.18 – 7.07 (m, 3H), 3.49 – 3.42 (m, 1H), 2.91 (q, *J* = 7.1 Hz, 2H), 2.65 (s, 3H), 1.57 – 1.53 (m, 2H), 1.34 – 1.13 (m, 11H), 1.07 (t, *J* = 7.1 Hz, 3H), 0.89 (t, *J* = 7.1 Hz, 3H).

^{13}C NMR (126 MHz, $CDCl_3$) δ 151.54, 144.65, 126.51, 125.86, 124.13, 121.14, 51.58, 43.16, 38.47, 31.85, 31.45, 29.49, 27.80, 22.69, 22.19, 14.09, 13.17.

HRMS (ESI): calcd for $C_{17}H_{30}N$ $[M + H]^+$ 248.2373, found 248.2373.

3. Kinetic Isotope Effect (KIE) Experiment

(1) Intramolecular KIE Experiment (eq 2):

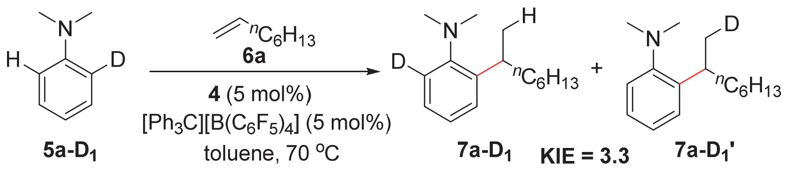

In a glovebox, $[Ph_3C][B(C_6F_5)_4]$ (17.4 mg, 0.0189 mmol, 5 mol %) was added to a stirred toluene solution (1.5 mL) of complex **4** ($[C_5Me_4(SiMe_3)Y(CH_2SiMe_3)_2(THF)]$) (10 mg, 0.0189 mmol, 5 mol %) in a Schlenk tube. After 15 min, **5a-D1** (46 mg, 0.38 mmol) and 1-octene (**6a**) (210 mg, 1.9 mmol, 5 equiv) were added. The closed tube was taken outside and heated at 70 °C for 12 h (58% conversion by GC-MS). The mixture was cooled to room temperature and concentrated under reduced pressure, and **7a-D$_1$** and **7a-D$_1$'** was isolated by silica gel column. The KIE value was determined as 3.4 by analysis of relative ratio of **7a-D$_1$** and **7a-D$_1$'** by ^{1}H NMR.

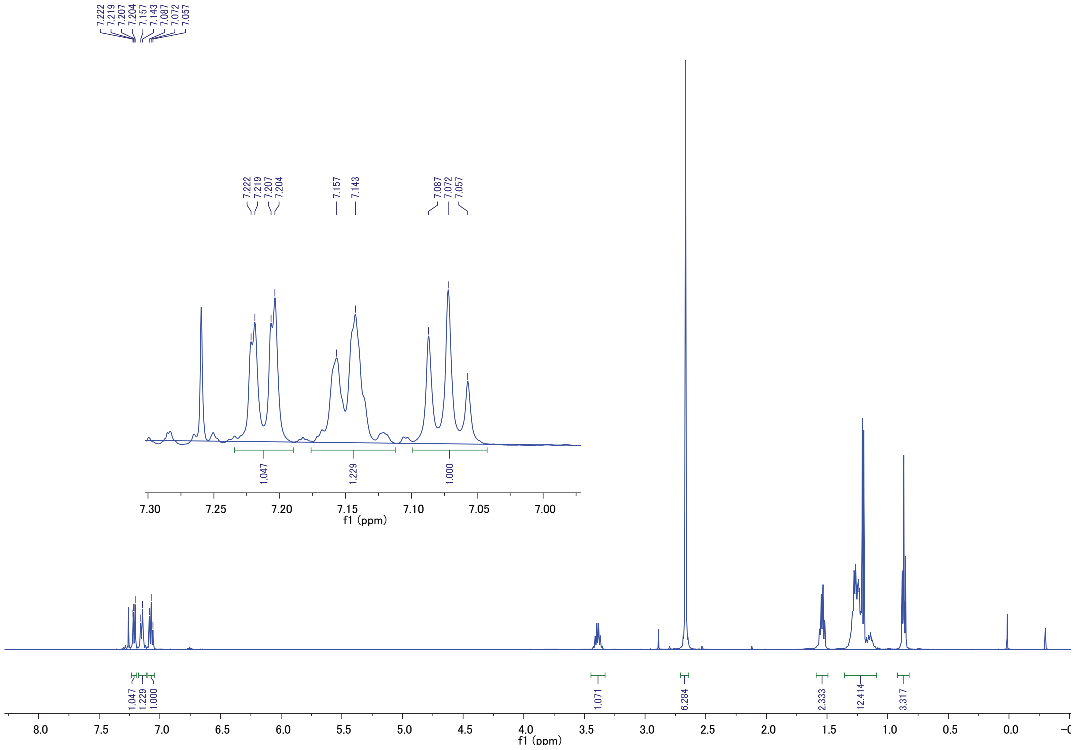

Figure S1. ^{1}H NMR spectra of the mixture of **7a-D$_1$** and **7a-D$_1$'**

(2) Determination of Kinetic Isotope Effect by Initial Rate of two Side-by-Side Reactions (eq 3)

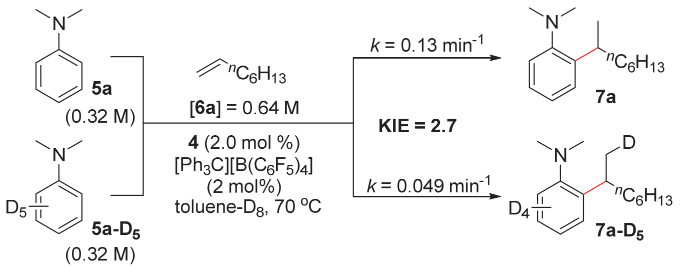

In a glove-box, *N,N*-dimethylaniline (**5a**) (25 μL, 0.2 mmol), 1-octene (**6a**) (60 μL, 0.4 mmol) and toluene-D_8 (0.4 mL) were injected to a J-Young NMR tube, followed by addition of the solution of **4**/[Ph_3C][$B(C_6F_5)_4$] in toluene-D_8 (0.04 M, 0.1 mL, 0.004 mmol, 2 mol %). The NMR tube was taken outside and was analyzed on an NMR spectrometer at 70 °C. The signal of methyl specie (δ: 2.49 ppm) in compound **7a** was used to calculated formation of **7a**. The slope of the plot of [**7a**] against *t* (min) give the rate constant $k_{C\text{-}H}$ as **0.13 min^{-1}**. $k_{C\text{-}D}$ was determined as **0.049 min^{-1}** under same reaction conditions by using **5a-D$_5$** as a substrate.

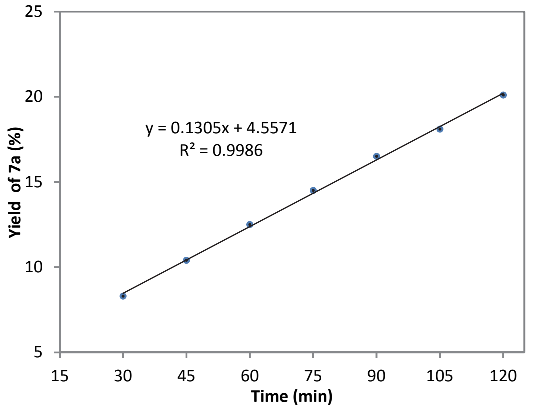

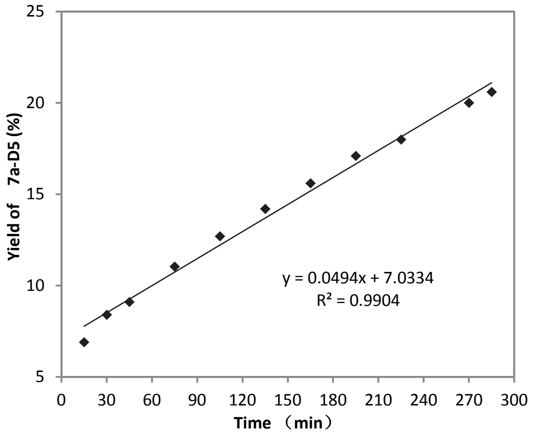

Figure S2. Kinetic plot for yttrium-catalyzed C–H Addition of **5a** or **5a-D** to 1-hexene

4. DFT Calculation of C–H Addition of *N,N*-dimethylaniline to 1-Hexene

Computational Details

All calculations were performed with Gaussian 09 program.[3] The B3PW91 functional[4] was used for geometry optimizations with the 6-31G(d) basis set for C, H, O, and N atoms and Stuttgart/Dresden relativistic effective core potentials (RECPs)[5] as well as the associated valence basis sets were used for Y atom. Each optimized structure was subsequently analyzed by harmonic vibrational frequencies at the same level of theory for characterization of a minimum (N_{Imag} = 0) or a transition state (N_{Imag} = 1). To obtain more reliable relative energies, single-point energy calculations were carried out with a larger basis set. In such single-point energies, the M06-L functional, which often shows better performance in the treatment of transition-metal systems,[6] was used together with the CPCM model[7] (in toluene solution) for considering the solvation effect, the Stuttgart/Dresden ECP together with associated basis sets were used for Y atom and the 6-311+G(d,p) was used for the remaining atoms. Since the free energies obtained from gas-phase calculation often overestimate the translational entropy in comparison with that in solution. Therefore, such a destabilization energy was partly due to the larger decrease in translation entropy calculated in gas-phase. The method of Whitesides[8] was applied to estimate the decrease in translational entropy in this work. In the system investigated, we may assume that the free volume of the solution is dominated by the free volume of the solvent (toluene used in experiment). We also assume that the molecules are cubic and are in 3D cubic array. According to the Whitesides method, the translational entropy of an analyte in solution can be therefore estimated by the following equation:

$$S_{trans}^{analyte} = R\ln\left[\left(\frac{10^{-15/2}V_{free}^{solvent}}{N_A^4[X]}\right)\left(\frac{2\pi MRTe^{5/3}}{h^2}\right)^{3/2}\right]$$
$$= 11.1 + 12.5\ln(T) + 12.5\ln(M) + 8.3\ln V_{free}^{solvent}$$

in which

$$V_{free}^{solvent} = 8\left(\sqrt[3]{\left(\frac{10^{27}}{[X]N_A}\right)} - \sqrt[3]{V_{molec}}\right)^3$$

where, the temperature (T, K), mass (M, g/mol) and concentration (X, mol/L) of the analyte, Plank's constant (h, J s^2), the fundamental constant (e, unitless), and Avogadro's number (N_A, unitless) are included. The numerical term $10^{-15/2}$ converts units of m^3 and kg into L and g, respectively. The temperature of 343.15 K was used for estimating entropy. The volume of one toluene molecule (V_{molec}) was computed to be 138.8 $Å^3$ via Gaussian program, and the V_{free} was further computed to be 0.65 $Å^3$ for toluene solvent.

Reference

3. Frisch, M. J.; Trucks, G. W.; Schlegel, H. B.; Scuseria, G. E.; Robb, M. A.; Cheeseman, J. R.; Scalmani, G.; Barone, V.; Mennucci, B.; Petersson, G. A.; Nakatsuji, H.; Caricato, M.; Li, X.; Hratchian, H. P.; Izmaylov, A. F.;

Bloino, J.; Zheng, G.; Sonnenberg, J. L.; Hada, M.; Ehara, M.; Toyota, K.; Fukuda, R.; Hasegawa, J.; Ishida, M.; Nakajima, T.; Honda, Y.; Kitao, O.; Nakai, H.; Vreven, T.; Montgomery, J. A. Jr.; Peralta, J. E.; Ogliaro, F.; Bearpark, M.; Heyd, J. J.; Brothers, E.; Kudin, K. N.; Staroverov, V. N.; Kobayashi, R.; Normand, J.; Raghavachari, K.; Rendell, A.; Burant, J. C.; Iyengar, S. S.; Tomasi, J.; Cossi, M.; Rega, N.; Millam, N. J.; Klene, M.; Knox, J. E.; Cross, J. B.; Bakken, V.; Adamo, C.; Jaramillo, J.; Gomperts, R.; Stratmann, R. E.; Yazyev, O.; Austin, A. J.; Cammi, R.; Pomelli, C.; Ochterski, J. W.; Martin, R. L.; Morokuma, K.; Zakrzewski, V. G.; Voth, G. A.; Salvador, P.; Dannenberg, J. J.; Dapprich, S.; Daniels, A. D.; Farkas, Ö.; Foresman, J. B.; Ortiz, J. V.; Cioslowski, J.; Fox, D. J. *Gaussian 09, Revision A.01*; Gaussian, Inc.: Wallingford, CT, 2009.

4. (a) Beck, A. D. *J. Chem. Phys.* **1993**, *98*, 5648−5652. (b) Lee, C. T.; Yang, W. T.; Parr, R. G. *Phys. Rev. B* **1988**, *37*, 785−789. (c) Perdew, J. P.; Burke, K.; Wang, Y. *Phys. Rev. B* **1996**, *54*, 16533−16539.
5. (a) Dolg, M.; Wedig, U.; Stoll, H.; Preuss, H. *J. Chem. Phys.* **1987**, *86*, 866−872. (b) Schwerdtfeger, P.; Dolg, M.; Schwarz, W. H. E.; Bowmaker, G. A.; Boyd, P. D. W. *J. Chem. Phys*. **1989**, *91*, 1762−1774. (c) Dolg, M.; Stoll, H.; Savin, A.; Preuss, H. *Theor. Chim. Acta* **1989**, *75*, 173–194. (d) Andrae, D.; Haeussermann, U.; Dolg, M.; Stoll, H.; Preuss, H. *Theor. Chim. Acta* **1990**, *77*, 123–141. (e) Dolg, M.; Stoll, H.; Preuss, H. *Theor. Chim. Acta* **1993**, *85*, 441–450. (f) Bergner, A.; Dolg, M.; Kuechle, W.; Stoll, H.; Preuss, H. *Mol. Phys.* **1993**, *80*, 1431–1441.
6. (a) Averkiev, B. B.; Truhlar, D. G. *Catal. Sci. Technol.* **2011**, *1*, 1526−1529. (b) Gusev, D. G. *Organometallics* **2013**, *32*, 4239–4243. (c) Zhao Y.; Truhlar, D. G. *Acc. Chem. Res.* **2007**, *41*, 157–167.
7. (a) Barone, V.; Cossi, M. *J. Phys. Chem. A* **1998**, *102*, 1995−2001. (b) Cossi, M.; Rega, N.; Scalmani, G.; Barone, V. *J. Comput. Chem.* **2003**, *24*, 669−681.
8. Mammen, M.; Shakhovich, E. I.; Deutch, J. M.; Whitesides, G. M. *J. Org. Chem*. **1998**, *63*, 3821–3830.

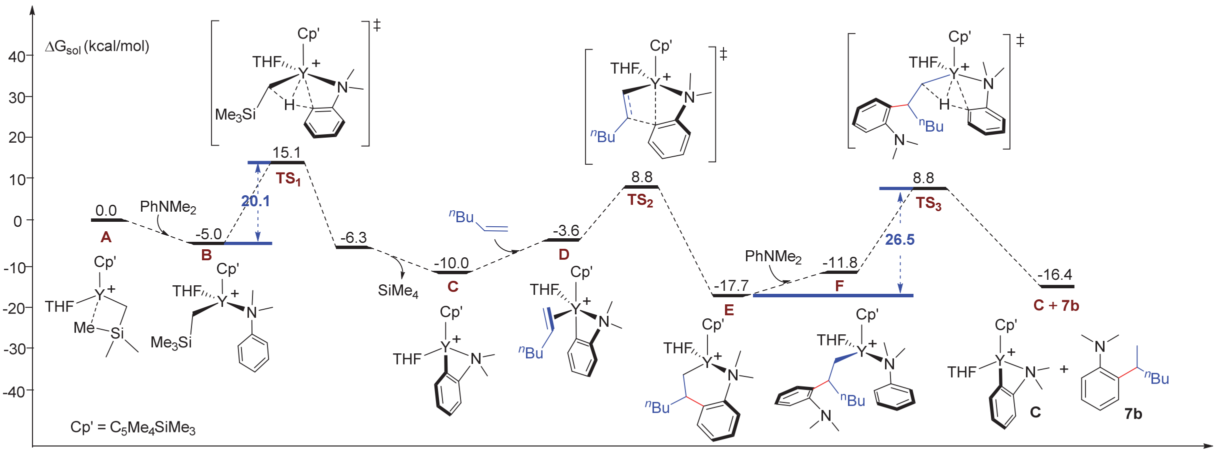

Figure S3. Energy profile (free energy in toluene solution) of C–H addition of *N,N*-dimethylaniline to 1-Hexene catalyzed by **4** (***THF-ligated***). The energies are relative to **A** and all other species involved in the corresponding reaction.

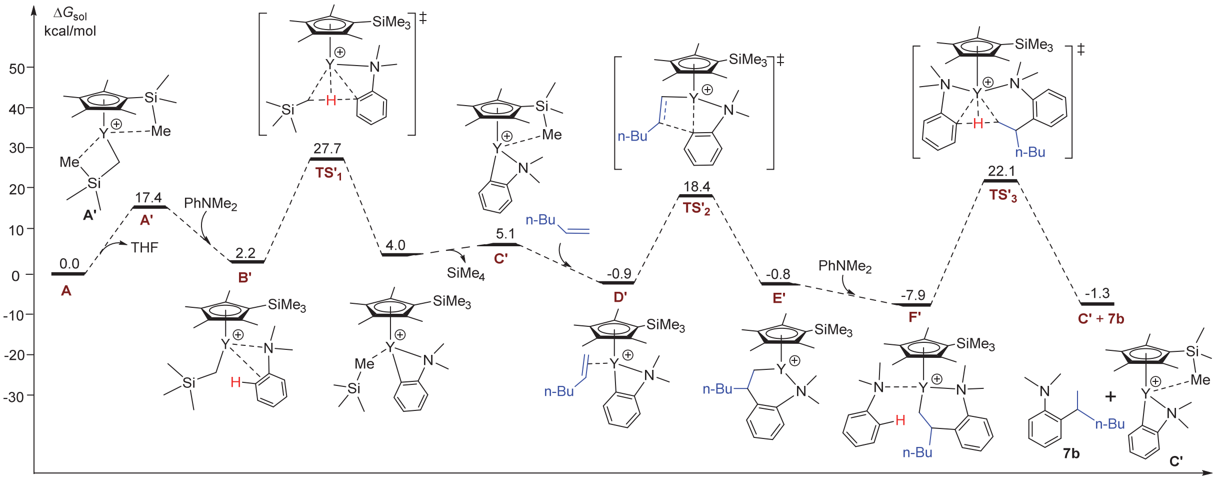

Figure S4. Energy profile (free energy in toluene solution) of C–H addition of *N,N*-dimethylaniline to 1-Hexene catalyzed by **4** (***THF-dissociated***). The energies are relative to **A** and all other species involved in the corresponding reaction.

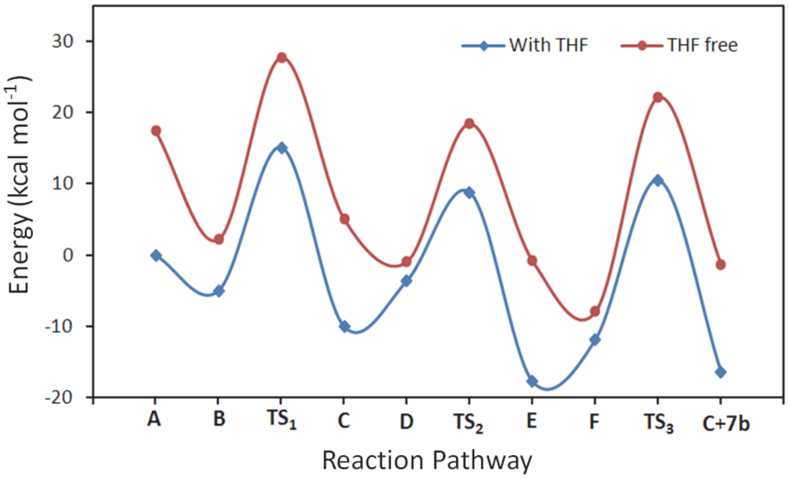

Figure S5. Comparison of energy profiles for the THF-ligated and THF-dissociated pathways.

As shown in Figure S5, it is clear that the THF-ligated pathway has a lower energy profile than that of THF-dissociated.

5. Energy profile for the Reaction of *N,N*-Dimethylaniline with Styrene by a Yttrium Catalyst

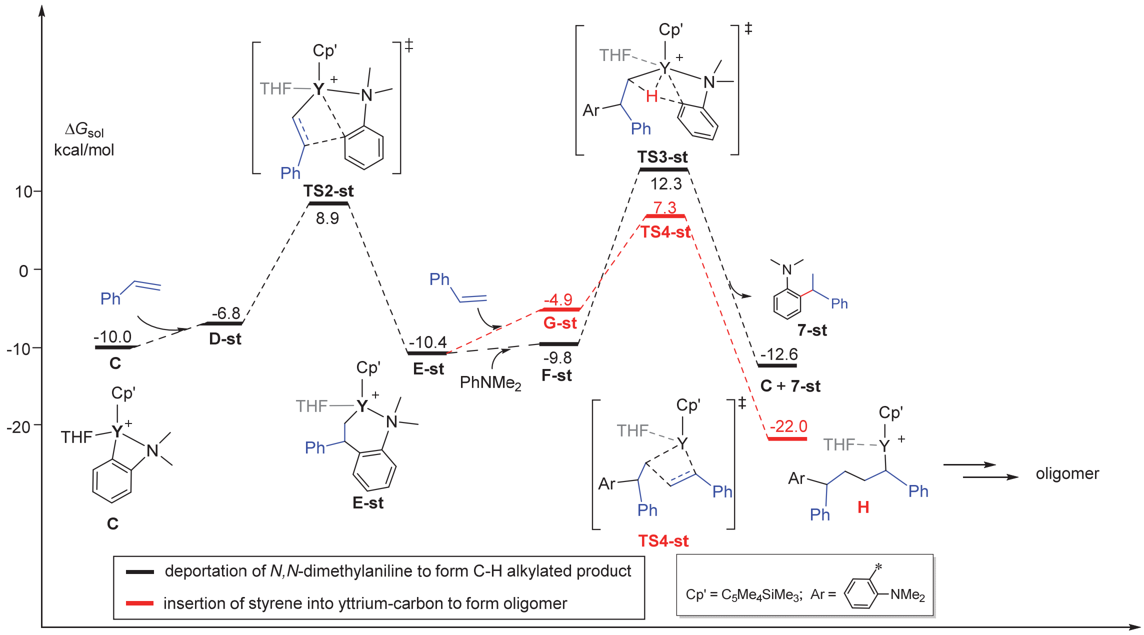

Figure S6 Comparison of energy profiles for the insertion of styrene into yttrium-carbon to form oligomer and deportation of *N,N*-dimethylaniline to form C-H alkylated product. The energy values are relative to **A**.

6 Comparison of the sp^3 and sp^2 C−H Activation of *N,N*-dimethyl-*o*-toluidine and Subsequent Alkene Insertion

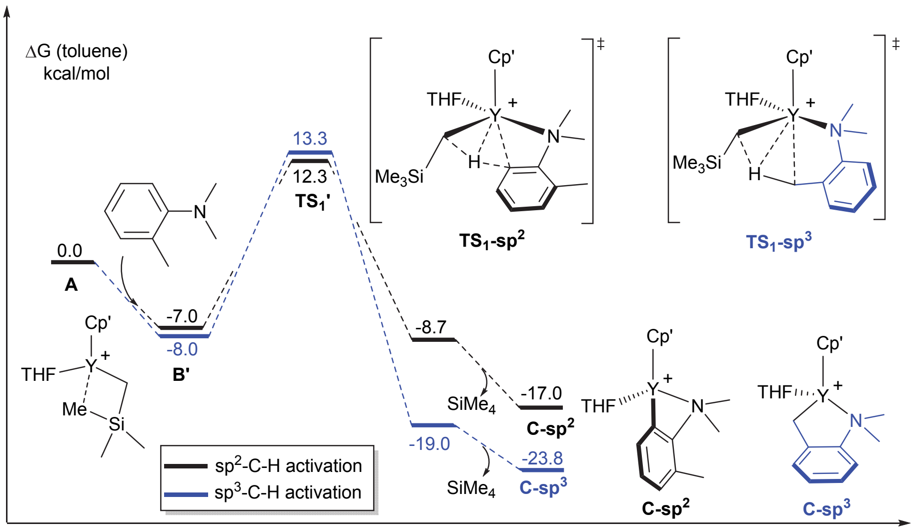

Figure S7. Energy profile for sp^2 and sp^3 C−H activation of *N,N*-dimethyl-*o*-toluidine. Cp' = $C_5Me_4SiMe_3$. The energy values are relative to **A**.

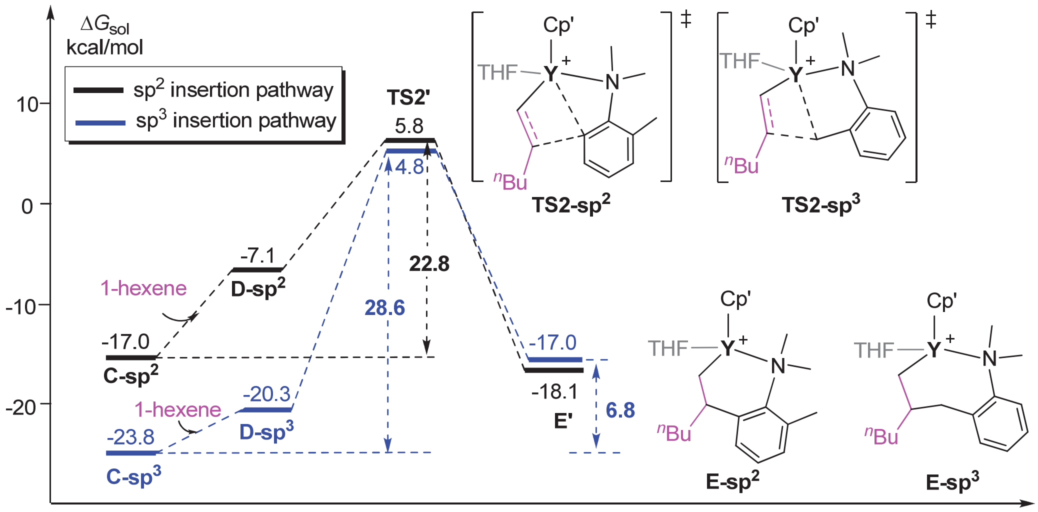

Figure S8. Energy profile for insertion of 1-hexene into yttrium-carbon bond of **C-sp^2** and **C-sp^3**. Cp' = $C_5Me_4SiMe_3$. The energy values are relative to **A**.

7. Optimized Cartesian Coordinates

$PhNMe_2$ (5a)

N	-1.570040000	0.000011000	-0.136719000
C	0.548079000	-1.206170000	-0.027696000
C	-0.184489000	-0.000012000	-0.062281000
C	0.548083000	1.206173000	-0.027705000
H	0.032853000	2.160362000	-0.032761000
C	1.938442000	1.196800000	0.014339000
H	2.467882000	2.146770000	0.037528000
C	2.652137000	-0.000002000	0.033355000
H	3.737824000	0.000018000	0.068564000
C	1.938464000	-1.196789000	0.014388000
H	2.467883000	-2.146769000	0.037612000
C	-2.286702000	1.241106000	0.053734000
H	-2.111866000	1.692875000	1.044375000
H	-3.358099000	1.055743000	-0.051652000
H	-2.006500000	1.978696000	-0.708669000
C	-2.286710000	-1.241112000	0.053683000
H	-2.006483000	-1.978661000	-0.708749000
H	-3.358112000	-1.055766000	-0.051657000
H	-2.111828000	-1.692921000	1.044299000
H	0.032905000	-2.160389000	-0.032761000

A

Y	-0.462543000	-0.020522000	0.148141000
C	-1.723463000	-0.624950000	-1.725843000
H	-1.796314000	0.201797000	-2.451633000
H	-1.469157000	-1.528926000	-2.290692000
C	1.969099000	-0.793742000	0.106226000
C	1.676885000	-0.581177000	1.500866000
C	0.733887000	-1.560201000	1.924434000
C	0.454594000	-2.417032000	0.811115000
C	1.211259000	-1.958130000	-0.298505000
C	2.305922000	0.425166000	2.426322000
H	3.236132000	0.026083000	2.852292000
H	2.567997000	1.360196000	1.925193000
H	1.655319000	0.662429000	3.275660000
C	0.246608000	-1.767808000	3.332355000
H	0.962089000	-2.375959000	3.901413000
H	0.125758000	-0.827506000	3.882459000
H	-0.711962000	-2.296330000	3.366608000
C	-0.372726000	-3.669831000	0.860053000
H	0.242197000	-4.522772000	1.176999000
H	-1.202444000	-3.597550000	1.571765000
H	-0.792182000	-3.925414000	-0.117726000
C	1.307643000	-2.679439000	-1.613396000
H	2.104294000	-3.434025000	-1.576184000
H	0.379870000	-3.204369000	-1.858858000
H	1.532303000	-2.009506000	-2.447412000
Si	-3.330377000	-0.829417000	-0.790891000
C	-4.009235000	-2.584331000	-0.796421000
H	-4.303891000	-2.868079000	-1.813608000
H	-3.265636000	-3.312929000	-0.454451000
H	-4.893409000	-2.679521000	-0.155173000
C	-4.655493000	0.436142000	-1.240312000
H	-5.528650000	0.362874000	-0.581102000
H	-4.281661000	1.466287000	-1.184214000
H	-5.000274000	0.270533000	-2.267788000
C	-2.951477000	-0.438896000	1.104573000
H	-2.222563000	-1.079956000	1.640184000
H	-2.776943000	0.621619000	1.363524000
H	-3.889808000	-0.676318000	1.621139000
C	-0.400193000	3.188154000	1.431703000

O	-0.571564000	2.346208000	0.241589000
C	-0.920065000	3.189069000	-0.910396000
C	-1.331732000	4.520050000	-0.307816000
C	-0.416574000	4.618934000	0.915597000
H	-1.241089000	2.984161000	2.103903000
H	0.535755000	2.897863000	1.914082000
H	-0.028560000	3.278359000	-1.539220000
H	-1.710486000	2.679643000	-1.466417000
H	-1.192818000	5.344208000	-1.012475000
H	-2.385289000	4.501002000	-0.007360000
H	0.590377000	4.934564000	0.621621000
H	-0.785418000	5.317778000	1.670891000
Si	3.249031000	0.058184000	-1.016698000
C	4.506835000	-1.211532000	-1.617268000
H	4.066365000	-1.981384000	-2.258249000
H	5.291883000	-0.709950000	-2.196466000
H	4.990170000	-1.716000000	-0.772449000
C	4.197272000	1.442670000	-0.146832000
H	4.735155000	1.097975000	0.742608000
H	4.948254000	1.828592000	-0.847984000
H	3.566535000	2.291886000	0.141802000
C	2.360526000	0.854740000	-2.495784000
H	3.098472000	1.219427000	-3.221477000
H	1.692414000	0.170726000	-3.031484000
H	1.770310000	1.729307000	-2.187706000

B

Y	0.065313000	-0.183609000	-0.195061000
C	-0.132034000	2.148792000	-0.510103000
H	0.865071000	2.498693000	-0.178703000
H	-0.046483000	2.176088000	-1.616866000
C	2.695303000	-0.487499000	-0.305863000
C	2.168501000	-1.828479000	-0.390838000
C	1.458863000	-1.966498000	-1.614895000
C	1.551864000	-0.728839000	-2.321226000
C	2.316826000	0.171531000	-1.532299000
C	2.486327000	-3.002537000	0.494916000
H	3.333602000	-3.564800000	0.078798000
H	2.767419000	-2.711245000	1.506865000
H	1.652809000	-3.711825000	0.563912000
C	0.887767000	-3.246484000	-2.156746000
H	1.673571000	-3.850411000	-2.630631000
H	0.440345000	-3.874458000	-1.377326000
H	0.125195000	-3.065391000	-2.921341000
C	1.098194000	-0.478816000	-3.731490000
H	1.908798000	-0.699387000	-4.439302000
H	0.251505000	-1.111654000	-4.018134000
H	0.814114000	0.566214000	-3.899338000
C	2.803752000	1.501255000	-2.033909000
H	3.673650000	1.360880000	-2.689370000
H	2.044394000	2.030408000	-2.618818000
H	3.108368000	2.169153000	-1.225876000
Si	-1.294383000	3.578581000	-0.117437000
C	-0.658122000	5.170645000	-0.921896000
H	0.329630000	5.446576000	-0.532474000
H	-0.562641000	5.058669000	-2.009080000
H	-1.333699000	6.014603000	-0.734708000
C	-1.434871000	3.920523000	1.746540000
H	-1.955865000	4.873221000	1.903531000
H	-2.002214000	3.162048000	2.300897000
H	-0.447956000	4.018703000	2.216499000
C	-3.043095000	3.271957000	-0.794909000
H	-3.033883000	3.182326000	-1.888772000
H	-3.509893000	2.366536000	-0.386796000

H	-3.700926000	4.114660000	-0.549554000
C	-2.402827000	-0.123593000	-2.514020000
O	-1.821125000	-0.911320000	-1.426068000
C	-2.578719000	-2.149954000	-1.251600000
C	-3.800915000	-2.001895000	-2.146269000
C	-3.283686000	-1.101554000	-3.270818000
H	-2.977812000	0.691830000	-2.067143000
H	-1.579331000	0.288821000	-3.099666000
H	-1.934709000	-2.976589000	-1.566724000
H	-2.820952000	-2.249744000	-0.190824000
H	-4.163317000	-2.969801000	-2.502910000
H	-4.615102000	-1.510226000	-1.603017000
H	-2.694000000	-1.679032000	-3.991747000
H	-4.084263000	-0.592310000	-3.814168000
Si	4.058223000	0.198434000	0.835437000
C	5.643152000	0.341938000	-0.180865000
H	5.545246000	1.043790000	-1.015538000
H	6.469338000	0.692398000	0.449940000
H	5.932504000	-0.630233000	-0.597056000
C	4.453571000	-0.914587000	2.316449000
H	4.839997000	-1.894986000	2.019277000
H	5.245284000	-0.421392000	2.895099000
H	3.615294000	-1.070523000	3.005897000
C	3.623219000	1.899707000	1.552858000
H	4.443482000	2.226521000	2.204721000
H	3.490796000	2.676041000	0.792243000
H	2.714436000	1.883988000	2.165993000
N	-0.392051000	-0.666969000	2.301628000
C	-2.649173000	0.096984000	1.706088000
C	-1.824252000	-0.905831000	2.239965000
C	-2.407628000	-2.106604000	2.666259000
H	-1.809252000	-2.899995000	3.097888000
C	-3.788224000	-2.288376000	2.568911000
H	-4.223218000	-3.219869000	2.920324000
C	-4.604664000	-1.286782000	2.048834000
H	-5.679790000	-1.428932000	1.992520000
C	-4.026650000	-0.093910000	1.616404000
H	-4.648329000	0.704638000	1.221389000
C	0.365490000	-1.780970000	2.896742000
H	0.066354000	-1.957881000	3.939171000
H	1.424797000	-1.525952000	2.877671000
H	0.223785000	-2.698146000	2.321825000
C	-0.073099000	0.572449000	3.059001000
H	-0.503862000	1.451103000	2.578707000
H	1.012331000	0.692995000	3.087755000
H	-0.453044000	0.504838000	4.086807000
H	-2.228418000	1.045491000	1.379350000

TS1, Imaginary frequency = 1364.4i

Y	-0.032875000	0.474837000	0.024148000
C	-0.307938000	-1.889315000	0.737743000
H	0.725996000	-2.082680000	0.397797000
H	-0.212374000	-1.544431000	1.785485000
C	2.610843000	0.548860000	0.229232000
C	2.222060000	1.866921000	-0.211506000
C	1.486370000	2.500639000	0.827311000
C	1.431502000	1.605472000	1.938947000
C	2.120715000	0.416267000	1.579675000
C	2.696369000	2.599622000	-1.436353000
H	3.639389000	3.119764000	-1.219432000
H	2.889835000	1.940364000	-2.284055000
H	1.986263000	3.369118000	-1.757636000
C	1.015069000	3.928400000	0.831756000
H	1.828827000	4.605606000	1.124895000

H	0.669037000	4.264854000	-0.152659000
H	0.200057000	4.088477000	1.545210000
C	0.905562000	1.946275000	3.303799000
H	1.666008000	2.486039000	3.884060000
H	0.023090000	2.593564000	3.259871000
H	0.645247000	1.054477000	3.883906000
C	2.430266000	-0.697907000	2.540266000
H	3.341986000	-0.469063000	3.107226000
H	1.633088000	-0.848409000	3.276559000
H	2.589998000	-1.654652000	2.037476000
Si	-1.098269000	-3.615505000	0.873113000
C	0.136725000	-4.794224000	1.683351000
H	1.049763000	-4.895167000	1.084123000
H	0.429670000	-4.448748000	2.682283000
H	-0.292554000	-5.797497000	1.795444000
C	-1.543238000	-4.268138000	-0.843381000
H	-1.999410000	-5.262795000	-0.769305000
H	-2.254207000	-3.616815000	-1.364623000
H	-0.652198000	-4.370824000	-1.475774000
C	-2.638288000	-3.529700000	1.970279000
H	-2.396130000	-3.135500000	2.965331000
H	-3.437902000	-2.911158000	1.547053000
H	-3.053130000	-4.534670000	2.114396000
C	-2.465897000	1.042868000	2.349527000
O	-1.784439000	1.606025000	1.191012000
C	-2.628969000	2.622687000	0.567471000
C	-3.932188000	2.616816000	1.359025000
C	-3.498071000	2.089088000	2.729305000
H	-2.931605000	0.096996000	2.051374000
H	-1.708913000	0.856611000	3.113819000
H	-2.095220000	3.574857000	0.644148000
H	-2.767927000	2.356196000	-0.484722000

H	-4.383348000	3.611491000	1.405990000
H	-4.653349000	1.934878000	0.897168000
H	-3.038642000	2.883047000	3.328646000
H	-4.324389000	1.661003000	3.303242000
Si	3.936831000	-0.602130000	-0.511861000
C	5.443779000	-0.558124000	0.626198000
H	5.237159000	-0.979669000	1.615437000
H	6.264081000	-1.139463000	0.187420000
H	5.803218000	0.467692000	0.768464000
C	4.522584000	-0.078545000	-2.233149000
H	5.001820000	0.905739000	-2.239379000
H	5.277933000	-0.805382000	-2.558517000
H	3.734562000	-0.080977000	-2.995357000
C	3.350131000	-2.397211000	-0.683452000
H	4.178412000	-2.997566000	-1.081274000
H	3.052593000	-2.856104000	0.265379000
H	2.514656000	-2.501960000	-1.386075000
N	-0.340460000	0.405204000	-2.486610000
C	-2.085358000	-0.513660000	-1.089753000
C	-1.768138000	0.178232000	-2.270535000
C	-2.752677000	0.633552000	-3.155061000
H	-2.499708000	1.179646000	-4.058616000
C	-4.090857000	0.362364000	-2.873041000
H	-4.861116000	0.697500000	-3.562308000
C	-4.438589000	-0.346591000	-1.722017000
H	-5.482733000	-0.568658000	-1.516806000
C	-3.441194000	-0.768842000	-0.843442000
H	-3.727175000	-1.319478000	0.050912000
C	-0.036089000	1.543815000	-3.366989000
H	-0.357912000	1.356508000	-4.400109000
H	1.040777000	1.712610000	-3.373334000
H	-0.534505000	2.445852000	-3.001113000

C	0.303333000	-0.818017000	-3.022709000
H	0.087751000	-1.670530000	-2.374305000
H	1.384756000	-0.666371000	-3.077133000
H	-0.077484000	-1.046593000	-4.027898000
H	-1.239928000	-1.263737000	-0.218812000

SiMe4

Si	0.000209000	0.000056000	0.000304000
C	1.657039000	0.691424000	0.600086000
H	1.611472000	1.779139000	0.733585000
H	1.948361000	0.252460000	1.562125000
H	2.460228000	0.479826000	-0.116209000
C	-0.449141000	0.789971000	-1.660196000
H	0.313815000	0.582735000	-2.420350000
H	-1.404917000	0.407444000	-2.038662000
H	-0.540836000	1.879467000	-1.572640000
C	0.138420000	-1.875923000	-0.210769000
H	0.403339000	-2.365460000	0.734302000
H	-0.808781000	-2.309938000	-0.553549000
H	0.908012000	-2.139662000	-0.946434000
C	-1.346532000	0.394474000	1.270625000
H	-1.455866000	1.476443000	1.413086000
H	-2.320805000	0.002876000	0.953721000
H	-1.115660000	-0.045788000	2.248283000

C

Y	0.168640000	-1.271570000	-0.420926000
C	2.473537000	-0.131314000	-0.396075000
C	2.593976000	-1.197708000	-1.358233000
C	1.785001000	-0.890380000	-2.488821000
C	1.158511000	0.372172000	-2.252518000
C	1.581684000	0.845017000	-0.979464000
C	3.482463000	-2.410637000	-1.260220000
H	4.524618000	-2.148750000	-1.485271000
H	3.482764000	-2.864190000	-0.263989000
H	3.194203000	-3.184537000	-1.979535000
C	1.718592000	-1.667235000	-3.774438000
H	2.522220000	-1.355830000	-4.455008000
H	1.836765000	-2.746592000	-3.626307000
H	0.776277000	-1.502743000	-4.308454000
C	0.297198000	1.113210000	-3.236832000
H	0.912568000	1.724784000	-3.909934000
H	-0.284687000	0.435372000	-3.871933000
H	-0.405483000	1.793152000	-2.744339000
C	1.271783000	2.211186000	-0.433590000
H	2.100735000	2.902467000	-0.636804000
H	0.380575000	2.640517000	-0.901166000
H	1.101331000	2.203102000	0.644536000
C	-2.796091000	-1.496409000	-1.967306000
O	-1.516931000	-2.200336000	-1.794934000
C	-1.508429000	-3.417920000	-2.604935000
C	-2.954954000	-3.620816000	-3.026664000
C	-3.463045000	-2.181882000	-3.148674000
H	-3.362489000	-1.608888000	-1.037967000
H	-2.575831000	-0.438676000	-2.128835000
H	-0.847146000	-3.248489000	-3.460039000
H	-1.108733000	-4.228398000	-1.988306000
H	-3.030918000	-4.182895000	-3.961173000
H	-3.510644000	-4.164524000	-2.254584000
H	-3.134596000	-1.734077000	-4.093067000
H	-4.552501000	-2.107472000	-3.097453000
Si	3.600051000	0.210054000	1.100331000
C	5.010893000	1.317183000	0.510270000

H	4.642363000	2.265177000	0.102306000
H	5.685733000	1.554098000	1.342004000
H	5.603972000	0.827724000	-0.270990000
C	4.364042000	-1.368691000	1.817399000
H	5.082241000	-1.837411000	1.137045000
H	4.917765000	-1.092606000	2.723929000
H	3.630207000	-2.128088000	2.112557000
C	2.670496000	1.080577000	2.505446000
H	3.203923000	0.915738000	3.450029000
H	2.626825000	2.164016000	2.350343000
H	1.641024000	0.728027000	2.640433000
N	-0.463910000	-2.337824000	1.738391000
C	-1.174390000	-0.088340000	1.062234000
C	-1.249126000	-1.107760000	2.028592000
C	-1.993260000	-0.998564000	3.208166000
H	-2.025498000	-1.800747000	3.942271000
C	-2.701919000	0.177118000	3.438100000
H	-3.286868000	0.285184000	4.347550000
C	-2.657574000	1.218064000	2.504624000
H	-3.211350000	2.134291000	2.694636000
C	-1.902995000	1.082718000	1.341326000
H	-1.878871000	1.914120000	0.638230000
C	-1.327447000	-3.530714000	1.628227000
H	-1.808592000	-3.774229000	2.584882000
H	-0.726746000	-4.394379000	1.320692000
H	-2.113213000	-3.353290000	0.888551000
C	0.581788000	-2.573215000	2.755352000
H	1.206182000	-1.682962000	2.856269000
H	1.206217000	-3.420524000	2.452088000
H	0.145373000	-2.801040000	3.736926000

***n*-hexene (6b)**

C	-2.037663000	-0.205527000	0.388975000
H	-2.075620000	-0.863167000	1.260261000
C	-3.081777000	-0.173408000	-0.439610000
H	-3.090219000	0.464038000	-1.322381000
H	-3.968733000	-0.778303000	-0.268069000
C	-0.773530000	0.585587000	0.224341000
H	-0.859337000	1.242097000	-0.652058000
H	-0.640943000	1.244842000	1.096781000
C	0.473196000	-0.300361000	0.087930000
H	0.538454000	-0.972760000	0.956275000
H	0.356890000	-0.949445000	-0.791303000
C	1.770978000	0.499669000	-0.031151000
H	1.703217000	1.172905000	-0.897669000
H	1.877876000	1.149165000	0.849638000
C	3.009582000	-0.383290000	-0.167547000
H	3.922427000	0.217385000	-0.252780000
H	3.124918000	-1.042243000	0.701891000
H	2.946352000	-1.020530000	-1.058205000

D

Y	0.243322000	-0.446044000	-0.095876000
C	2.687980000	0.706544000	-0.393053000
C	2.915352000	-0.711688000	-0.292160000
C	2.352231000	-1.353685000	-1.431861000
C	1.799023000	-0.344532000	-2.277011000
C	2.002413000	0.911781000	-1.647403000
C	3.826089000	-1.420556000	0.671700000
H	4.867689000	-1.332459000	0.334491000
H	3.789045000	-1.014196000	1.684610000
H	3.609244000	-2.492097000	0.722878000
C	2.505825000	-2.805574000	-1.791367000
H	3.500798000	-2.997991000	-2.215000000

H	2.398340000	-3.473700000	-0.928639000
H	1.775316000	-3.114749000	-2.544948000
C	1.291175000	-0.557732000	-3.673802000
H	2.126433000	-0.572697000	-4.387023000
H	0.762329000	-1.509968000	-3.782121000
H	0.618020000	0.243024000	-3.999000000
C	1.771117000	2.229857000	-2.335413000
H	2.608845000	2.455330000	-3.007915000
H	0.869710000	2.227285000	-2.959253000
H	1.689440000	3.064903000	-1.634529000
C	-1.974199000	-1.443578000	-2.451642000
O	-0.878756000	-1.932853000	-1.632340000
C	-1.129875000	-3.321832000	-1.255579000
C	-2.509452000	-3.658126000	-1.807982000
C	-2.644155000	-2.695750000	-2.991146000
H	-2.654973000	-0.863345000	-1.816658000
H	-1.545336000	-0.798010000	-3.221765000
H	-0.337103000	-3.925557000	-1.706881000
H	-1.082137000	-3.390940000	-0.164560000
H	-2.586151000	-4.709005000	-2.099550000
H	-3.278799000	-3.453928000	-1.056121000
H	-2.102790000	-3.071889000	-3.866327000
H	-3.682706000	-2.515197000	-3.281665000
Si	3.623525000	2.098219000	0.516466000
C	4.805883000	2.900185000	-0.721160000
H	4.288422000	3.417976000	-1.535066000
H	5.437687000	3.638551000	-0.212054000
H	5.469455000	2.151973000	-1.170496000
C	4.688308000	1.494178000	1.959220000
H	5.457182000	0.778798000	1.650985000
H	5.211012000	2.370164000	2.364776000
H	4.123383000	1.054190000	2.788365000
C	2.483093000	3.436716000	1.228166000
H	3.104517000	4.204433000	1.706544000
H	1.875449000	3.949155000	0.474140000
H	1.811981000	3.048120000	2.004578000
N	0.494904000	-1.134425000	2.328274000
C	-1.664706000	-1.056518000	1.250284000
C	-0.974606000	-1.247456000	2.452374000
C	-1.581809000	-1.558606000	3.674133000
H	-1.005924000	-1.690992000	4.587911000
C	-2.964882000	-1.711758000	3.701651000
H	-3.465158000	-1.955609000	4.635066000
C	-3.704865000	-1.563012000	2.523951000
H	-4.783943000	-1.698825000	2.546966000
C	-3.061018000	-1.238598000	1.330608000
H	-3.678568000	-1.127987000	0.437678000
C	1.131127000	-2.431304000	2.634618000
H	0.919851000	-2.743426000	3.666093000
H	2.211855000	-2.358046000	2.507002000
H	0.738259000	-3.201214000	1.963633000
C	1.047561000	-0.083378000	3.202080000
H	0.536032000	0.864492000	3.007813000
H	2.114829000	0.039516000	2.999978000
H	0.917706000	-0.325383000	4.265837000
C	-0.718717000	2.186457000	-0.163964000
H	-0.572733000	2.451968000	-1.208786000
H	0.125523000	2.363549000	0.503920000
C	-1.953752000	1.934150000	0.318841000
H	-2.082034000	1.775631000	1.389434000
C	-3.210691000	1.958452000	-0.486390000
C	-4.091540000	3.161683000	-0.093056000
H	-3.781287000	1.039229000	-0.301215000
H	-2.979826000	2.005142000	-1.558702000

C	-5.412627000	3.197265000	-0.864162000
H	-4.297865000	3.122892000	0.985640000
H	-3.533105000	4.090414000	-0.270472000
C	-6.286052000	4.389514000	-0.480608000
H	-5.203059000	3.226423000	-1.942777000
H	-5.962642000	2.263129000	-0.681878000
H	-7.224768000	4.387276000	-1.044460000
H	-6.538698000	4.370857000	0.586082000
H	-5.777315000	5.338624000	-0.686301000

TS2, Imaginary frequency = 230.2i

Y	0.159209000	-0.287771000	-0.092756000
C	2.712550000	0.362807000	-0.488192000
C	2.692104000	-1.081319000	-0.470077000
C	1.978264000	-1.544800000	-1.607454000
C	1.554836000	-0.406388000	-2.357507000
C	2.011491000	0.758449000	-1.685833000
C	3.479062000	-2.004138000	0.422406000
H	4.401004000	-2.322135000	-0.083548000
H	3.778752000	-1.533538000	1.358354000
H	2.930695000	-2.920806000	0.670558000
C	1.878343000	-2.974523000	-2.056683000
H	2.718586000	-3.230676000	-2.716515000
H	1.914377000	-3.678299000	-1.217953000
H	0.960921000	-3.170732000	-2.621563000
C	0.911407000	-0.440117000	-3.715265000
H	1.665684000	-0.321204000	-4.504878000
H	0.406583000	-1.392888000	-3.904484000
H	0.181406000	0.365712000	-3.859946000
C	1.944649000	2.135402000	-2.284425000
H	2.834187000	2.327069000	-2.899006000
H	1.078237000	2.253593000	-2.943945000
H	1.893118000	2.926404000	-1.533110000
C	-2.242419000	-1.677573000	-2.222836000
O	-1.239158000	-1.928296000	-1.204930000
C	-1.456840000	-3.244950000	-0.614616000
C	-2.685189000	-3.818727000	-1.316474000
C	-2.710151000	-3.058983000	-2.645913000
H	-3.057127000	-1.094215000	-1.777282000
H	-1.769425000	-1.097675000	-3.017949000
H	-0.554161000	-3.833328000	-0.803317000
H	-1.602728000	-3.119323000	0.462407000
H	-2.611840000	-4.901995000	-1.444048000
H	-3.589501000	-3.605251000	-0.737127000
H	-2.007545000	-3.498219000	-3.362821000
H	-3.701309000	-3.036764000	-3.107062000
Si	3.886926000	1.535354000	0.444596000
C	5.299837000	1.981118000	-0.727563000
H	4.949508000	2.520917000	-1.614073000
H	6.030438000	2.622446000	-0.219360000
H	5.826845000	1.083444000	-1.071273000
C	4.672685000	0.782333000	1.996432000
H	5.354124000	-0.044212000	1.770243000
H	5.273625000	1.566826000	2.474228000
H	3.952862000	0.437059000	2.747998000
C	3.055086000	3.144511000	1.013919000
H	3.768287000	3.698105000	1.638260000
H	2.784217000	3.806765000	0.185164000
H	2.157233000	2.983089000	1.621680000
N	0.311665000	-0.394781000	2.428313000
C	-1.883351000	-0.311339000	1.352750000
C	-1.087317000	-0.820638000	2.403009000
C	-1.581110000	-1.747529000	3.333130000
H	-0.955639000	-2.121507000	4.138290000

C	-2.895352000	-2.190801000	3.232615000
H	-3.285144000	-2.895505000	3.961923000
C	-3.702101000	-1.729920000	2.191306000
H	-4.725039000	-2.085976000	2.096478000
C	-3.194790000	-0.808658000	1.276924000
H	-3.851225000	-0.480901000	0.474765000
C	1.230346000	-1.397178000	2.994490000
H	1.092613000	-1.519380000	4.077286000
H	2.254945000	-1.068084000	2.819797000
H	1.081286000	-2.366405000	2.510852000
C	0.495397000	0.874647000	3.165245000
H	-0.135983000	1.659358000	2.749549000
H	1.540135000	1.185736000	3.089256000
H	0.236380000	0.745758000	4.225424000
C	-0.628128000	1.989411000	-0.270073000
H	-0.799955000	2.211101000	-1.324751000
H	0.227287000	2.512045000	0.161362000
C	-1.758363000	1.756037000	0.542962000
H	-1.693288000	2.053025000	1.587595000
C	-3.141061000	1.939562000	-0.032796000
C	-3.419972000	3.433521000	-0.283350000
H	-3.903760000	1.561431000	0.653033000
H	-3.242969000	1.392258000	-0.980569000
C	-4.839857000	3.683503000	-0.796414000
H	-3.266812000	3.991859000	0.651323000
H	-2.689987000	3.829782000	-1.000509000
C	-5.122556000	5.162593000	-1.050345000
H	-4.995994000	3.114704000	-1.724456000
H	-5.564438000	3.290023000	-0.069825000
H	-6.144181000	5.312483000	-1.415105000
H	-5.007272000	5.752092000	-0.133108000
H	-4.437516000	5.576019000	-1.800087000

E

Y	-0.474584000	-0.405066000	-0.150946000
C	1.595459000	-2.002058000	-0.025731000
C	0.445096000	-2.844011000	0.196604000
C	-0.315071000	-2.908243000	-1.004344000
C	0.350104000	-2.117454000	-1.991922000
C	1.518320000	-1.570171000	-1.402109000
C	0.142099000	-3.662805000	1.423143000
H	0.639702000	-4.640832000	1.370742000
H	0.482350000	-3.185569000	2.344752000
H	-0.929705000	-3.865952000	1.529469000
C	-1.504601000	-3.792681000	-1.252296000
H	-1.188303000	-4.752131000	-1.683021000
H	-2.047834000	-4.027776000	-0.330951000
H	-2.217699000	-3.351188000	-1.957314000
C	-0.058050000	-1.981204000	-3.431765000
H	0.541285000	-2.641606000	-4.072459000
H	-1.105735000	-2.259588000	-3.588149000
H	0.075187000	-0.959931000	-3.804483000
C	2.557870000	-0.790146000	-2.157449000
H	3.437646000	-1.414170000	-2.363332000
H	2.175018000	-0.450329000	-3.123882000
H	2.906097000	0.093321000	-1.614650000
C	-3.354246000	-0.123084000	-1.816463000
O	-2.808602000	-0.620018000	-0.552245000
C	-3.873809000	-1.211399000	0.248532000
C	-5.163741000	-0.928292000	-0.512764000
C	-4.684810000	-0.838006000	-1.964169000
H	-3.469999000	0.961947000	-1.728304000
H	-2.625394000	-0.346936000	-2.598478000
H	-3.667568000	-2.282842000	0.333576000

H	-3.848199000	-0.748911000	1.239367000
H	-5.909453000	-1.711814000	-0.354364000
H	-5.597136000	0.025001000	-0.191743000
H	-4.542187000	-1.837092000	-2.390636000
H	-5.375850000	-0.286776000	-2.607609000
Si	3.198727000	-1.953566000	0.991925000
C	4.368042000	-3.248363000	0.267648000
H	4.606612000	-3.043153000	-0.781970000
H	5.313500000	-3.272161000	0.823400000
H	3.927481000	-4.251024000	0.316047000
C	2.951342000	-2.379987000	2.822915000
H	2.658868000	-3.424125000	2.974416000
H	3.911908000	-2.240003000	3.335066000
H	2.221342000	-1.745655000	3.340250000
C	4.053675000	-0.262392000	0.930972000
H	4.845221000	-0.236881000	1.690637000
H	4.532502000	-0.074615000	-0.035196000
H	3.377847000	0.575931000	1.137857000
N	-0.350595000	0.669074000	2.182592000
C	-0.999670000	2.439378000	0.574231000
C	-1.294470000	1.692681000	1.743267000
C	-2.491588000	1.915084000	2.440894000
H	-2.704754000	1.365413000	3.351601000
C	-3.411317000	2.858459000	1.997359000
H	-4.324470000	3.027041000	2.561149000
C	-3.144967000	3.588875000	0.840723000
H	-3.853080000	4.331801000	0.484529000
C	-1.960315000	3.370807000	0.148056000
H	-1.760618000	3.946292000	-0.750907000
C	-0.921305000	-0.348731000	3.082064000
H	-1.190780000	0.060007000	4.065831000
H	-0.174281000	-1.130613000	3.236469000
H	-1.818133000	-0.800259000	2.643832000
C	0.827962000	1.265028000	2.857340000
H	1.244626000	2.075327000	2.259866000
H	1.591637000	0.497170000	2.999966000
H	0.535969000	1.672612000	3.834839000
C	-0.077489000	1.448091000	-1.537710000
H	-0.914882000	1.917740000	-2.072054000
H	0.778507000	1.408761000	-2.219670000
C	0.267077000	2.267903000	-0.274617000
H	1.001152000	1.681898000	0.319009000
C	0.978211000	3.626997000	-0.501210000
C	2.383906000	3.506809000	-1.087154000
H	1.031080000	4.168912000	0.455442000
H	0.363106000	4.240655000	-1.171805000
C	3.065983000	4.863511000	-1.281903000
H	3.006639000	2.882946000	-0.427321000
H	2.340935000	2.987466000	-2.053689000
C	4.468428000	4.743897000	-1.873596000
H	2.443374000	5.489119000	-1.936776000
H	3.116760000	5.388018000	-0.317364000
H	4.929507000	5.728656000	-2.004000000
H	5.125922000	4.153275000	-1.223956000
H	4.445106000	4.255790000	-2.855553000

F

Y	0.599058000	-0.657300000	-0.114948000
C	3.079553000	-0.353966000	-0.883138000
C	2.841119000	-1.776729000	-0.944704000
C	1.910045000	-2.041267000	-1.984990000
C	1.571041000	-0.796150000	-2.603201000
C	2.282983000	0.232074000	-1.936803000
C	3.552786000	-2.823298000	-0.127131000

H	4.635939000	-2.776946000	-0.291454000	H	6.192548000	-1.195245000	-1.345835000
H	3.397956000	-2.715678000	0.953972000	C	5.060149000	-0.169266000	1.600840000
H	3.238701000	-3.834045000	-0.403362000	H	5.068125000	-1.256558000	1.725520000
C	1.466778000	-3.380016000	-2.506462000	H	6.064190000	0.190065000	1.860478000
H	1.900062000	-3.564802000	-3.497987000	H	4.371914000	0.265518000	2.334974000
H	1.786324000	-4.206085000	-1.864960000	C	4.619121000	2.302779000	-0.084712000
H	0.378363000	-3.452213000	-2.624352000	H	5.522644000	2.643829000	0.437034000
C	0.752058000	-0.656494000	-3.852821000	H	4.626897000	2.764376000	-1.076890000
H	1.391580000	-0.750091000	-4.741128000	H	3.757181000	2.690608000	0.469264000
H	-0.017507000	-1.432324000	-3.930428000	N	-3.975192000	0.624196000	1.780799000
H	0.249095000	0.312694000	-3.916839000	C	-3.563232000	-0.004398000	-0.569329000
C	2.275012000	1.674468000	-2.363084000	C	-4.239411000	-0.221869000	0.658198000
H	3.144545000	1.898264000	-2.995642000	C	-5.160221000	-1.277851000	0.754975000
H	1.385531000	1.911387000	-2.954420000	H	-5.684354000	-1.449490000	1.689992000
H	2.306029000	2.363840000	-1.514958000	C	-5.450353000	-2.094270000	-0.335991000
C	-1.772407000	-3.044739000	-0.068209000	H	-6.181319000	-2.892306000	-0.232876000
O	-0.419440000	-2.736115000	0.431309000	C	-4.834651000	-1.849802000	-1.560364000
C	0.182904000	-3.946603000	0.978029000	H	-5.078245000	-2.451012000	-2.432572000
C	-0.970740000	-4.918220000	1.160462000	C	-3.906174000	-0.815515000	-1.658635000
C	-1.876629000	-4.559699000	-0.018597000	H	-3.424707000	-0.631291000	-2.616577000
H	-2.490461000	-2.550115000	0.590820000	C	-4.267715000	0.055500000	3.087476000
H	-1.866841000	-2.619957000	-1.067846000	H	-5.346393000	-0.026932000	3.307291000
H	0.919002000	-4.313705000	0.257619000	H	-3.830062000	0.701280000	3.857171000
H	0.691766000	-3.682042000	1.908111000	H	-3.824195000	-0.941359000	3.176348000
H	-0.633219000	-5.958115000	1.145527000	C	-4.582766000	1.952578000	1.670794000
H	-1.482509000	-4.738088000	2.112790000	H	-4.343901000	2.408538000	0.709305000
H	-1.498779000	-5.008293000	-0.944129000	H	-4.190034000	2.597605000	2.464797000
H	-2.911337000	-4.883376000	0.119176000	H	-5.680894000	1.912718000	1.767165000
Si	4.659302000	0.415754000	-0.157669000	C	-1.157656000	0.506574000	-1.252784000
C	6.070496000	-0.106842000	-1.303375000	H	-1.358204000	-0.103314000	-2.150877000
H	5.892886000	0.242744000	-2.327236000	H	-0.572076000	1.352680000	-1.661576000
H	7.023100000	0.320100000	-0.966380000	C	-2.511807000	1.079873000	-0.757493000

H	-2.369046000	1.553037000	0.223797000
C	-3.060534000	2.179270000	-1.708852000
C	-2.315703000	3.512803000	-1.638505000
H	-4.122705000	2.360627000	-1.488521000
H	-3.032368000	1.797320000	-2.739575000
C	-2.856161000	4.561883000	-2.613012000
H	-2.380195000	3.908524000	-0.612014000
H	-1.245584000	3.359781000	-1.842961000
C	-2.117714000	5.895836000	-2.530151000
H	-2.790519000	4.168698000	-3.637239000
H	-3.925745000	4.720894000	-2.417556000
H	-2.525600000	6.622739000	-3.240798000
H	-2.197528000	6.334006000	-1.527406000
H	-1.050778000	5.775195000	-2.757008000
N	0.619842000	-0.005476000	2.371540000
C	0.433008000	2.247388000	1.435836000
C	1.047039000	1.386206000	2.355840000
C	2.031535000	1.890094000	3.211125000
H	2.521536000	1.255124000	3.940404000
C	2.388948000	3.237252000	3.143626000
H	3.150812000	3.616179000	3.818977000
C	1.777916000	4.092138000	2.229289000
C	0.799176000	3.590737000	1.372651000
H	0.307631000	4.242789000	0.656586000
C	-0.785029000	-0.123304000	2.841126000
H	-0.873767000	0.240865000	3.873698000
H	-1.078180000	-1.177026000	2.812126000
H	-1.476354000	0.443264000	2.210668000
C	1.467624000	-0.881158000	3.197205000
H	1.110186000	-1.908934000	3.097457000
H	1.414506000	-0.615955000	4.262384000
H	2.507385000	-0.832411000	2.867453000
H	-0.365757000	1.888607000	0.787149000
H	2.059856000	5.139871000	2.186064000

TS3, Imaginary frequency = 1351.78i

Y	-0.857717000	0.162329000	0.103041000
C	-3.216393000	0.530397000	-0.923780000
C	-2.877859000	1.798271000	-0.315655000
C	-1.811125000	2.387875000	-1.044222000
C	-1.474294000	1.508406000	-2.120085000
C	-2.338293000	0.381624000	-2.058773000
C	-3.635518000	2.476006000	0.793307000
H	-4.565038000	2.919108000	0.412683000
H	-3.916034000	1.787815000	1.595581000
H	-3.063825000	3.294773000	1.241382000
C	-1.225856000	3.757869000	-0.844227000
H	-1.604233000	4.452946000	-1.605281000
H	-1.488584000	4.184514000	0.128839000
H	-0.132325000	3.766711000	-0.924953000
C	-0.509380000	1.829102000	-3.225359000
H	-1.032412000	2.314746000	-4.060318000
H	0.276397000	2.518035000	-2.899473000
H	-0.022551000	0.935825000	-3.627960000
C	-2.392104000	-0.690902000	-3.111033000
H	-3.218619000	-0.503302000	-3.809285000
H	-1.472510000	-0.714902000	-3.700814000
H	-2.536203000	-1.691201000	-2.695440000
C	-0.599899000	2.120125000	2.906282000
O	-1.081683000	0.824691000	2.433036000
C	-1.864625000	0.183016000	3.482668000
C	-1.619485000	1.009632000	4.736130000
C	-1.400965000	2.411830000	4.164166000
H	0.470614000	2.021801000	3.114459000

H	-0.749988000	2.846116000	2.103116000
H	-2.915722000	0.197673000	3.176837000
H	-1.525381000	-0.851512000	3.569274000
H	-2.461139000	0.955650000	5.431793000
H	-0.721387000	0.661176000	5.257869000
H	-2.357834000	2.882298000	3.913407000
H	-0.861662000	3.074167000	4.846641000
Si	-4.852688000	-0.387614000	-0.624785000
C	-6.253705000	0.695232000	-1.282885000
H	-6.115239000	0.910780000	-2.348738000
H	-7.219766000	0.188238000	-1.168507000
H	-6.321250000	1.654574000	-0.757997000
C	-5.174200000	-0.734535000	1.215576000
H	-5.361760000	0.174523000	1.797163000
H	-6.073197000	-1.357738000	1.303902000
H	-4.356466000	-1.287272000	1.694247000
C	-4.955548000	-2.058303000	-1.507782000
H	-5.898320000	-2.537335000	-1.214097000
H	-4.971626000	-1.955739000	-2.597348000
H	-4.147023000	-2.752028000	-1.250729000
N	4.323819000	-1.819031000	-0.582677000
C	3.382677000	-0.180082000	0.989005000
C	3.999442000	-1.433995000	0.746345000
C	4.262202000	-2.283120000	1.833649000
C	3.965553000	-1.907423000	3.141409000
H	4.197224000	-2.581843000	3.961884000
C	3.412821000	-0.653934000	3.391130000
H	3.214246000	-0.330127000	4.409692000
C	3.128615000	0.186784000	2.316304000
H	2.700583000	1.167929000	2.514142000
C	4.350814000	-3.246268000	-0.852727000
H	4.359752000	-3.395726000	-1.937753000
H	3.450871000	-3.720981000	-0.452243000
C	5.509579000	-1.163145000	-1.127769000
H	5.467231000	-0.085410000	-0.959327000
H	5.557182000	-1.339650000	-2.208151000
C	1.479705000	1.070003000	-0.134162000
H	1.242505000	1.578392000	0.822788000
H	1.269406000	1.805405000	-0.923130000
C	2.996028000	0.762633000	-0.139968000
H	3.215060000	0.237578000	-1.078003000
C	3.853608000	2.050258000	-0.103246000
C	3.814884000	2.888577000	-1.381752000
H	4.897203000	1.773706000	0.103015000
H	3.539738000	2.667919000	0.751688000
C	4.723525000	4.118818000	-1.322105000
H	4.111778000	2.261039000	-2.236007000
H	2.786824000	3.219486000	-1.589508000
C	4.688522000	4.951712000	-2.601421000
H	4.429763000	4.744517000	-0.467192000
H	5.754901000	3.796890000	-1.121424000
H	5.346361000	5.824269000	-2.526742000
H	5.014867000	4.363726000	-3.467833000
H	3.675571000	5.317176000	-2.811510000
N	-0.645210000	-2.271148000	0.805641000
C	0.516884000	-1.464658000	-1.162484000
C	-0.135098000	-2.542459000	-0.539765000
C	-0.290847000	-3.778924000	-1.173606000
C	0.245940000	-3.955144000	-2.448144000
H	0.136640000	-4.914954000	-2.945914000
C	0.925412000	-2.912082000	-3.079625000
C	1.051533000	-1.680679000	-2.439783000
H	1.576434000	-0.871457000	-2.943504000
C	0.434720000	-2.452291000	1.804929000

H	0.071855000	-2.158324000	2.793977000
H	1.303721000	-1.843005000	1.547604000
C	-1.809130000	-3.083718000	1.189632000
H	-2.208777000	-2.713507000	2.137987000
H	-2.587008000	-3.012629000	0.426716000
H	1.035056000	-0.269770000	-0.596533000
H	1.347961000	-3.062380000	-4.069667000
H	4.729810000	-3.246664000	1.655801000
H	-0.813122000	-4.602554000	-0.695746000
H	-1.544945000	-4.140692000	1.331621000
H	0.748498000	-3.504481000	1.844478000
H	5.236697000	-3.762436000	-0.441182000
H	6.442144000	-1.541432000	-0.673959000

Product 7b

N	1.625826000	-1.671835000	0.210882000
C	0.826474000	0.649292000	0.182034000
C	1.858488000	-0.288343000	-0.053780000
C	3.100066000	0.160650000	-0.525297000
H	3.894842000	-0.558179000	-0.704190000
C	3.333758000	1.509884000	-0.777504000
H	4.306690000	1.831638000	-1.141250000
C	2.313920000	2.434695000	-0.574170000
H	2.477792000	3.490802000	-0.773003000
C	1.080380000	1.996262000	-0.098274000
H	0.294293000	2.726940000	0.080896000
C	2.772469000	-2.440991000	0.653317000
H	3.491329000	-2.685913000	-0.151367000
H	2.419484000	-3.390480000	1.073754000
H	3.304634000	-1.895579000	1.438092000
C	0.893442000	-2.361875000	-0.841572000
H	0.004118000	-1.791966000	-1.122268000
H	0.570067000	-3.344953000	-0.478360000
H	1.501328000	-2.513777000	-1.752833000
C	-0.731563000	0.908296000	2.136575000
H	-0.823109000	1.997306000	2.041134000
H	-1.642485000	0.537475000	2.619454000
C	-0.512858000	0.237371000	0.771678000
H	-0.465593000	-0.843265000	0.948815000
C	-1.670174000	0.514761000	-0.209740000
C	-3.018320000	-0.080455000	0.202049000
H	-1.396670000	0.119742000	-1.198927000
H	-1.779535000	1.600942000	-0.346495000
C	-4.111504000	0.130668000	-0.847760000
H	-2.899028000	-1.158731000	0.388531000
H	-3.351202000	0.357313000	1.153277000
C	-5.459741000	-0.458779000	-0.438821000
H	-4.224989000	1.207168000	-1.040304000
H	-3.790024000	-0.314295000	-1.800411000
H	-6.220676000	-0.294113000	-1.210413000
H	-5.385177000	-1.540379000	-0.270757000
H	-5.825513000	-0.005623000	0.490955000
H	0.109623000	0.702166000	2.807085000

THF

C	1.158653000	-0.429709000	-0.133151000
O	-0.000431000	-1.246435000	0.000302000
C	-1.159052000	-0.428917000	0.132771000
C	-0.729561000	0.993145000	-0.229119000
C	0.730384000	0.992524000	0.229283000
H	1.945397000	-0.823979000	0.521273000
H	1.525498000	-0.478233000	-1.170797000
H	-1.945580000	-0.822460000	-0.522377000
H	-1.526786000	-0.477378000	1.170093000

H	-0.787587000	1.146859000	-1.313645000
H	-1.341813000	1.759785000	0.256193000
H	1.343230000	1.758886000	-0.255737000
H	0.788544000	1.145739000	1.313874000

A’

Y	-0.309437000	-0.387181000	-0.295552000
C	-1.926115000	-0.989441000	1.255062000
H	-1.810149000	-2.038134000	1.576135000
H	-2.085419000	-0.384313000	2.153870000
C	1.912500000	0.603679000	0.191583000
C	1.414551000	1.391762000	-0.906330000
C	0.313058000	2.165267000	-0.444462000
C	0.134363000	1.893136000	0.953430000
C	1.113955000	0.943662000	1.346847000
C	1.950279000	1.415849000	-2.314171000
H	3.022066000	1.638443000	-2.317120000
H	1.828063000	0.466563000	-2.857373000
H	1.457763000	2.188118000	-2.910535000
C	-0.427653000	3.221632000	-1.217411000
H	0.049116000	4.200995000	-1.081233000
H	-0.447813000	3.021640000	-2.293353000
H	-1.464717000	3.326649000	-0.882309000
C	-0.818853000	2.618343000	1.856799000
H	-0.340058000	3.513078000	2.276095000
H	-1.712357000	2.957169000	1.323470000
H	-1.150306000	2.001419000	2.696704000
C	1.295729000	0.429920000	2.749336000
H	2.280125000	0.711844000	3.139975000
H	0.543829000	0.850447000	3.421103000
H	1.215558000	-0.660885000	2.827070000
Si	-3.341465000	-0.837603000	0.039283000
C	-4.507420000	0.592314000	0.402746000
H	-5.069273000	0.389617000	1.322093000
H	-3.969001000	1.535572000	0.551582000
H	-5.234333000	0.740963000	-0.404068000
C	-4.245932000	-2.451084000	-0.309520000
H	-4.967693000	-2.348358000	-1.128190000
H	-3.555661000	-3.263330000	-0.568379000
H	-4.798313000	-2.766853000	0.583330000
C	-2.537729000	-0.382234000	-1.719352000
H	-2.028799000	0.595390000	-1.854986000
H	-1.958122000	-1.175233000	-2.232604000
H	-3.415836000	-0.267472000	-2.366924000
Si	2.975774000	-0.945356000	0.032413000
C	3.873473000	-1.441692000	1.606183000
H	3.211696000	-1.736738000	2.425225000
H	4.535311000	-2.289745000	1.392389000
H	4.503474000	-0.619044000	1.964051000
C	4.202341000	-0.911127000	-1.392105000
H	4.891556000	-0.064903000	-1.288986000
H	4.806657000	-1.826152000	-1.365297000
H	3.741516000	-0.849881000	-2.382175000
C	1.697280000	-2.362656000	-0.364416000
H	2.286688000	-3.286230000	-0.433908000
H	0.959293000	-2.601412000	0.425182000
H	1.199920000	-2.321726000	-1.353389000

B’

Y	0.143990000	-0.071319000	-0.108881000	H	1.621004000	5.947942000	0.047683000
C	0.216666000	2.191739000	-0.697632000	C	3.081696000	3.296919000	-1.179492000
H	0.228367000	2.212010000	-1.806390000	H	3.800321000	4.073461000	-0.889803000
H	-0.764399000	2.610196000	-0.416698000	H	3.605867000	2.337151000	-1.074433000
C	-2.294808000	-0.392454000	0.568638000	H	2.863866000	3.443900000	-2.244963000
C	-1.713011000	-1.711857000	0.683632000	C	1.977693000	3.152065000	1.689437000
C	-0.793408000	-1.713503000	1.765543000	H	1.109076000	3.266715000	2.349593000
C	-0.789261000	-0.403365000	2.341375000	H	2.399053000	2.152073000	1.865811000
C	-1.731622000	0.397481000	1.633320000	H	2.730404000	3.878210000	2.020433000
C	-2.105679000	-2.937724000	-0.097926000	Si	-3.627707000	0.070692000	-0.708780000
H	-2.952516000	-3.443451000	0.383889000	C	-4.283266000	1.828323000	-0.523455000
H	-2.417255000	-2.710516000	-1.121986000	H	-3.528122000	2.595913000	-0.725477000
H	-1.294539000	-3.673283000	-0.148246000	H	-5.088235000	1.968764000	-1.256185000
C	-0.053370000	-2.900783000	2.312304000	H	-4.711030000	2.016624000	0.466735000
H	0.948773000	-2.639455000	2.669811000	C	-5.087503000	-1.116287000	-0.572915000
H	-0.594339000	-3.330614000	3.165843000	H	-5.519921000	-1.085477000	0.434131000
H	0.058808000	-3.698254000	1.571298000	H	-5.874238000	-0.820631000	-1.277892000
C	-0.050385000	-0.008665000	3.590205000	H	-4.825006000	-2.156270000	-0.790566000
H	0.255911000	1.042261000	3.584170000	C	-2.849064000	-0.051502000	-2.443048000
H	-0.686481000	-0.150656000	4.474074000	H	-3.594395000	0.204899000	-3.206699000
H	0.847745000	-0.615799000	3.745332000	H	-2.024734000	0.666436000	-2.567193000
C	-2.130016000	1.782582000	2.057488000	H	-2.483289000	-1.056684000	-2.688061000
H	-2.997560000	1.737989000	2.729532000	N	1.374369000	-1.252232000	-2.023042000
H	-1.327230000	2.282215000	2.607857000	C	2.894475000	-0.685288000	-0.190362000
H	-2.402914000	2.422598000	1.217422000	C	2.290515000	-1.678768000	-0.994642000
Si	1.506025000	3.447326000	-0.127436000	C	2.515573000	-3.025273000	-0.680750000
C	0.871815000	5.220353000	-0.287886000	H	2.080431000	-3.815740000	-1.281198000
H	0.620202000	5.457809000	-1.328521000	C	3.333169000	-3.365079000	0.397301000
H	-0.032375000	5.376625000	0.313208000	H	3.510053000	-4.415242000	0.611692000

C	3.926475000	-2.383708000	1.190392000
H	4.563602000	-2.662869000	2.023769000
C	3.707607000	-1.041830000	0.889938000
H	4.182253000	-0.260474000	1.476617000
C	0.680976000	-2.337300000	-2.738281000
H	1.379588000	-2.946056000	-3.328030000
H	-0.045567000	-1.889729000	-3.419923000
H	0.145710000	-2.982074000	-2.037402000
C	1.970566000	-0.314050000	-3.009425000
H	2.415216000	0.551949000	-2.518779000
H	1.179619000	0.041737000	-3.673756000
H	2.740351000	-0.822696000	-3.603711000
H	2.830288000	0.371157000	-0.460887000

TS1', Imaginary frequency = 1384.9i

Y	0.277075000	0.182265000	-0.238288000
C	-1.238429000	-1.727483000	-0.399758000
H	-0.918623000	-1.860807000	-1.455457000
H	-0.525252000	-2.292090000	0.230191000
C	2.594738000	-0.210092000	0.699025000
C	2.328066000	1.196464000	0.899729000
C	1.324796000	1.330452000	1.898057000
C	0.963998000	0.015814000	2.338107000
C	1.754766000	-0.925475000	1.623615000
C	3.030092000	2.347399000	0.226869000
H	4.031651000	2.491595000	0.650983000
H	3.167112000	2.204343000	-0.852037000
H	2.489296000	3.288420000	0.367321000
C	0.822817000	2.608100000	2.508271000
H	-0.244194000	2.558345000	2.747779000
H	1.356156000	2.822155000	3.443867000
H	0.970808000	3.471346000	1.852255000
C	0.040438000	-0.284176000	3.483766000
H	-0.484224000	-1.237116000	3.362291000
H	0.602059000	-0.343105000	4.425623000
H	-0.715682000	0.496456000	3.610209000
C	1.763707000	-2.403093000	1.905304000
H	2.629384000	-2.667582000	2.525882000
H	0.871390000	-2.709640000	2.459122000
H	1.820959000	-3.018364000	1.003120000
Si	-2.877999000	-2.693275000	-0.254591000
C	-2.610109000	-4.447910000	-0.896534000
H	-2.299427000	-4.452571000	-1.948472000
H	-1.841061000	-4.975480000	-0.319349000
H	-3.533411000	-5.035962000	-0.826414000
C	-4.199894000	-1.829231000	-1.291086000
H	-5.159814000	-2.351177000	-1.196630000
H	-4.360466000	-0.791635000	-0.975013000
H	-3.942816000	-1.826289000	-2.357989000
C	-3.398408000	-2.757560000	1.558845000
H	-2.629369000	-3.228240000	2.183317000
H	-3.605523000	-1.763032000	1.969790000
H	-4.313902000	-3.350927000	1.670851000
Si	3.624621000	-0.891312000	-0.732136000
C	3.967649000	-2.741279000	-0.644502000
H	3.076174000	-3.361875000	-0.783067000
H	4.671119000	-3.003643000	-1.444484000
H	4.434670000	-3.025930000	0.304621000

C	5.266033000	0.003773000	-0.953140000
H	5.867495000	-0.075340000	-0.039690000
H	5.836384000	-0.467832000	-1.762753000
H	5.166290000	1.065791000	-1.193972000
C	2.523972000	-0.594865000	-2.283942000
H	3.070646000	-0.864158000	-3.196978000
H	1.622659000	-1.233662000	-2.311887000
H	2.234296000	0.459563000	-2.428186000
N	-0.851025000	1.696821000	-1.883943000
C	-1.967460000	1.038721000	0.172972000
C	-1.774322000	2.042804000	-0.797861000
C	-2.403352000	3.287087000	-0.709012000
H	-2.241884000	4.060077000	-1.454641000
C	-3.266016000	3.531145000	0.358515000
H	-3.768828000	4.491622000	0.429966000
C	-3.489652000	2.550819000	1.327724000
H	-4.166814000	2.751200000	2.153616000
C	-2.840284000	1.321517000	1.233486000
H	-3.015476000	0.567696000	1.998090000
C	-0.106959000	2.837938000	-2.445403000
H	-0.755072000	3.508645000	-3.024745000
H	0.667742000	2.461190000	-3.120470000
H	0.363560000	3.411853000	-1.642637000
C	-1.553373000	0.974806000	-2.972114000
H	-2.113074000	0.132062000	-2.561630000
H	-0.821825000	0.607146000	-3.699458000
H	-2.258486000	1.641918000	-3.486259000
H	-1.732946000	-0.350878000	-0.057251000

C’

Y	0.099279000	-0.141548000	-0.443331000
C	-2.192126000	-0.087057000	0.576733000
C	-1.917273000	-1.486770000	0.384864000
C	-0.880307000	-1.873529000	1.280230000
C	-0.513505000	-0.723382000	2.054860000
C	-1.319727000	0.368228000	1.631155000
C	-2.595161000	-2.412363000	-0.591553000
H	-3.670572000	-2.471295000	-0.390987000
H	-2.493532000	-2.100983000	-1.641799000
H	-2.197676000	-3.428205000	-0.517648000
C	-0.382356000	-3.272220000	1.518948000
H	0.635531000	-3.282786000	1.920955000
H	-1.017245000	-3.784487000	2.253501000
H	-0.385362000	-3.885400000	0.611742000
C	0.450746000	-0.725120000	3.205242000
H	0.947828000	0.240508000	3.329699000
H	-0.070698000	-0.956348000	4.143481000
H	1.234911000	-1.478677000	3.081448000
C	-1.282737000	1.744301000	2.240450000
H	-2.256808000	2.003736000	2.670248000
H	-0.546598000	1.794359000	3.046269000
H	-1.028067000	2.535716000	1.524807000
Si	-3.072706000	1.017538000	-0.669316000
C	-3.620735000	2.682456000	0.010706000
H	-2.794049000	3.342418000	0.289266000
H	-4.217049000	3.202248000	-0.749086000
H	-4.258447000	2.553391000	0.892747000
C	-4.527002000	0.203842000	-1.540394000

H	-5.265838000	-0.142338000	-0.808034000
H	-5.024543000	0.943400000	-2.179514000
H	-4.256287000	-0.648007000	-2.170554000
C	-1.721144000	1.379871000	-2.023785000
H	-2.197765000	2.015401000	-2.781521000
H	-0.851805000	1.988390000	-1.708067000
H	-1.381156000	0.506382000	-2.613454000
N	2.281010000	-0.982908000	-1.217610000
C	1.985488000	1.048488000	0.133384000
C	2.883478000	0.197121000	-0.538568000
C	4.264516000	0.413475000	-0.574235000
H	4.937325000	-0.262722000	-1.096729000
C	4.777044000	1.528188000	0.082838000
H	5.846942000	1.717310000	0.067282000
C	3.920867000	2.402063000	0.761705000
H	4.332382000	3.269470000	1.271596000
C	2.549988000	2.159808000	0.786126000
H	1.907126000	2.853247000	1.325991000
C	2.798405000	-2.258346000	-0.679161000
H	3.863584000	-2.391621000	-0.908114000
H	2.243948000	-3.094566000	-1.117626000
H	2.675537000	-2.273571000	0.406539000
C	2.472108000	-0.932079000	-2.682669000
H	2.090314000	0.015756000	-3.077999000
H	1.940619000	-1.765525000	-3.155495000
H	3.532996000	-1.001837000	-2.956620000

D’

Y	0.225456000	0.315698000	-0.134554000
C	2.484247000	0.453211000	1.106297000
C	1.511415000	0.612800000	2.134560000
C	0.800851000	-0.619376000	2.259933000
C	1.348246000	-1.545371000	1.322944000
C	2.391445000	-0.888965000	0.580253000
N	0.017776000	2.463983000	-1.352779000
C	-1.444259000	1.796522000	0.492997000
C	-1.222258000	2.695290000	-0.567521000
C	-2.068958000	3.769348000	-0.859902000
H	-1.864414000	4.454107000	-1.679810000
C	-3.192003000	3.962636000	-0.060542000
H	-3.864035000	4.791406000	-0.266390000
C	-3.449708000	3.100248000	1.010848000
H	-4.325235000	3.265072000	1.634159000
C	-2.586979000	2.039621000	1.278779000
H	-2.811281000	1.387735000	2.122402000
C	1.015949000	3.521879000	-1.087030000
H	0.674402000	4.493767000	-1.467999000
H	1.963216000	3.268242000	-1.573620000
H	1.174237000	3.611327000	-0.010136000
C	-0.228325000	2.364679000	-2.805186000
H	-0.998731000	1.613342000	-3.005498000
H	0.697606000	2.079749000	-3.316126000
H	-0.570658000	3.317676000	-3.229429000
C	-0.930111000	-1.605657000	-1.854762000
H	-0.433988000	-2.472010000	-1.416045000
H	-0.488572000	-1.195823000	-2.764516000
C	-2.146303000	-1.203180000	-1.426828000
H	-2.641789000	-0.388237000	-1.959612000

C	-2.932198000	-1.814261000	-0.313227000
C	-4.227640000	-2.467838000	-0.838996000
H	-3.209262000	-1.029622000	0.405575000
H	-2.329453000	-2.561431000	0.218722000
C	-5.078450000	-3.056568000	0.288049000
H	-4.814370000	-1.719710000	-1.389973000
H	-3.966718000	-3.254343000	-1.559329000
C	-6.359522000	-3.710997000	-0.224053000
H	-4.484193000	-3.795473000	0.843608000
H	-5.330214000	-2.261236000	1.003642000
H	-6.947420000	-4.121156000	0.603526000
H	-6.991256000	-2.989192000	-0.754968000
H	-6.138839000	-4.533558000	-0.914262000
C	3.542468000	1.473722000	0.778776000
H	4.428256000	1.316645000	1.407721000
H	3.878621000	1.424158000	-0.261355000
H	3.202728000	2.496589000	0.969089000
C	1.353751000	1.801473000	3.037746000
H	0.315332000	1.945446000	3.349197000
H	1.956138000	1.672606000	3.947114000
H	1.685682000	2.731337000	2.565534000
C	-0.233591000	-0.918167000	3.307875000
H	-0.965521000	-1.663322000	2.978398000
H	0.240070000	-1.318093000	4.214466000
H	-0.784706000	-0.020220000	3.603139000
C	0.959598000	-2.999103000	1.267191000
H	1.017867000	-3.426836000	0.263321000
H	1.627508000	-3.595527000	1.902678000
H	-0.055019000	-3.164001000	1.644884000
Si	3.535481000	-1.565269000	-0.775822000
C	3.233707000	-3.382325000	-1.189711000
H	2.252187000	-3.573908000	-1.638524000
H	3.985388000	-3.691557000	-1.927071000
H	3.346774000	-4.037915000	-0.320010000
C	3.209770000	-0.581167000	-2.375673000
H	3.886884000	-0.918759000	-3.170554000
H	2.190573000	-0.742534000	-2.758843000
H	3.369244000	0.498348000	-2.263877000
C	5.339162000	-1.378390000	-0.260478000
H	5.639917000	-0.336988000	-0.110392000
H	5.531456000	-1.917990000	0.674309000
H	5.995807000	-1.804844000	-1.028632000

TS2', Imaginary frequency = 246.2i

Y	0.406289000	0.298094000	-0.266013000
C	2.455517000	0.370859000	1.270802000
C	1.382214000	0.562985000	2.182977000
C	0.626836000	-0.649700000	2.235143000
C	1.242333000	-1.596930000	1.367498000
C	2.374113000	-0.970561000	0.738458000
N	-0.189703000	2.192681000	-1.773695000
C	-1.704428000	1.399679000	-0.002530000
C	-1.162238000	2.499057000	-0.720605000
C	-1.470636000	3.822143000	-0.379809000
H	-1.064127000	4.653565000	-0.948554000
C	-2.314580000	4.077611000	0.696325000
H	-2.562275000	5.103458000	0.954059000
C	-2.827510000	3.018935000	1.447490000

H	-3.460189000	3.218997000	2.308124000
C	-2.527943000	1.705952000	1.092677000
H	-2.938239000	0.901331000	1.696144000
C	0.889426000	3.190089000	-1.910188000
H	0.536831000	4.129764000	-2.355108000
H	1.664055000	2.783467000	-2.567901000
H	1.326852000	3.415029000	-0.932485000
C	-0.810928000	1.971721000	-3.098115000
H	-1.599334000	1.222564000	-3.034531000
H	-0.047841000	1.622194000	-3.800186000
H	-1.246281000	2.904135000	-3.482069000
C	-1.072203000	-1.268045000	-1.328839000
H	-0.984629000	-2.250737000	-0.865571000
H	-0.769780000	-1.241318000	-2.380616000
C	-2.199737000	-0.479931000	-0.957072000
H	-2.646747000	0.137814000	-1.734205000
C	-3.229806000	-1.065712000	-0.020034000
C	-4.031919000	-2.169471000	-0.734036000
H	-3.933077000	-0.298186000	0.314824000
H	-2.746870000	-1.486500000	0.871808000
C	-5.140225000	-2.748835000	0.148280000
H	-4.475527000	-1.758637000	-1.652574000
H	-3.354131000	-2.971756000	-1.052337000
C	-5.940433000	-3.843132000	-0.554644000
H	-4.696916000	-3.151531000	1.070194000
H	-5.817151000	-1.941373000	0.460628000
H	-6.723781000	-4.240269000	0.099361000
H	-6.426001000	-3.461098000	-1.460424000
H	-5.297124000	-4.680761000	-0.849390000
C	3.533097000	1.388277000	0.995837000
H	4.416175000	1.194818000	1.618496000
H	3.879680000	1.385027000	-0.043830000
H	3.203248000	2.406337000	1.229703000
C	1.144907000	1.766739000	3.050322000
H	0.078284000	1.955778000	3.210914000
H	1.599615000	1.625190000	4.039889000
H	1.580679000	2.678577000	2.629066000
C	-0.488279000	-0.922453000	3.203257000
H	-1.179782000	-1.690019000	2.841657000
H	-0.087550000	-1.282365000	4.160460000
H	-1.071561000	-0.021906000	3.420327000
C	0.832004000	-3.041178000	1.281020000
H	0.995102000	-3.474504000	0.292379000
H	1.406404000	-3.644063000	1.996749000
H	-0.224990000	-3.177230000	1.531746000
Si	3.439092000	-1.581376000	-0.697651000
C	3.189621000	-3.391180000	-1.159752000
H	2.194086000	-3.605559000	-1.563070000
H	3.916948000	-3.653136000	-1.938557000
H	3.366969000	-4.061819000	-0.312087000
C	2.853677000	-0.539023000	-2.200801000
H	3.448082000	-0.774497000	-3.092924000
H	1.813261000	-0.766491000	-2.494867000
H	2.962226000	0.545698000	-2.049463000
C	5.278515000	-1.303193000	-0.402513000
H	5.550918000	-0.255348000	-0.247101000
H	5.606223000	-1.864478000	0.480799000
H	5.855263000	-1.673529000	-1.258909000

E’

Y	0.417211000	0.117992000	-0.115893000
C	2.936928000	0.562533000	-0.158398000
C	2.461382000	1.583651000	0.706316000
C	1.949298000	0.958129000	1.890121000
C	2.123353000	-0.444971000	1.766492000
C	2.705808000	-0.716352000	0.475513000
N	-0.878930000	1.284092000	-2.011883000
C	-2.118530000	1.300280000	0.136400000
C	-1.484916000	2.024452000	-0.913380000
C	-1.394066000	3.424598000	-0.848224000
H	-0.928007000	3.978592000	-1.655724000
C	-1.915664000	4.122738000	0.233966000
H	-1.846477000	5.206363000	0.258138000
C	-2.526340000	3.427549000	1.276996000
H	-2.933407000	3.963326000	2.129524000
C	-2.614313000	2.042539000	1.222168000
H	-3.088034000	1.508711000	2.040560000
C	0.128314000	2.031347000	-2.787876000
H	-0.317848000	2.831174000	-3.394944000
H	0.623814000	1.333821000	-3.469537000
H	0.880848000	2.474490000	-2.128163000
C	-1.880694000	0.739857000	-2.960335000
H	-2.700303000	0.259418000	-2.427597000
H	-1.400471000	0.007051000	-3.615644000
H	-2.297192000	1.549225000	-3.574602000
C	-1.253488000	-0.801519000	1.208596000
H	-1.438860000	-0.403051000	2.212963000
H	-1.324279000	-1.896447000	1.259222000
C	-2.261003000	-0.231275000	0.183608000
H	-1.980306000	-0.629747000	-0.813457000
C	-3.742191000	-0.653720000	0.350768000
C	-3.988924000	-2.146401000	0.133948000
H	-4.363003000	-0.076912000	-0.351372000
H	-4.074920000	-0.375998000	1.358941000
C	-5.462079000	-2.530764000	0.290158000
H	-3.645630000	-2.434734000	-0.872575000
H	-3.388333000	-2.729070000	0.844979000
C	-5.714880000	-4.021817000	0.079459000
H	-5.805193000	-2.238780000	1.292521000
H	-6.067055000	-1.950248000	-0.420434000
H	-6.775562000	-4.264393000	0.202910000
H	-5.417769000	-4.339248000	-0.927581000
H	-5.150574000	-4.626678000	0.799474000
C	3.626962000	0.796628000	-1.476636000
H	4.694726000	0.557410000	-1.403066000
H	3.226890000	0.185802000	-2.296134000
H	3.557455000	1.843161000	-1.787921000
C	2.606129000	3.069285000	0.526185000
H	1.727846000	3.619007000	0.883326000
H	3.466349000	3.442785000	1.097347000
H	2.774031000	3.352629000	-0.517665000
C	1.461336000	1.692953000	3.104780000
H	0.780311000	1.084498000	3.706591000
H	2.302479000	1.975105000	3.752311000
H	0.936171000	2.617965000	2.843147000
C	1.840140000	-1.428133000	2.865650000

H	1.507109000	-2.398348000	2.494410000
H	2.745673000	-1.600314000	3.462777000
H	1.068235000	-1.057924000	3.546028000
Si	2.770400000	-2.357806000	-0.450652000
C	2.608637000	-3.898454000	0.619024000
H	1.614124000	-4.023550000	1.059283000
H	2.802606000	-4.780669000	-0.003668000
H	3.343127000	-3.904992000	1.431756000
C	1.203474000	-2.310845000	-1.587016000
H	1.213988000	-3.192515000	-2.240846000
H	0.243605000	-2.403734000	-1.045585000
H	1.160947000	-1.457825000	-2.288902000
C	4.291397000	-2.551215000	-1.544008000
H	4.353404000	-1.821649000	-2.356122000
H	5.204608000	-2.459838000	-0.943908000
H	4.294127000	-3.551354000	-1.994299000

F'

Y	-0.383103000	0.151030000	0.326836000
C	-2.977672000	-0.296447000	0.598661000
C	-2.326476000	-1.581647000	0.649160000
C	-1.573136000	-1.671247000	1.853346000
C	-1.737051000	-0.450913000	2.569554000
C	-2.606515000	0.386323000	1.817476000
N	0.479388000	-0.331980000	-2.040235000
C	1.660412000	-2.168294000	-0.829178000
C	0.744333000	-1.760251000	-1.834991000
C	0.106763000	-2.711040000	-2.642211000
H	-0.566115000	-2.396561000	-3.432605000
C	0.324456000	-4.072210000	-2.458990000
H	-0.178593000	-4.790699000	-3.099589000
C	1.189611000	-4.493821000	-1.455115000
H	1.370033000	-5.552557000	-1.291671000
C	1.840279000	-3.550077000	-0.665707000
H	2.527011000	-3.897646000	0.098510000
C	-0.780349000	-0.039454000	-2.750602000
H	-0.756680000	-0.348000000	-3.804893000
H	-0.941706000	1.044554000	-2.739211000
H	-1.629878000	-0.537695000	-2.270044000
C	1.574858000	0.313435000	-2.804977000
H	2.541367000	0.088339000	-2.358847000
H	1.431742000	1.396845000	-2.811324000
H	1.573999000	-0.061210000	-3.837931000
C	1.559120000	-0.776622000	1.277931000
H	1.404349000	-1.649162000	1.924438000
H	2.116303000	-0.046112000	1.889904000
C	2.432751000	-1.197362000	0.071556000
H	2.642125000	-0.293589000	-0.516657000
N	0.127563000	2.691715000	0.598344000
C	2.504120000	2.539965000	-0.073026000
C	1.210435000	3.025513000	-0.318479000
C	0.998337000	3.823305000	-1.452192000
H	0.022953000	4.237482000	-1.676456000
C	2.058241000	4.133463000	-2.306691000
H	1.870719000	4.766615000	-3.169531000
C	3.341520000	3.660780000	-2.049678000
H	4.165766000	3.918526000	-2.707905000
C	3.555168000	2.860811000	-0.928586000

H	4.550078000	2.486598000	-0.705011000
C	-1.102196000	3.461888000	0.329469000
H	-0.912947000	4.543802000	0.362043000
H	-1.842282000	3.214219000	1.087683000
H	-1.518523000	3.204950000	-0.647391000
C	0.517027000	2.917589000	2.014836000
H	1.360334000	2.287803000	2.296540000
H	-0.328321000	2.658520000	2.656162000
H	0.786604000	3.969023000	2.180222000
H	2.709871000	1.917371000	0.790982000
C	3.820675000	-1.754007000	0.469173000
C	4.776088000	-0.698559000	1.025717000
H	4.291861000	-2.226802000	-0.405472000
H	3.695193000	-2.542231000	1.224228000
C	6.137569000	-1.272454000	1.425149000
H	4.927836000	0.089414000	0.271208000
H	4.326099000	-0.210315000	1.901820000
C	7.095782000	-0.216939000	1.972138000
H	5.989168000	-2.058547000	2.178559000
H	6.591965000	-1.766710000	0.555128000
H	8.057653000	-0.659743000	2.251583000
H	7.295831000	0.564173000	1.228055000
H	6.683956000	0.270523000	2.864411000
C	-2.527279000	-2.754150000	-0.272143000
H	-2.708575000	-2.463339000	-1.308858000
H	-1.663851000	-3.425882000	-0.265672000
H	-3.395463000	-3.345869000	0.049887000
C	-0.903078000	-2.908972000	2.373575000
H	-0.331147000	-3.435720000	1.602592000
H	-0.221021000	-2.684823000	3.198530000
H	-1.655109000	-3.612099000	2.756632000
C	-1.207559000	-0.180707000	3.949534000
H	-1.249644000	0.882048000	4.209037000
H	-1.801762000	-0.713027000	4.704368000
H	-0.169336000	-0.511087000	4.067158000
C	-3.269405000	1.613912000	2.383638000
H	-4.085314000	1.317078000	3.055890000
H	-2.593087000	2.231546000	2.986442000
H	-3.713307000	2.251056000	1.614126000
Si	-4.449448000	0.218665000	-0.489734000
C	-6.006593000	0.212270000	0.579599000
H	-5.964027000	0.937553000	1.398830000
H	-6.886168000	0.454299000	-0.029603000
H	-6.172003000	-0.777207000	1.021724000
C	-4.227845000	1.946574000	-1.247650000
H	-3.377869000	1.988459000	-1.941149000
H	-5.123265000	2.195102000	-1.831301000
H	-4.102117000	2.741625000	-0.504923000
C	-4.775062000	-0.941985000	-1.948831000
H	-3.956391000	-0.997154000	-2.676084000
H	-5.018207000	-1.961587000	-1.633387000
H	-5.649469000	-0.553815000	-2.487068000

TS3’, Imaginary frequency = 1347.3i

Y	-0.369793000	0.595459000	0.161785000
C	-2.766079000	-0.322574000	0.800018000
C	-1.823904000	-1.085664000	1.579836000
C	-1.230578000	-0.228084000	2.547439000

C	-1.800913000	1.074692000	2.397417000	C	1.552517000	3.995535000	-1.726366000
C	-2.747850000	1.018739000	1.340407000	H	0.936946000	4.694094000	-2.286780000
N	0.305670000	-0.627544000	-1.989938000	C	2.939132000	4.079965000	-1.814641000
C	1.627992000	-2.337142000	-0.711843000	H	3.394233000	4.832022000	-2.453307000
C	0.599957000	-2.022281000	-1.637113000	C	3.736448000	3.214906000	-1.064335000
C	-0.149451000	-3.045327000	-2.232257000	H	4.819198000	3.296075000	-1.111068000
H	-0.903426000	-2.812617000	-2.974912000	C	3.141297000	2.242584000	-0.264089000
C	0.054491000	-4.382312000	-1.905498000	H	3.781823000	1.564309000	0.294489000
H	-0.542037000	-5.152587000	-2.385465000	C	-1.279374000	3.293765000	-1.859303000
C	1.025774000	-4.707128000	-0.968958000	H	-1.261472000	4.364755000	-2.100642000
H	1.200636000	-5.741418000	-0.686931000	H	-2.319848000	3.010032000	-1.677095000
C	1.792263000	-3.694804000	-0.399037000	H	-0.899462000	2.747560000	-2.726350000
H	2.553933000	-3.977627000	0.317738000	C	-0.801903000	3.963259000	0.398030000
C	-1.011065000	-0.444549000	-2.630699000	H	-0.240664000	3.729819000	1.304520000
H	-1.055427000	-0.863097000	-3.645442000	H	-1.871333000	3.926162000	0.609255000
H	-1.210098000	0.628518000	-2.731213000	H	-0.532845000	4.978856000	0.075720000
H	-1.803029000	-0.904829000	-2.032720000	H	1.628581000	0.883286000	0.521120000
C	1.322779000	-0.072141000	-2.919209000	C	3.818840000	-1.932728000	0.544175000
H	2.324897000	-0.187310000	-2.513775000	C	4.927627000	-0.937399000	0.889566000
H	1.137791000	0.994318000	-3.074722000	H	4.226418000	-2.648372000	-0.183215000
H	1.270086000	-0.596066000	-3.883254000	H	3.569645000	-2.507245000	1.447566000
C	1.757952000	-0.479623000	1.016578000	C	6.181508000	-1.621033000	1.441304000
H	1.079962000	-1.152347000	1.559434000	H	5.198506000	-0.364259000	-0.010183000
H	2.444369000	-0.119408000	1.794983000	H	4.574799000	-0.206705000	1.631966000
C	2.535373000	-1.299272000	-0.039817000	C	7.297611000	-0.637458000	1.784916000
H	2.886288000	-0.604652000	-0.814616000	H	5.912419000	-2.198921000	2.336562000
N	-0.469673000	2.984265000	-0.665070000	H	6.547807000	-2.351070000	0.706138000
C	1.745024000	2.109108000	-0.164026000	H	8.178511000	-1.158113000	2.175036000
C	0.977224000	3.028314000	-0.894531000	H	7.613690000	-0.068688000	0.901825000

H	6.974189000	0.082448000	2.546767000
C	-1.576550000	-2.567330000	1.493584000
H	-1.596050000	-2.941292000	0.467101000
H	-0.607764000	-2.844572000	1.920295000
H	-2.340052000	-3.119608000	2.057927000
C	-0.341979000	-0.624587000	3.692270000
H	0.121991000	-1.603632000	3.544371000
H	0.461267000	0.098517000	3.874289000
H	-0.930407000	-0.688622000	4.617449000
C	-1.562843000	2.200174000	3.366007000
H	-1.996792000	3.145242000	3.030133000
H	-2.020564000	1.965276000	4.336193000
H	-0.497276000	2.374788000	3.559421000
C	-3.740405000	2.103127000	1.013456000
H	-4.758143000	1.774345000	1.256183000
H	-3.563381000	3.005751000	1.604516000
H	-3.751127000	2.390427000	-0.043463000
Si	-4.237562000	-1.011238000	-0.192029000
C	-5.697229000	-1.173257000	0.997007000
H	-5.993355000	-0.215469000	1.438667000
H	-6.572383000	-1.581174000	0.476253000
H	-5.453789000	-1.854508000	1.820652000
C	-4.736657000	0.128116000	-1.625414000
H	-3.905349000	0.378782000	-2.295808000
H	-5.494096000	-0.382130000	-2.233713000
H	-5.185205000	1.065194000	-1.280550000
C	-3.927233000	-2.729598000	-0.922992000
H	-3.034343000	-2.807089000	-1.552831000
H	-3.851582000	-3.497206000	-0.146096000
H	-4.789618000	-2.990525000	-1.549884000

***N,N*-dimethyl-*o*-toluidine**

C	2.695345000	-0.101735000	-0.013957000
C	2.103873000	-1.355071000	-0.161409000
C	0.718319000	-1.454229000	-0.214037000
C	-0.106505000	-0.324973000	-0.098566000
C	0.487752000	0.942306000	0.087431000
C	1.886445000	1.024153000	0.105909000
H	3.777278000	-0.000650000	0.015236000
H	2.717127000	-2.247779000	-0.256722000
H	0.238156000	-2.417348000	-0.368514000
H	2.347255000	2.000709000	0.244516000
N	-1.508974000	-0.552856000	-0.172896000
C	-2.336163000	0.289524000	-1.005714000
H	-3.202173000	-0.292024000	-1.351890000
H	-2.728525000	1.193991000	-0.508225000
H	-1.771068000	0.600606000	-1.889305000
C	-2.160169000	-1.046383000	1.022333000
H	-3.095488000	-1.554236000	0.751971000
H	-1.513172000	-1.770298000	1.525949000
H	-2.410521000	-0.251256000	1.750402000
C	-0.321832000	2.195375000	0.308456000
H	-0.766792000	2.579209000	-0.618158000
H	-1.142183000	2.025736000	1.013915000
H	0.310527000	2.989535000	0.718420000

B-sp^2

Y	0.123586000	0.208520000	-0.232794000
C	-0.338879000	2.185755000	0.974562000

H	0.467421000	2.150297000	1.736031000
H	0.028154000	2.945132000	0.256907000
C	2.760430000	-0.065361000	-0.339178000
C	2.258350000	-0.738142000	-1.513993000
C	1.703835000	0.229561000	-2.394931000
C	1.852091000	1.515237000	-1.790893000
C	2.501630000	1.341456000	-0.541747000
C	2.421502000	-2.189178000	-1.881304000
H	3.308311000	-2.331062000	-2.513787000
H	2.550325000	-2.832963000	-1.010142000
H	1.569055000	-2.571013000	-2.457199000
C	1.240690000	-0.022431000	-3.801123000
H	2.060012000	0.155066000	-4.510765000
H	0.914225000	-1.056128000	-3.953550000
H	0.418284000	0.636374000	-4.099578000
C	1.543769000	2.837004000	-2.435564000
H	2.459708000	3.291636000	-2.836611000
H	0.851068000	2.736591000	-3.277846000
H	1.116388000	3.560110000	-1.731685000
C	2.989900000	2.484990000	0.301472000
H	3.913245000	2.902542000	-0.121353000
H	2.262920000	3.302174000	0.348367000
H	3.207962000	2.186349000	1.330154000
Si	-1.783073000	3.046884000	1.825011000
C	-1.273681000	4.790020000	2.362461000
H	-0.431810000	4.757855000	3.065056000
H	-0.962625000	5.399782000	1.505019000
H	-2.098513000	5.315890000	2.859301000
C	-2.362357000	2.130018000	3.383699000
H	-3.112622000	2.733961000	3.908981000
H	-2.826318000	1.157292000	3.179592000
H	-1.534393000	1.971786000	4.086681000
C	-3.265174000	3.226950000	0.649588000
H	-2.988346000	3.781141000	-0.256527000
H	-3.679256000	2.258497000	0.340142000
H	-4.076375000	3.785230000	1.132540000
C	-2.164275000	1.245170000	-2.549218000
O	-1.537737000	0.082242000	-1.921450000
C	-2.063488000	-1.146114000	-2.510045000
C	-3.251637000	-0.707044000	-3.352718000
C	-2.843262000	0.700413000	-3.795056000
H	-2.880434000	1.665058000	-1.837347000
H	-1.378186000	1.977476000	-2.748173000
H	-1.267906000	-1.589596000	-3.117753000
H	-2.331423000	-1.821655000	-1.693536000
H	-3.429263000	-1.385616000	-4.191306000
H	-4.159389000	-0.672169000	-2.740934000
H	-2.139771000	0.654029000	-4.633427000
H	-3.693901000	1.317526000	-4.096823000
Si	4.109894000	-0.659429000	0.869575000
C	5.689176000	0.297222000	0.471734000
H	5.612278000	1.364019000	0.703304000
H	6.526120000	-0.106253000	1.055017000
H	5.948375000	0.203092000	-0.589315000
C	4.541177000	-2.494949000	0.691315000
H	4.902447000	-2.743434000	-0.312201000
H	5.361788000	-2.707184000	1.388941000
H	3.729051000	-3.187348000	0.941453000

C	3.657579000	-0.386579000	2.693450000
H	4.572265000	-0.426316000	3.298315000
H	3.181403000	0.579385000	2.895062000
H	2.999049000	-1.177373000	3.073587000
N	-0.392996000	-1.670562000	1.469315000
C	-2.596415000	-0.851225000	0.978075000
C	-1.789924000	-1.984786000	1.169734000
C	-2.372990000	-3.271964000	1.092965000
C	-3.733313000	-3.334958000	0.746383000
H	-4.193746000	-4.317422000	0.678352000
C	-4.520432000	-2.208426000	0.532835000
H	-5.573618000	-2.319007000	0.291515000
C	-3.949565000	-0.947807000	0.671147000
H	-4.545550000	-0.047491000	0.555398000
C	0.604217000	-2.722490000	1.203276000
H	0.576421000	-3.531180000	1.940200000
H	1.595606000	-2.266789000	1.242349000
H	0.455495000	-3.141104000	0.205145000
C	-0.243415000	-1.188790000	2.865765000
H	-0.924155000	-0.358007000	3.053851000
H	0.783142000	-0.846035000	3.019696000
H	-0.459857000	-1.994946000	3.579790000
H	-2.167624000	0.142983000	1.127171000
C	-1.701008000	-4.586956000	1.422014000
H	-1.252507000	-4.581439000	2.420530000
H	-2.448249000	-5.384812000	1.417614000
H	-0.924335000	-4.875168000	0.706728000

TS1-sp^2, Imaginary frequency = 1300.79i

Y	0.028149000	-0.456068000	-0.169853000
C	-0.106895000	1.862692000	-1.048493000
H	0.895923000	2.033224000	-0.614916000
H	0.074471000	1.483522000	-2.072503000
C	2.680576000	-0.593677000	-0.007384000
C	2.209071000	-1.912412000	0.343914000
C	1.613047000	-2.508032000	-0.801576000
C	1.726767000	-1.586876000	-1.886565000
C	2.385375000	-0.423064000	-1.408676000
C	2.512249000	-2.686641000	1.597802000
H	3.429394000	-3.275650000	1.459805000
H	2.681164000	-2.043965000	2.462576000
H	1.721498000	-3.401476000	1.851968000
C	1.127349000	-3.926138000	-0.905445000
H	1.950747000	-4.599927000	-1.178873000
H	0.718805000	-4.299683000	0.040355000
H	0.354503000	-4.041827000	-1.672497000
C	1.394620000	-1.876576000	-3.322811000
H	2.280680000	-2.251799000	-3.852648000
H	0.621127000	-2.645160000	-3.421027000
H	1.062499000	-0.985207000	-3.867893000
C	2.856875000	0.688145000	-2.304374000
H	3.816581000	0.420089000	-2.765697000
H	2.158544000	0.886009000	-3.124825000
H	3.003404000	1.629636000	-1.770734000
Si	-0.812830000	3.614250000	-1.286159000
C	0.540741000	4.720272000	-2.005373000
H	1.406939000	4.785298000	-1.335692000
H	0.895085000	4.346072000	-2.973556000
H	0.171652000	5.741256000	-2.162808000

C	-1.374616000	4.323161000	0.373367000
H	-1.782604000	5.332477000	0.240004000
H	-2.155533000	3.711339000	0.839850000
H	-0.538958000	4.407499000	1.079801000
C	-2.260555000	3.579698000	-2.503818000
H	-1.987796000	3.080235000	-3.441883000
H	-3.147584000	3.081191000	-2.097766000
H	-2.558030000	4.604032000	-2.759376000
C	-2.190812000	-1.470450000	-2.602068000
O	-1.597389000	-1.800489000	-1.313361000
C	-2.464987000	-2.731155000	-0.596325000
C	-3.691114000	-2.919656000	-1.483798000
C	-3.149779000	-2.613237000	-2.882805000
H	-2.716173000	-0.513140000	-2.509285000
H	-1.376899000	-1.376317000	-3.323243000
H	-1.898351000	-3.657547000	-0.460408000
H	-2.707000000	-2.293418000	0.377358000
H	-4.106407000	-3.927032000	-1.395391000
H	-4.470727000	-2.201618000	-1.209803000
H	-2.612117000	-3.474967000	-3.293790000
H	-3.932573000	-2.327681000	-3.590716000
Si	3.926848000	0.506616000	0.925114000
C	5.591386000	0.366211000	0.043917000
H	5.555838000	0.740180000	-0.984821000
H	6.354644000	0.945208000	0.578292000
H	5.930443000	-0.675710000	0.006664000
C	4.202763000	-0.008913000	2.726995000
H	4.661426000	-0.998684000	2.821431000
H	4.901376000	0.710925000	3.172588000
H	3.296754000	0.007567000	3.344474000
C	3.407539000	2.329950000	0.978866000
H	4.154468000	2.887220000	1.558864000
H	3.356663000	2.802493000	-0.007550000
H	2.440960000	2.481536000	1.473299000
N	-0.584022000	-0.103217000	2.267823000
C	-2.131609000	0.607000000	0.583251000
C	-1.987163000	0.146759000	1.904408000
C	-3.094589000	-0.030959000	2.764632000
C	-4.360236000	0.217767000	2.215239000
H	-5.231449000	0.093826000	2.854395000
C	-4.536006000	0.645503000	0.901073000
H	-5.537879000	0.839827000	0.526354000
C	-3.420381000	0.856426000	0.097434000
H	-3.551358000	1.236367000	-0.913978000
C	-0.326686000	-1.282808000	3.112501000
H	-0.594496000	-1.122368000	4.161236000
H	0.737639000	-1.518322000	3.070533000
H	-0.892124000	-2.141292000	2.736536000
C	0.043523000	1.099548000	2.855791000
H	-0.108535000	1.955133000	2.193865000
H	1.115719000	0.925659000	2.983185000
H	-0.392597000	1.337878000	3.835201000
H	-1.175639000	1.278572000	-0.194906000
C	-3.031218000	-0.414902000	4.224730000
H	-2.281395000	0.159436000	4.777866000
H	-3.996531000	-0.216937000	4.699345000
H	-2.814398000	-1.478821000	4.375291000

C-sp^2

Y	0.050600000	0.191764000	-0.097770000
C	-2.426387000	-0.415806000	-0.458591000
C	-2.321040000	0.969121000	-0.862156000
C	-1.540631000	1.041949000	-2.045870000
C	-1.149908000	-0.287024000	-2.402588000
C	-1.705090000	-1.179935000	-1.442922000
C	-3.023054000	2.141937000	-0.230781000
H	-4.034507000	2.254050000	-0.642155000
H	-3.132604000	2.039204000	0.853695000
H	-2.502802000	3.086392000	-0.424973000
C	-1.267223000	2.271467000	-2.866322000
H	-1.982252000	2.349509000	-3.696053000
H	-1.359989000	3.192808000	-2.282178000
H	-0.267322000	2.259783000	-3.315613000
C	-0.442942000	-0.679351000	-3.667444000
H	-1.169125000	-0.929015000	-4.452804000
H	0.180797000	0.131934000	-4.055912000
H	0.202677000	-1.551354000	-3.528098000
C	-1.609083000	-2.676351000	-1.559899000
H	-2.429960000	-3.064786000	-2.177883000
H	-0.676847000	-2.984281000	-2.045270000
H	-1.671455000	-3.184227000	-0.595494000
C	2.083004000	2.861748000	0.077672000
O	0.857148000	2.286874000	0.653140000
C	0.276920000	3.222947000	1.614178000
C	1.386280000	4.218495000	1.908052000
C	2.106193000	4.303836000	0.559642000
H	2.925716000	2.273593000	0.452177000
H	2.025850000	2.754125000	-1.007897000
H	-0.589668000	3.697995000	1.143512000
H	-0.050323000	2.647723000	2.484772000
H	0.991609000	5.180664000	2.245014000
H	2.056786000	3.832708000	2.684131000
H	1.556017000	4.952301000	-0.131105000
H	3.127159000	4.685624000	0.643159000
Si	-3.465078000	-0.998723000	1.017966000
C	-5.228702000	-0.344396000	0.880893000
H	-5.688096000	-0.664386000	-0.061747000
H	-5.839937000	-0.745745000	1.698581000
H	-5.292581000	0.747078000	0.928013000
C	-2.664714000	-0.340276000	2.619332000
H	-2.598553000	0.754599000	2.651628000
H	-3.255250000	-0.645423000	3.492453000
H	-1.655618000	-0.750918000	2.775355000
C	-3.566962000	-2.876533000	1.188595000
H	-4.257159000	-3.106722000	2.010222000
H	-3.965706000	-3.355761000	0.288190000
H	-2.610813000	-3.353328000	1.432332000
N	1.443507000	-1.327954000	1.298316000
C	2.041834000	-0.646859000	-0.940465000
C	2.498764000	-1.244898000	0.251578000
C	3.805278000	-1.754716000	0.413530000
C	4.653754000	-1.636034000	-0.694505000
H	5.667835000	-2.021377000	-0.612515000
C	4.239639000	-1.057463000	-1.894193000
H	4.933961000	-0.999745000	-2.729246000
C	2.942346000	-0.568334000	-2.013890000
H	2.629946000	-0.124603000	-2.958135000

C	1.801272000	-0.766170000	2.613336000
H	2.485896000	-1.408052000	3.178422000
H	0.893027000	-0.642424000	3.216027000
H	2.276441000	0.209906000	2.478534000
C	0.893137000	-2.691228000	1.439675000
H	0.632555000	-3.081674000	0.453306000
H	-0.005962000	-2.662241000	2.065731000
H	1.608674000	-3.380521000	1.904594000
C	4.358802000	-2.396678000	1.663926000
H	3.674066000	-3.116188000	2.122125000
H	5.280201000	-2.938317000	1.430489000
H	4.614362000	-1.651571000	2.428099000

D-sp^2

Y	0.263331000	-0.281990000	-0.335736000
C	2.777635000	0.748709000	-0.142251000
C	2.930830000	-0.599967000	-0.623970000
C	2.365337000	-0.692892000	-1.927275000
C	1.887329000	0.601250000	-2.293279000
C	2.139542000	1.483263000	-1.209687000
C	3.798245000	-1.683280000	-0.044260000
H	4.821794000	-1.583441000	-0.431573000
H	3.869600000	-1.641303000	1.043426000
H	3.458247000	-2.684908000	-0.327907000
C	2.471608000	-1.878625000	-2.845344000
H	3.470854000	-1.928007000	-3.299095000
H	2.315458000	-2.833848000	-2.330092000
H	1.752863000	-1.820089000	-3.668066000
C	1.400741000	0.998125000	-3.657215000
H	2.247505000	1.253152000	-4.308783000
H	0.851530000	0.189760000	-4.150112000
H	0.751649000	1.880486000	-3.629070000
C	2.012245000	2.979150000	-1.310904000
H	2.909661000	3.399818000	-1.782886000
H	1.164412000	3.289076000	-1.932616000
H	1.905846000	3.465138000	-0.337450000
C	-1.897606000	-0.236804000	-2.977433000
O	-0.859329000	-1.030335000	-2.342524000
C	-1.138679000	-2.451293000	-2.535260000
C	-2.485848000	-2.515740000	-3.243883000
C	-2.545238000	-1.175487000	-3.981800000
H	-2.611416000	0.087517000	-2.210873000
H	-1.416676000	0.635735000	-3.425441000
H	-0.325335000	-2.860373000	-3.141491000
H	-1.152375000	-2.931024000	-1.551803000
H	-2.552425000	-3.377003000	-3.914027000
H	-3.296171000	-2.587486000	-2.511169000
H	-1.959672000	-1.210221000	-4.907373000
H	-3.564440000	-0.869365000	-4.233208000
Si	3.742605000	1.601333000	1.263807000
C	5.056764000	2.710311000	0.479233000
H	4.628992000	3.550611000	-0.077122000
H	5.706015000	3.129869000	1.257685000
H	5.692537000	2.142794000	-0.210449000
C	4.663656000	0.407030000	2.408817000
H	5.451980000	-0.149171000	1.891225000
H	5.155436000	1.012420000	3.181318000
H	4.026583000	-0.313657000	2.934333000
C	2.646536000	2.661372000	2.393669000

H	3.284959000	3.129315000	3.153883000
H	2.127569000	3.475985000	1.876583000
H	1.899015000	2.066920000	2.933276000
N	0.333861000	-1.744395000	1.746872000
C	-1.712621000	-1.262610000	0.617534000
C	-1.131135000	-1.965201000	1.683899000
C	-1.849846000	-2.788991000	2.579527000
C	-3.226326000	-2.895350000	2.343836000
H	-3.820409000	-3.516502000	3.010858000
C	-3.850367000	-2.236935000	1.285436000
H	-4.921587000	-2.355576000	1.136513000
C	-3.097247000	-1.430203000	0.437510000
H	-3.613109000	-0.925816000	-0.380693000
C	1.113997000	-2.994065000	1.720177000
H	1.005016000	-3.572488000	2.643448000
H	2.171083000	-2.762755000	1.587422000
H	0.776387000	-3.615250000	0.884969000
C	0.739186000	-0.893349000	2.879166000
H	0.148243000	0.028732000	2.873907000
H	1.798978000	-0.639805000	2.783770000
H	0.586187000	-1.378866000	3.851026000
C	-0.598050000	2.238915000	0.485799000
H	-0.460519000	2.823036000	-0.421940000
H	0.263601000	2.167949000	1.150963000
C	-1.832555000	1.871271000	0.890718000
H	-1.949414000	1.376775000	1.855235000
C	-3.101032000	2.197785000	0.174586000
C	-3.913194000	3.251944000	0.954516000
H	-3.711215000	1.291208000	0.079028000
H	-2.885636000	2.573216000	-0.834391000
C	-5.239411000	3.591229000	0.270900000
H	-4.109299000	2.881160000	1.970245000
H	-3.309703000	4.162628000	1.065922000
C	-6.043493000	4.640747000	1.034713000
H	-5.040674000	3.950368000	-0.748770000
H	-5.836123000	2.674344000	0.164346000
H	-6.987438000	4.860972000	0.525195000
H	-6.284545000	4.297990000	2.047833000
H	-5.487072000	5.581165000	1.124316000
C	-1.281274000	-3.578226000	3.736565000
H	-0.520828000	-3.039051000	4.307893000
H	-2.080268000	-3.839639000	4.437237000
H	-0.832812000	-4.522595000	3.402805000

TS2-sp^2, Imaginary frequency = 232.70i

Y	-0.188188000	-0.227979000	0.245796000
C	-2.785408000	0.414902000	0.372511000
C	-2.753552000	-1.016849000	0.559499000
C	-2.108132000	-1.306065000	1.791879000
C	-1.751919000	-0.067115000	2.404166000
C	-2.170550000	0.984794000	1.547690000
C	-3.501249000	-2.065957000	-0.220511000
H	-4.434326000	-2.322084000	0.299923000
H	-3.781063000	-1.733424000	-1.220069000
H	-2.938199000	-3.001238000	-0.323714000
C	-2.023248000	-2.659212000	2.438399000
H	-2.903872000	-2.843867000	3.068921000
H	-1.989777000	-3.470866000	1.703148000

H	-1.144357000	-2.756373000	3.084295000
C	-1.218628000	0.103447000	3.799101000
H	-2.031296000	0.353667000	4.494504000
H	-0.756075000	-0.814372000	4.175694000
H	-0.482902000	0.913254000	3.879923000
C	-2.159484000	2.430551000	1.958574000
H	-3.077904000	2.678534000	2.507519000
H	-1.323871000	2.660164000	2.628532000
H	-2.094675000	3.115069000	1.110548000
C	2.016424000	-1.284207000	2.763154000
O	1.138442000	-1.675276000	1.676574000
C	1.426571000	-3.049240000	1.280694000
C	2.570712000	-3.509782000	2.181085000
C	2.439104000	-2.595046000	3.402155000
H	2.874774000	-0.743144000	2.346896000
H	1.450845000	-0.621354000	3.420699000
H	0.510833000	-3.626853000	1.439255000
H	1.692735000	-3.052690000	0.219205000
H	2.490827000	-4.571144000	2.430545000
H	3.533452000	-3.348657000	1.684881000
H	1.661154000	-2.957889000	4.083089000
H	3.370625000	-2.498276000	3.966509000
Si	-3.897225000	1.431078000	-0.791324000
C	-5.379938000	2.025724000	0.216883000
H	-5.080823000	2.668943000	1.051570000
H	-6.071276000	2.600164000	-0.411895000
H	-5.935861000	1.178407000	0.635181000
C	-4.579187000	0.462128000	-2.271300000
H	-5.287306000	-0.320821000	-1.980701000
H	-5.131905000	1.171698000	-2.900556000
H	-3.811291000	0.008958000	-2.909868000
C	-3.026951000	2.943101000	-1.539690000
H	-3.725161000	3.431494000	-2.231732000
H	-2.737527000	3.698158000	-0.801891000
H	-2.134522000	2.682672000	-2.120695000
N	-0.104291000	-0.668264000	-2.245971000
C	1.955493000	-0.385731000	-1.004854000
C	1.312772000	-1.024509000	-2.092117000
C	1.979571000	-1.965020000	-2.916363000
C	3.284822000	-2.316362000	-2.555012000
H	3.820636000	-3.031416000	-3.174967000
C	3.914383000	-1.767659000	-1.440748000
H	4.923876000	-2.077516000	-1.181158000
C	3.259413000	-0.795182000	-0.693775000
H	3.783770000	-0.351376000	0.148481000
C	-1.014739000	-1.785281000	-2.556716000
H	-0.978397000	-2.090654000	-3.606999000
H	-2.034026000	-1.469173000	-2.332475000
H	-0.770540000	-2.652606000	-1.935112000
C	-0.334843000	0.436437000	-3.197535000
H	0.268270000	1.304543000	-2.930251000
H	-1.390283000	0.719348000	-3.168715000
H	-0.079592000	0.140386000	-4.223519000
C	0.516450000	2.070828000	0.122691000
H	0.615592000	2.474444000	1.131726000
H	-0.320612000	2.484493000	-0.442265000
C	1.703905000	1.747970000	-0.573261000
H	1.692396000	1.867074000	-1.654544000

C	3.038919000	2.106124000	0.035441000
C	3.237769000	3.633235000	0.009691000
H	3.859733000	1.644350000	-0.519988000
H	3.100793000	1.754280000	1.075296000
C	4.610782000	4.052200000	0.539789000
H	3.119438000	3.995386000	-1.021727000
H	2.448171000	4.118297000	0.597583000
C	4.814565000	5.565596000	0.516829000
H	4.732073000	3.680470000	1.567498000
H	5.395569000	3.566404000	-0.056776000
H	5.802616000	5.836908000	0.903351000
H	4.736169000	5.961651000	-0.502509000
H	4.064695000	6.077753000	1.131421000
C	1.419383000	-2.586860000	-4.175342000
H	0.866341000	-1.872898000	-4.792319000
H	2.240061000	-2.969086000	-4.789471000
H	0.755702000	-3.434821000	-3.968630000

E-sp^2

Y	-0.248778000	-0.535603000	-0.291796000
C	2.120143000	-1.582912000	0.111315000
C	1.195043000	-2.686879000	0.206163000
C	0.621146000	-2.913747000	-1.075103000
C	1.185058000	-1.968657000	-1.987687000
C	2.104320000	-1.162030000	-1.270525000
C	0.976025000	-3.586967000	1.392931000
H	1.687317000	-4.424033000	1.379768000
H	1.114145000	-3.072939000	2.346650000
H	-0.026113000	-4.031045000	1.398289000
C	-0.270442000	-4.059687000	-1.463638000
H	0.314742000	-4.855614000	-1.943185000
H	-0.764160000	-4.512898000	-0.598150000
H	-1.047531000	-3.766815000	-2.179156000
C	0.924618000	-1.922935000	-3.466256000
H	1.689028000	-2.488888000	-4.015611000
H	-0.042733000	-2.367174000	-3.725263000
H	0.933071000	-0.899972000	-3.855009000
C	3.003540000	-0.138258000	-1.905578000
H	4.033718000	-0.512932000	-1.968755000
H	2.685831000	0.089279000	-2.926701000
H	3.036235000	0.805735000	-1.354015000
C	-3.077787000	-0.953186000	-2.021337000
O	-2.425540000	-1.383069000	-0.780013000
C	-3.284898000	-2.323289000	-0.071664000
C	-4.627139000	-2.263348000	-0.786347000
C	-4.217825000	-1.936951000	-2.224283000
H	-3.429098000	0.071918000	-1.869660000
H	-2.325305000	-0.965883000	-2.812674000
H	-2.824619000	-3.314016000	-0.136951000
H	-3.337985000	-2.011990000	0.975238000
H	-5.176941000	-3.204174000	-0.698333000
H	-5.248689000	-1.462551000	-0.371095000
H	-3.866893000	-2.836759000	-2.741721000
H	-5.030649000	-1.501518000	-2.811758000
Si	3.542107000	-1.172895000	1.302713000
C	5.046205000	-2.175494000	0.753432000
H	5.336544000	-1.943517000	-0.277511000
H	5.909282000	-1.963591000	1.396535000
H	4.848184000	-3.252417000	0.805670000

C	3.193566000	-1.628755000	3.110523000
H	3.147706000	-2.710950000	3.270449000
H	4.028800000	-1.254214000	3.716208000
H	2.279946000	-1.185615000	3.524443000
C	4.006106000	0.665842000	1.278821000
H	4.663712000	0.878395000	2.131306000
H	4.558057000	0.940844000	0.374251000
H	3.143370000	1.337555000	1.358976000
N	-0.598889000	0.667900000	1.983272000
C	-1.561428000	2.063096000	0.215183000
C	-1.745536000	1.417765000	1.468104000
C	-2.985904000	1.516715000	2.148772000
C	-4.049676000	2.144671000	1.493778000
H	-5.002835000	2.223291000	2.011013000
C	-3.909161000	2.698951000	0.225955000
H	-4.753331000	3.185150000	-0.255142000
C	-2.668916000	2.672842000	-0.392247000
H	-2.539193000	3.150319000	-1.358924000
C	-0.880123000	-0.548919000	2.766015000
H	-1.158686000	-0.349716000	3.805647000
H	0.024163000	-1.164708000	2.779777000
H	-1.692548000	-1.122115000	2.305710000
C	0.353688000	1.519135000	2.726009000
H	0.599208000	2.409510000	2.145646000
H	1.270786000	0.956517000	2.917881000
H	-0.074216000	1.839892000	3.685374000
C	-0.224065000	1.273896000	-1.774788000
H	-1.101403000	1.496460000	-2.396520000
H	0.671709000	1.443826000	-2.381083000
C	-0.217755000	2.174670000	-0.522303000
H	0.579566000	1.809457000	0.162417000
C	0.151596000	3.666200000	-0.740742000
C	1.593447000	3.895425000	-1.190647000
H	-0.026956000	4.220711000	0.192988000
H	-0.530776000	4.091197000	-1.487802000
C	1.924581000	5.377247000	-1.385531000
H	2.283801000	3.466756000	-0.447290000
H	1.780008000	3.360334000	-2.131234000
C	3.364658000	5.612265000	-1.835314000
H	1.234471000	5.805857000	-2.125709000
H	1.739423000	5.918501000	-0.446947000
H	3.569293000	6.679935000	-1.967945000
H	4.080338000	5.226573000	-1.098976000
H	3.569333000	5.114264000	-2.790853000
C	-3.234605000	1.065269000	3.571339000
H	-2.425451000	1.353751000	4.249168000
H	-4.144197000	1.542799000	3.945908000
H	-3.382996000	-0.016345000	3.667846000

B-sp^3

Y	0.182261000	0.462185000	-0.245246000
C	-0.681029000	0.049523000	1.901648000
H	-0.138450000	-0.864079000	2.209493000
H	-0.218648000	0.838482000	2.521282000
C	2.685300000	-0.264867000	0.205622000
C	2.700455000	0.227659000	-1.149783000
C	2.435224000	1.623125000	-1.136557000
C	2.274308000	2.028017000	0.225368000
C	2.439600000	0.879384000	1.048195000

C	3.178032000	-0.500507000	-2.376508000
H	4.275131000	-0.467326000	-2.428376000
H	2.892848000	-1.553583000	-2.393703000
H	2.814605000	-0.031953000	-3.298114000
C	2.537945000	2.538942000	-2.326071000
H	3.587625000	2.665093000	-2.623486000
H	2.008459000	2.164778000	-3.211295000
H	2.154015000	3.540193000	-2.109092000
C	2.160664000	3.447119000	0.707066000
H	3.157266000	3.893080000	0.825609000
H	1.611393000	4.086227000	0.007688000
H	1.667095000	3.518968000	1.681525000
C	2.531967000	0.917094000	2.547434000
H	3.582995000	0.937300000	2.864782000
H	2.060329000	1.812796000	2.963454000
H	2.058582000	0.053619000	3.019372000
Si	-2.445481000	-0.138215000	2.560508000
C	-2.765632000	1.171778000	3.895947000
H	-2.063589000	1.057762000	4.731055000
H	-2.660161000	2.197118000	3.518559000
H	-3.780029000	1.079978000	4.303240000
C	-2.664872000	-1.846144000	3.350514000
H	-3.671327000	-1.962990000	3.770713000
H	-2.521530000	-2.652529000	2.620998000
H	-1.945993000	-1.999473000	4.164491000
C	-3.809091000	0.043091000	1.247839000
H	-3.797700000	1.013220000	0.733540000
H	-3.740425000	-0.737729000	0.481943000
H	-4.795638000	-0.052517000	1.719335000
C	-1.521896000	3.170247000	0.726115000
O	-0.994244000	2.543823000	-0.498720000
C	-1.046213000	3.511681000	-1.587356000
C	-2.064777000	4.545773000	-1.144255000
C	-1.798324000	4.621487000	0.359901000
H	-2.426060000	2.624002000	0.999170000
H	-0.776805000	3.048475000	1.513859000
H	-0.049638000	3.947229000	-1.711318000
H	-1.321376000	2.979165000	-2.499586000
H	-1.925666000	5.502392000	-1.655184000
H	-3.083127000	4.193722000	-1.344143000
H	-0.923544000	5.248447000	0.563942000
H	-2.642855000	5.024567000	0.925175000
Si	3.481983000	-1.877817000	0.836918000
C	5.277805000	-1.470396000	1.260950000
H	5.349593000	-0.715900000	2.052402000
H	5.803057000	-2.367176000	1.612140000
H	5.820137000	-1.087752000	0.388545000
C	3.494858000	-3.260805000	-0.458271000
H	4.182761000	-3.057458000	-1.285142000
H	3.845961000	-4.178552000	0.030395000
H	2.508038000	-3.482477000	-0.880583000
C	2.637618000	-2.576099000	2.381627000
H	3.015792000	-3.591075000	2.558131000
H	2.859368000	-1.993250000	3.281291000
H	1.548255000	-2.646531000	2.288706000
N	-0.854273000	-0.954750000	-2.221514000
C	-1.766545000	-2.729602000	-0.750223000
C	-1.985751000	-1.747963000	-1.736657000

C	-3.267272000	-1.579620000	-2.276746000
C	-4.331094000	-2.371642000	-1.854883000
H	-5.314982000	-2.222771000	-2.289912000
C	-4.124503000	-3.349255000	-0.886477000
H	-4.945110000	-3.975754000	-0.550000000
C	-2.854186000	-3.513857000	-0.346243000
H	-2.689607000	-4.274342000	0.413482000
C	-1.259353000	0.210466000	-3.027561000
H	-1.706963000	-0.076245000	-3.989256000
H	-0.367912000	0.807442000	-3.254916000
H	-1.984535000	0.819316000	-2.479392000
C	0.009020000	-1.806595000	-3.079106000
H	0.426048000	-2.633667000	-2.505560000
H	0.828828000	-1.209369000	-3.481970000
H	-0.578923000	-2.216873000	-3.911008000
H	-3.454617000	-0.836534000	-3.043353000
C	-0.427104000	-3.018006000	-0.124803000
H	0.318998000	-2.223361000	-0.256887000
H	-0.532589000	-3.187335000	0.950270000
H	0.024350000	-3.923020000	-0.551648000

TS1-sp^3, Imaginary frequency = 1291.37i

Y	-0.092712000	-0.397759000	0.159618000
C	0.346574000	2.070753000	0.353053000
H	-0.679186000	2.466031000	0.351653000
H	0.541402000	1.786599000	1.405873000
C	-2.709994000	-0.192621000	0.273707000
C	-2.458806000	-1.613617000	0.295044000
C	-1.802239000	-1.946216000	1.512749000
C	-1.671689000	-0.749820000	2.282213000
C	-2.241728000	0.316859000	1.539794000
C	-3.023089000	-2.630594000	-0.657554000
H	-4.053384000	-2.878130000	-0.367836000
H	-3.063294000	-2.273706000	-1.687191000
H	-2.467958000	-3.573970000	-0.643830000
C	-1.495964000	-3.321108000	2.039010000
H	-2.237081000	-3.614637000	2.794721000
H	-1.524219000	-4.087086000	1.259222000
H	-0.514945000	-3.378904000	2.527289000
C	-1.236403000	-0.700300000	3.718741000
H	-2.051796000	-1.035215000	4.374689000
H	-0.382310000	-1.356384000	3.922410000
H	-0.975807000	0.313644000	4.039083000
C	-2.500037000	1.681086000	2.115348000
H	-3.348411000	1.635832000	2.811163000
H	-1.646211000	2.075528000	2.677393000
H	-2.752511000	2.417428000	1.350010000
Si	1.462458000	3.579657000	0.049600000
C	1.038346000	4.906488000	1.329391000
H	-0.006201000	5.229017000	1.241377000
H	1.187890000	4.546458000	2.354974000
H	1.668327000	5.795217000	1.199810000
C	1.157680000	4.300232000	-1.672888000
H	1.648534000	5.277273000	-1.759241000
H	1.550131000	3.672554000	-2.480927000
H	0.088365000	4.456781000	-1.859464000
C	3.292471000	3.129194000	0.242500000
H	3.547313000	2.910227000	1.287354000

H	3.577091000	2.263175000	-0.366010000
H	3.924324000	3.971782000	-0.064595000
C	2.129547000	0.114933000	2.727250000
O	1.769445000	-0.825058000	1.662782000
C	2.733734000	-1.924865000	1.652521000
C	3.838091000	-1.516039000	2.615571000
C	3.086566000	-0.646935000	3.624514000
H	2.608612000	0.979421000	2.258881000
H	1.208071000	0.424528000	3.222747000
H	2.210658000	-2.825093000	1.994785000
H	3.078442000	-2.057941000	0.624789000
H	4.322744000	-2.384580000	3.069630000
H	4.603494000	-0.929766000	2.095449000
H	2.537383000	-1.266880000	4.342211000
H	3.741878000	0.025889000	4.184209000
Si	-3.836114000	0.792715000	-0.908838000
C	-5.454681000	1.150267000	-0.003458000
H	-5.304003000	1.761626000	0.893030000
H	-6.147783000	1.691356000	-0.659311000
H	-5.948624000	0.222434000	0.308249000
C	-4.249503000	-0.141887000	-2.502027000
H	-4.862408000	-1.032785000	-2.332426000
H	-4.834480000	0.532972000	-3.139923000
H	-3.363935000	-0.434939000	-3.079083000
C	-3.057386000	2.425884000	-1.472464000
H	-3.741195000	2.905172000	-2.184896000
H	-2.886113000	3.145678000	-0.665367000
H	-2.105429000	2.270495000	-1.993163000
N	0.507883000	-2.199166000	-1.673190000
C	1.685236000	-0.138839000	-2.282746000
C	1.760778000	-1.524105000	-1.996334000
C	2.980102000	-2.206590000	-2.073749000
C	4.151533000	-1.529414000	-2.404345000
H	5.090921000	-2.071359000	-2.462851000
C	4.100218000	-0.163652000	-2.679954000
H	5.003629000	0.373460000	-2.954960000
C	2.886615000	0.511576000	-2.619990000
H	2.851785000	1.571481000	-2.857306000
C	0.675236000	-3.492103000	-0.989460000
H	1.140239000	-4.249027000	-1.636302000
H	-0.309808000	-3.860383000	-0.699588000
H	1.289631000	-3.383337000	-0.092770000
C	-0.250628000	-2.460627000	-2.923561000
H	-0.397969000	-1.540240000	-3.488375000
H	-1.221003000	-2.895569000	-2.679772000
H	0.309044000	-3.162863000	-3.556284000
H	3.023433000	-3.275675000	-1.891203000
C	0.423400000	0.639647000	-2.147260000
H	-0.479415000	0.137240000	-2.513687000
H	0.402014000	1.334344000	-0.938046000
H	0.482466000	1.583345000	-2.695988000

C-sp^3

Y	0.105978000	-0.293099000	0.163405000
C	-2.457857000	-0.126934000	-0.023474000
C	-2.189119000	-1.539314000	-0.001266000
C	-1.686677000	-1.885941000	1.286145000
C	-1.669783000	-0.702138000	2.091652000
C	-2.153523000	0.370584000	1.297747000

C	-2.488409000	-2.542066000	-1.079815000	Si	-3.180507000	0.946397000	-1.415531000
H	-3.528603000	-2.886058000	-1.004479000	C	-4.760439000	1.791415000	-0.819542000
H	-2.349804000	-2.133707000	-2.081476000	H	-4.588783000	2.511539000	-0.012919000
H	-1.855869000	-3.431881000	-0.994691000	H	-5.225691000	2.335467000	-1.650851000
C	-1.447424000	-3.278632000	1.799430000	H	-5.488541000	1.054481000	-0.460584000
H	-2.361641000	-3.679626000	2.257489000	C	-3.592596000	-0.013449000	-2.987168000
H	-1.164212000	-3.974805000	1.003951000	H	-4.305359000	-0.826470000	-2.814242000
H	-0.670960000	-3.315959000	2.572397000	H	-4.059831000	0.682130000	-3.696064000
C	-1.357234000	-0.662172000	3.561673000	H	-2.707181000	-0.429579000	-3.480137000
H	-2.242994000	-0.929205000	4.153689000	C	-1.899698000	2.268594000	-1.903379000
H	-0.570187000	-1.372187000	3.841133000	H	-2.315086000	2.917932000	-2.684513000
H	-1.042972000	0.331732000	3.897502000	H	-1.605822000	2.921138000	-1.071904000
C	-2.453293000	1.749537000	1.820010000	H	-0.994454000	1.810813000	-2.327026000
H	-3.511598000	1.827583000	2.099522000	N	1.932527000	-1.971419000	0.341482000
H	-1.877970000	1.981768000	2.721984000	C	1.974051000	-0.772751000	-1.819072000
H	-2.259974000	2.537995000	1.085421000	C	2.663583000	-1.150003000	-0.624235000
C	1.916598000	2.551983000	-0.119795000	C	3.991681000	-0.756622000	-0.398927000
O	1.139547000	1.741470000	0.830058000	C	4.664480000	0.034049000	-1.321480000
C	1.174388000	2.368144000	2.149016000	H	5.697136000	0.318029000	-1.141796000
C	2.315688000	3.367977000	2.080339000	C	4.004131000	0.440013000	-2.487628000
C	2.240826000	3.837580000	0.625478000	H	4.524320000	1.047581000	-3.223463000
H	2.810282000	1.979669000	-0.382338000	C	2.694443000	0.053093000	-2.718620000
H	1.300573000	2.703089000	-1.009854000	H	2.197397000	0.354214000	-3.637725000
H	0.209833000	2.856215000	2.315190000	C	2.446577000	-1.901338000	1.720012000
H	1.315454000	1.578225000	2.891836000	H	3.425525000	-2.387535000	1.828555000
H	2.189021000	4.180694000	2.800576000	H	1.744316000	-2.416053000	2.381839000
H	3.274513000	2.877304000	2.281994000	H	2.550721000	-0.858279000	2.041536000
H	1.439766000	4.574091000	0.498530000	C	1.865694000	-3.390685000	-0.074167000
H	3.173381000	4.285174000	0.271732000	H	1.520922000	-3.465979000	-1.105480000

H	1.172909000	-3.927959000	0.578168000
H	2.860175000	-3.851543000	-0.005156000
H	4.512915000	-1.082457000	0.495729000
C	0.555929000	-1.082132000	-2.035413000
H	0.278738000	-2.137938000	-1.951344000
H	0.167451000	-0.682895000	-2.974047000

D-sp^3

Y	-0.508753000	-0.418162000	0.091140000
C	-2.024771000	1.752597000	0.212214000
C	-2.919381000	0.637056000	0.026108000
C	-2.922801000	-0.158536000	1.209377000
C	-2.041508000	0.452757000	2.149315000
C	-1.501574000	1.622720000	1.545755000
C	-3.884068000	0.451562000	-1.112643000
H	-4.757038000	1.104873000	-0.980432000
H	-3.447000000	0.694972000	-2.082535000
H	-4.269862000	-0.571863000	-1.161313000
C	-3.847069000	-1.304636000	1.516151000
H	-4.692185000	-0.962412000	2.128389000
H	-4.275399000	-1.749030000	0.613003000
H	-3.359421000	-2.109620000	2.079122000
C	-1.931189000	0.106950000	3.608378000
H	-2.587444000	0.759058000	4.201095000
H	-2.239547000	-0.921682000	3.818358000
H	-0.919012000	0.245468000	4.007957000
C	-0.747620000	2.665457000	2.325206000
H	-1.451781000	3.258379000	2.924857000
H	-0.026718000	2.237962000	3.031583000
H	-0.211898000	3.366503000	1.683136000
C	-0.190948000	-2.781484000	2.665925000
O	0.263255000	-2.374381000	1.338006000
C	1.437060000	-3.166268000	0.973247000
C	1.964817000	-3.713041000	2.285667000
C	0.666593000	-3.979997000	3.050725000
H	-0.037029000	-1.935615000	3.341635000
H	-1.259018000	-3.004580000	2.606378000
H	1.110475000	-3.967246000	0.304220000
H	2.121365000	-2.514296000	0.425453000
H	2.569849000	-4.612083000	2.139927000
H	2.578720000	-2.969037000	2.806786000
H	0.206059000	-4.913415000	2.708473000
H	0.806985000	-4.048273000	4.132805000
Si	-1.918775000	3.337772000	-0.837247000
C	-3.038639000	4.624187000	-0.021650000
H	-2.716437000	4.861240000	0.998741000
H	-3.032946000	5.559195000	-0.595304000
H	-4.076390000	4.274680000	0.032422000
C	-2.504124000	3.099399000	-2.620792000
H	-3.574755000	2.884268000	-2.693694000
H	-2.328782000	4.038821000	-3.160531000
H	-1.955222000	2.316746000	-3.156951000
C	-0.164160000	4.049153000	-0.953519000
H	-0.169039000	4.849059000	-1.704996000
H	0.195335000	4.498988000	-0.021732000
H	0.564536000	3.300661000	-1.282730000
N	-1.171450000	-1.990729000	-1.839395000
C	1.043192000	-0.980738000	-2.184325000

C	0.253114000	-2.165953000	-2.105166000
C	0.815927000	-3.430011000	-2.326793000
C	2.178951000	-3.569385000	-2.556006000
H	2.603663000	-4.553470000	-2.731598000
C	2.989978000	-2.429333000	-2.570431000
H	4.056977000	-2.523477000	-2.755180000
C	2.432314000	-1.172997000	-2.388622000
H	3.064042000	-0.291165000	-2.452403000
C	-1.833656000	-3.171557000	-1.258656000
H	-1.908889000	-4.002772000	-1.973226000
H	-2.849317000	-2.891593000	-0.967044000
H	-1.290747000	-3.516174000	-0.374356000
C	-1.885742000	-1.631079000	-3.086925000
H	-1.423637000	-0.761537000	-3.554277000
H	-2.929770000	-1.406490000	-2.862958000
H	-1.840837000	-2.469338000	-3.795643000
H	0.187007000	-4.315223000	-2.322005000
C	0.466855000	0.348997000	-1.951538000
H	-0.407547000	0.593486000	-2.562090000
H	1.201952000	1.148957000	-2.048742000
C	1.846700000	0.282106000	1.717526000
H	2.488208000	-0.588782000	1.830624000
H	0.990990000	0.354139000	2.390033000
C	2.200086000	1.309117000	0.924350000
H	1.557455000	2.189040000	0.892150000
C	3.474281000	1.406251000	0.149677000
C	4.411222000	2.468003000	0.758169000
H	3.253952000	1.691732000	-0.887724000
H	3.980900000	0.433498000	0.122825000
C	5.710680000	2.628597000	-0.033213000
H	3.888244000	3.433768000	0.802136000
H	4.640929000	2.192480000	1.796294000
C	6.643565000	3.675028000	0.572069000
H	6.227109000	1.659607000	-0.082886000
H	5.471292000	2.903215000	-1.070157000
H	7.564671000	3.767321000	-0.012853000
H	6.168184000	4.662589000	0.601524000
H	6.926226000	3.410664000	1.597964000

TS2-sp^3, Imaginary frequency = 322.36i

Y	-0.204005000	-0.445884000	0.159033000
C	-2.390018000	1.058961000	0.277196000
C	-2.860958000	-0.298605000	0.161043000
C	-2.515870000	-1.009527000	1.343528000
C	-1.855255000	-0.100200000	2.226242000
C	-1.793871000	1.166065000	1.584681000
C	-3.806217000	-0.824787000	-0.883512000
H	-4.816460000	-0.432797000	-0.704509000
H	-3.534323000	-0.535844000	-1.900730000
H	-3.891793000	-1.915169000	-0.846979000
C	-2.933152000	-2.410199000	1.696836000
H	-3.908896000	-2.410842000	2.201424000
H	-3.034158000	-3.055705000	0.817508000
H	-2.225044000	-2.889829000	2.381128000
C	-1.476122000	-0.391024000	3.651070000
H	-2.292441000	-0.115589000	4.332444000
H	-1.277503000	-1.455334000	3.815168000
H	-0.591695000	0.167808000	3.977332000

C	-1.365312000	2.436091000	2.269143000
H	-2.223945000	3.107254000	2.395279000
H	-0.973855000	2.237799000	3.271266000
H	-0.600151000	2.998895000	1.725056000
C	1.565031000	-2.175133000	2.547869000
O	0.764788000	-2.368763000	1.341594000
C	0.712389000	-3.794269000	1.033839000
C	1.569185000	-4.491032000	2.086755000
C	1.519815000	-3.512568000	3.260630000
H	2.583188000	-1.909756000	2.243369000
H	1.125912000	-1.348923000	3.106528000
H	-0.336731000	-4.103020000	1.088992000
H	1.090461000	-3.935905000	0.018284000
H	1.182101000	-5.483208000	2.333655000
H	2.598017000	-4.607526000	1.729273000
H	0.585271000	-3.620454000	3.822969000
H	2.354343000	-3.635050000	3.956520000
Si	-2.959622000	2.548828000	-0.759513000
C	-4.521364000	3.232990000	0.055523000
H	-4.337202000	3.560570000	1.084936000
H	-4.904731000	4.095999000	-0.502528000
H	-5.316530000	2.478885000	0.088210000
C	-3.372570000	2.104215000	-2.553256000
H	-4.269645000	1.484292000	-2.648070000
H	-3.569461000	3.037212000	-3.096485000
H	-2.548767000	1.601616000	-3.073358000
C	-1.655565000	3.917712000	-0.856168000
H	-2.041625000	4.717516000	-1.500876000
H	-1.422074000	4.372637000	0.111219000
H	-0.719522000	3.568660000	-1.308352000
N	-0.475454000	-1.638996000	-2.204602000
C	1.636394000	-0.396465000	-2.069895000
C	0.978112000	-1.647264000	-2.245662000
C	1.707385000	-2.811766000	-2.513014000
C	3.095082000	-2.777707000	-2.613807000
H	3.645911000	-3.689945000	-2.823385000
C	3.757780000	-1.557838000	-2.487248000
H	4.836543000	-1.506161000	-2.605263000
C	3.039056000	-0.398634000	-2.226238000
H	3.566581000	0.546685000	-2.167842000
C	-1.101271000	-2.957369000	-2.034264000
H	-0.928204000	-3.616633000	-2.896984000
H	-2.178879000	-2.817955000	-1.932912000
H	-0.724567000	-3.456026000	-1.139486000
C	-1.008336000	-1.056843000	-3.460801000
H	-0.563240000	-0.080772000	-3.654503000
H	-2.092075000	-0.949822000	-3.383637000
H	-0.772927000	-1.715972000	-4.307921000
H	1.190598000	-3.752092000	-2.676005000
C	0.929421000	0.852585000	-1.694477000
H	-0.132833000	0.896130000	-1.978741000
H	1.387364000	1.736961000	-2.125720000
C	1.500241000	0.865041000	1.325697000
H	2.437824000	0.383035000	1.605013000
H	0.885478000	1.171971000	2.171702000
C	1.565081000	1.751682000	0.219168000
H	0.774152000	2.499649000	0.144876000
C	2.916944000	2.273065000	-0.214790000

C	3.405627000	3.348570000	0.772256000
H	2.892129000	2.712510000	-1.219550000
H	3.643707000	1.451315000	-0.228343000
C	4.763972000	3.932357000	0.376062000
H	2.664206000	4.158943000	0.826512000
H	3.467189000	2.916945000	1.779503000
C	5.255834000	4.998087000	1.352836000
H	5.503029000	3.120796000	0.314415000
H	4.695703000	4.362207000	-0.633185000
H	6.228289000	5.397128000	1.045460000
H	4.554753000	5.839267000	1.410114000
H	5.368508000	4.589436000	2.364157000

E-sp^3

Y	-0.400738000	0.527386000	-0.180257000
C	-2.440013000	-1.142091000	-0.314963000
C	-2.999937000	0.173412000	-0.498991000
C	-2.531717000	0.703702000	-1.734840000
C	-1.686321000	-0.274553000	-2.341613000
C	-1.631453000	-1.402952000	-1.482609000
C	-4.042427000	0.848673000	0.350881000
H	-5.051952000	0.582292000	0.009824000
H	-3.981823000	0.560442000	1.402924000
H	-3.977179000	1.941780000	0.294827000
C	-2.988760000	1.982797000	-2.377902000
H	-3.868901000	1.804731000	-3.010359000
H	-3.281391000	2.741274000	-1.643162000
H	-2.220158000	2.420803000	-3.023781000
C	-1.076855000	-0.198799000	-3.712307000
H	-1.700360000	-0.732542000	-4.442397000
H	-0.986623000	0.831895000	-4.072023000
H	-0.083503000	-0.660241000	-3.748641000
C	-0.978127000	-2.701670000	-1.862193000
H	-1.617850000	-3.261908000	-2.557340000
H	-0.017771000	-2.546723000	-2.363983000
H	-0.795938000	-3.346240000	-0.999413000
C	1.317494000	2.493268000	-2.272758000
O	0.349848000	2.572798000	-1.176313000
C	-0.029929000	3.967189000	-0.968485000
C	0.790747000	4.779652000	-1.966827000
C	1.091000000	3.762534000	-3.070346000
H	2.320819000	2.450735000	-1.836267000
H	1.121808000	1.568762000	-2.819029000
H	-1.105640000	4.046200000	-1.152062000
H	0.189185000	4.228078000	0.070812000
H	0.242024000	5.654386000	-2.325533000
H	1.721038000	5.128002000	-1.505610000
H	0.233907000	3.649090000	-3.743608000
H	1.964771000	4.030361000	-3.670592000
Si	-3.040538000	-2.528386000	0.844744000
C	-3.925200000	-3.815820000	-0.217247000
H	-3.256577000	-4.307874000	-0.931195000
H	-4.358465000	-4.597229000	0.419375000
H	-4.743439000	-3.360777000	-0.787675000
C	-4.283386000	-1.944395000	2.148036000
H	-5.190433000	-1.517922000	1.707309000
H	-4.591322000	-2.823955000	2.728001000
H	-3.879997000	-1.219501000	2.864791000

C	-1.632666000	-3.376862000	1.792074000
H	-2.033094000	-4.263457000	2.300305000
H	-0.813300000	-3.718127000	1.150409000
H	-1.204845000	-2.732905000	2.569585000
N	-0.401551000	1.081254000	2.315942000
C	1.957801000	0.321437000	2.114711000
C	1.032421000	1.362595000	2.380438000
C	1.491578000	2.644864000	2.718606000
C	2.851907000	2.915226000	2.817934000
H	3.184050000	3.912529000	3.091986000
C	3.771923000	1.892692000	2.603786000
H	4.836787000	2.078743000	2.709716000
C	3.319894000	0.622709000	2.265374000
H	4.042206000	-0.173447000	2.122515000
C	-1.263351000	2.276421000	2.303872000
H	-1.213823000	2.838387000	3.246623000
H	-2.297062000	1.956308000	2.156630000
H	-0.983237000	2.956843000	1.492488000
C	-0.840563000	0.237159000	3.454993000
H	-0.198158000	-0.635003000	3.562401000
H	-1.867037000	-0.094905000	3.282804000
H	-0.797159000	0.814711000	4.388394000
H	0.788718000	3.440200000	2.940494000
C	1.563914000	-1.090869000	1.714686000
H	0.469088000	-1.203245000	1.742613000
H	1.924445000	-1.786153000	2.486958000
C	1.598727000	-0.583539000	-0.772108000
H	2.349102000	0.232984000	-0.784076000
H	1.653281000	-1.049918000	-1.764740000
C	2.021308000	-1.589053000	0.310912000
H	1.435787000	-2.509943000	0.163424000
C	3.501093000	-2.014443000	0.250707000
C	3.838272000	-2.890716000	-0.957212000
H	3.765483000	-2.566226000	1.167356000
H	4.142569000	-1.122451000	0.221685000
C	5.307193000	-3.316474000	-1.002814000
H	3.201750000	-3.788548000	-0.944101000
H	3.591624000	-2.352378000	-1.882974000
C	5.640132000	-4.190310000	-2.210003000
H	5.943555000	-2.419779000	-1.012588000
H	5.557637000	-3.856609000	-0.078736000
H	6.696983000	-4.478311000	-2.214357000
H	5.045201000	-5.111697000	-2.209793000
H	5.435403000	-3.664036000	-3.150446000

Styrene

C	-1.778484000	-1.044907000	0.000001000
C	-0.405798000	-1.279708000	-0.000006000
C	0.514778000	-0.220054000	-0.000001000
C	0.009215000	1.090960000	-0.000004000
C	-1.360341000	1.327946000	-0.000005000
C	-2.262327000	0.261658000	0.000008000
H	-2.469858000	-1.883928000	0.000010000
H	-0.034159000	-2.302623000	-0.000006000
H	0.694213000	1.934501000	-0.000006000
H	-1.728487000	2.351026000	0.000001000
H	-3.332761000	0.450465000	0.000010000
C	1.951264000	-0.529107000	0.000001000
H	2.183481000	-1.594785000	-0.000004000

C	2.973490000	0.334905000	0.000004000
H	2.836249000	1.413291000	0.000006000
H	4.000535000	-0.018101000	0.000006000

D-st

Y	0.241058000	0.438963000	0.116975000
C	2.250512000	-1.306884000	0.471514000
C	2.459999000	-0.278646000	1.460824000
C	1.399998000	-0.326006000	2.408943000
C	0.528006000	-1.394631000	2.041156000
C	1.050701000	-2.001947000	0.868616000
C	3.706002000	0.539016000	1.659660000
H	4.461732000	-0.053118000	2.193062000
H	4.165273000	0.861912000	0.723116000
H	3.520868000	1.425796000	2.273824000
C	1.309022000	0.466364000	3.681931000
H	1.855903000	-0.035143000	4.491774000
H	1.739300000	1.469546000	3.586692000
H	0.272930000	0.577176000	4.017927000
C	-0.613236000	-1.922873000	2.862900000
H	-0.258014000	-2.685420000	3.569815000
H	-1.090914000	-1.139700000	3.460561000
H	-1.384532000	-2.401067000	2.248427000
C	0.540851000	-3.307490000	0.324585000
H	0.870244000	-4.134589000	0.967844000
H	-0.552982000	-3.352172000	0.291480000
H	0.917552000	-3.519315000	-0.679317000
C	-2.728684000	0.842307000	1.732095000
O	-1.345111000	1.280937000	1.717011000
C	-1.281786000	2.669472000	2.169780000
C	-2.699122000	3.033041000	2.616090000
C	-3.364259000	1.669957000	2.833007000
H	-3.179936000	1.059019000	0.756003000
H	-2.729240000	-0.234963000	1.910388000
H	-0.557561000	2.697156000	2.988550000
H	-0.935472000	3.286682000	1.335303000
H	-2.694463000	3.653506000	3.515985000
H	-3.215969000	3.587109000	1.825751000
H	-3.108754000	1.256954000	3.815056000
H	-4.453655000	1.709188000	2.747742000
Si	3.523022000	-2.046661000	-0.739861000
C	4.008127000	-3.758655000	-0.101069000
H	3.172979000	-4.466263000	-0.084891000
H	4.791160000	-4.189663000	-0.737105000
H	4.408999000	-3.694830000	0.917584000
C	5.129617000	-1.056320000	-0.875330000
H	5.654283000	-0.963710000	0.080764000
H	5.793441000	-1.608005000	-1.553481000
H	5.010531000	-0.053433000	-1.299870000
C	2.846347000	-2.211538000	-2.504178000
H	3.643586000	-2.596762000	-3.152530000
H	2.007814000	-2.911452000	-2.587618000
H	2.528450000	-1.250548000	-2.926625000
N	1.580185000	2.378688000	-0.808946000
C	-0.784105000	2.137652000	-1.254062000
C	0.353663000	2.936155000	-1.421748000
C	0.372746000	4.155437000	-2.107823000
C	-0.818901000	4.622575000	-2.654968000
H	-0.834821000	5.565058000	-3.195783000

C	-1.991416000	3.876294000	-2.506329000
H	-2.921867000	4.245922000	-2.931514000
C	-1.964344000	2.662768000	-1.819610000
H	-2.907406000	2.119764000	-1.727662000
C	2.194114000	3.327209000	0.139086000
H	2.510013000	4.251675000	-0.361898000
H	3.069352000	2.871598000	0.604911000
H	1.471393000	3.593102000	0.915951000
C	2.561716000	1.995798000	-1.841873000
H	2.088698000	1.332096000	-2.571701000
H	3.402808000	1.475433000	-1.377002000
H	2.942523000	2.873191000	-2.382895000
C	-0.711917000	-1.179558000	-2.062187000
H	-0.243722000	-2.155362000	-2.002623000
H	-0.140898000	-0.379761000	-2.537698000
C	-2.030765000	-0.983165000	-1.824817000
H	-2.411352000	0.025214000	-1.982402000
H	1.284177000	4.738035000	-2.225123000
C	-3.029636000	-1.967269000	-1.408888000
C	-4.298708000	-1.508694000	-1.013524000
C	-2.792523000	-3.354255000	-1.422680000
C	-5.288269000	-2.399553000	-0.609468000
H	-4.511245000	-0.441805000	-1.037682000
C	-3.781557000	-4.243986000	-1.022027000
H	-1.841964000	-3.739589000	-1.779249000
C	-5.028760000	-3.769747000	-0.606664000
H	-6.264367000	-2.027902000	-0.310682000
H	-3.588051000	-5.312652000	-1.047679000
H	-5.801391000	-4.469485000	-0.300577000

TS2-st, Imaginary frequency = 249.20i

Y	-0.277155000	0.207553000	-0.020214000
C	-2.872517000	-0.066336000	-0.552761000
C	-2.711997000	1.313288000	-0.154860000
C	-1.939516000	1.988151000	-1.137710000
C	-1.615023000	1.050695000	-2.163999000
C	-2.192398000	-0.199750000	-1.817597000
C	-3.417460000	2.036844000	0.961312000
H	-4.303657000	2.557564000	0.573471000
H	-3.763391000	1.363864000	1.745345000
H	-2.788828000	2.802992000	1.430591000
C	-1.683622000	3.466960000	-1.191372000
H	-2.458420000	3.972913000	-1.783433000
H	-1.699737000	3.926148000	-0.197101000
H	-0.720536000	3.706556000	-1.654636000
C	-0.964244000	1.373703000	-3.480620000
H	-1.719584000	1.453184000	-4.274076000
H	-0.436928000	2.332430000	-3.453156000
H	-0.253658000	0.604483000	-3.808544000
C	-2.241605000	-1.369934000	-2.759753000
H	-3.113215000	-1.289441000	-3.423146000
H	-1.359441000	-1.411765000	-3.407969000
H	-2.313666000	-2.329787000	-2.244122000
C	2.220164000	2.040080000	-1.666339000
O	1.263035000	1.998385000	-0.573904000
C	1.582564000	3.038062000	0.399008000
C	2.858889000	3.701627000	-0.108448000
C	2.801431000	3.441953000	-1.616064000

H	2.986525000	1.276214000	-1.491074000
H	1.677293000	1.810467000	-2.585644000
H	0.736128000	3.731754000	0.420702000
H	1.705573000	2.566481000	1.378309000
H	2.892752000	4.765053000	0.143097000
H	3.738550000	3.218523000	0.329408000
H	2.132888000	4.154831000	-2.111581000
H	3.781296000	3.498757000	-2.098029000
Si	-4.170130000	-1.323897000	0.046163000
C	-5.578630000	-1.329219000	-1.212815000
H	-5.247980000	-1.656823000	-2.204406000
H	-6.377871000	-2.008201000	-0.891063000
H	-6.014710000	-0.329355000	-1.321943000
C	-4.931619000	-0.913283000	1.732999000
H	-5.520964000	0.009393000	1.722899000
H	-5.620601000	-1.728026000	1.990653000
H	-4.204696000	-0.846683000	2.551478000
C	-3.501658000	-3.092744000	0.206608000
H	-4.311384000	-3.731384000	0.582540000
H	-3.172778000	-3.527753000	-0.742564000
H	-2.673004000	-3.177061000	0.919035000
N	-0.436917000	-0.315348000	2.440751000
C	1.753969000	-0.367229000	1.350554000
C	0.997600000	-0.039633000	2.497556000
C	1.576198000	0.584811000	3.613209000
C	2.932410000	0.893098000	3.603989000
H	3.384938000	1.362103000	4.473366000
C	3.702485000	0.594757000	2.478552000
H	4.761561000	0.840335000	2.463195000
C	3.114943000	-0.029691000	1.380028000
H	3.742296000	-0.267300000	0.524557000
C	-1.255911000	0.589976000	3.263804000
H	-1.101999000	0.424177000	4.338551000
H	-2.307473000	0.406553000	3.041703000
H	-1.020691000	1.632080000	3.031169000
C	-0.747526000	-1.709630000	2.826354000
H	-0.185489000	-2.414853000	2.214199000
H	-1.815674000	-1.891448000	2.684024000
H	-0.491943000	-1.882801000	3.880976000
C	0.234901000	-2.042497000	-0.708456000
H	0.312423000	-2.126271000	-1.790632000
H	-0.630180000	-2.569503000	-0.300210000
C	1.438805000	-2.098681000	0.047480000
H	1.415431000	-2.649581000	0.984721000
H	0.983311000	0.824711000	4.490758000
C	2.752860000	-2.200488000	-0.630160000
C	3.784911000	-2.918241000	-0.008035000
C	2.990477000	-1.660216000	-1.903249000
C	5.011409000	-3.103379000	-0.641094000
H	3.620084000	-3.341275000	0.980521000
C	4.220327000	-1.832629000	-2.532808000
H	2.203623000	-1.115763000	-2.420229000
C	5.234655000	-2.556649000	-1.904178000
H	5.792129000	-3.675571000	-0.147805000
H	4.384430000	-1.414265000	-3.522399000
H	6.190608000	-2.698957000	-2.400246000

E-st

Y	-0.613240000	0.497326000	-0.090666000
C	-2.559652000	-1.244065000	-0.205661000
C	-3.172941000	-0.016043000	0.238457000
C	-3.121584000	0.931565000	-0.821462000
C	-2.491773000	0.308054000	-1.941828000
C	-2.156642000	-1.021383000	-1.575510000
C	-3.898925000	0.228940000	1.534048000
H	-4.968430000	0.002545000	1.426015000
H	-3.527209000	-0.392779000	2.350440000
H	-3.835559000	1.275876000	1.853860000
C	-3.769288000	2.287773000	-0.829423000
H	-4.780335000	2.230480000	-1.254569000
H	-3.877560000	2.704715000	0.177743000
H	-3.213939000	3.012795000	-1.434992000
C	-2.320980000	0.920557000	-3.303443000
H	-3.161607000	0.656172000	-3.959001000
H	-2.289037000	2.015016000	-3.260944000
H	-1.407861000	0.577153000	-3.801893000
C	-1.634520000	-2.051791000	-2.535463000
H	-2.462118000	-2.638180000	-2.956373000
H	-1.109875000	-1.591319000	-3.377773000
H	-0.941991000	-2.753691000	-2.064903000
C	0.424362000	3.322149000	-1.561083000
O	-0.277157000	2.833829000	-0.371891000
C	-0.631522000	3.958400000	0.488004000
C	0.008780000	5.183400000	-0.154368000
C	0.068557000	4.795641000	-1.633987000
H	1.496254000	3.158775000	-1.409785000
H	0.081015000	2.729407000	-2.411837000

H	-1.723527000	4.023175000	0.512467000
H	-0.248217000	3.750045000	1.490979000
H	-0.574380000	6.089679000	0.028948000
H	1.017953000	5.340392000	0.241133000
H	-0.906103000	4.935032000	-2.114887000
H	0.811967000	5.368157000	-2.195177000
Si	-2.708052000	-2.977165000	0.564039000
C	-4.078682000	-3.903159000	-0.347091000
H	-3.855617000	-4.026207000	-1.412500000
H	-4.213787000	-4.904831000	0.079343000
H	-5.036107000	-3.375288000	-0.267199000
C	-3.176889000	-2.951200000	2.400922000
H	-4.187968000	-2.566623000	2.570589000
H	-3.163572000	-3.986995000	2.763528000
H	-2.487814000	-2.383840000	3.038428000
C	-1.103146000	-3.980839000	0.429647000
H	-1.188517000	-4.868505000	1.069411000
H	-0.911706000	-4.340666000	-0.586506000
H	-0.213305000	-3.431106000	0.758433000
N	0.429232000	-0.030646000	2.162852000
C	2.386933000	0.360579000	0.668048000
C	1.627727000	0.732964000	1.807989000
C	2.007360000	1.849038000	2.571157000
C	3.113479000	2.615138000	2.221078000
H	3.396846000	3.464304000	2.836630000
C	3.860283000	2.269752000	1.096666000
H	4.733594000	2.851780000	0.815645000
C	3.495875000	1.158665000	0.345949000
H	4.096190000	0.870013000	-0.512472000

C	-0.499704000	0.672657000	3.067048000
H	-0.083444000	0.814153000	4.073707000
H	-1.409581000	0.075722000	3.161977000
H	-0.764972000	1.660725000	2.671473000
C	0.756257000	-1.335030000	2.787594000
H	1.482886000	-1.880375000	2.188137000
H	-0.154237000	-1.933009000	2.871520000
H	1.180885000	-1.176654000	3.788145000
C	1.160341000	-0.285413000	-1.425501000
H	1.701609000	0.487553000	-1.993263000
H	0.936056000	-1.104160000	-2.118991000
C	2.051264000	-0.806973000	-0.275541000
H	1.453189000	-1.543192000	0.284452000
H	1.454253000	2.116679000	3.465138000
C	3.318554000	-1.573124000	-0.676310000
C	4.065053000	-2.239475000	0.306292000
C	3.754600000	-1.642425000	-2.000951000
C	5.210998000	-2.955757000	-0.025068000
H	3.753407000	-2.190703000	1.348829000
C	4.906505000	-2.357078000	-2.337434000
H	3.187141000	-1.141331000	-2.779688000
C	5.637017000	-3.016351000	-1.352993000
H	5.772432000	-3.467716000	0.752169000
H	5.227628000	-2.399701000	-3.375007000
H	6.530583000	-3.575991000	-1.615463000

F-st

Y	0.812595000	-0.236528000	0.005653000
C	3.250268000	-0.358861000	-1.015240000
C	2.742768000	-1.711055000	-1.057593000
C	1.684033000	-1.781201000	-2.004218000
C	1.504118000	-0.476997000	-2.554795000
C	2.465416000	0.389496000	-1.964104000
C	3.360836000	-2.906230000	-0.384696000
H	4.234540000	-3.259061000	-0.947874000
H	3.700799000	-2.689839000	0.632052000
H	2.669770000	-3.753143000	-0.332049000
C	0.987587000	-3.023224000	-2.481378000
H	1.429048000	-3.367556000	-3.426146000
H	1.078195000	-3.852720000	-1.772558000
H	-0.079865000	-2.863439000	-2.663852000
C	0.588180000	-0.119848000	-3.689909000
H	1.138792000	-0.110779000	-4.640435000
H	-0.228501000	-0.839264000	-3.798332000
H	0.141010000	0.873223000	-3.570184000
C	2.696528000	1.797582000	-2.437209000
H	3.407541000	1.805525000	-3.275120000
H	1.773383000	2.258727000	-2.801613000
H	3.102801000	2.446372000	-1.660275000
C	0.445748000	-3.170901000	1.669074000
O	0.952380000	-1.806131000	1.818354000
C	1.682287000	-1.694184000	3.075625000
C	1.412997000	-2.991853000	3.825048000
C	1.216539000	-3.993879000	2.685620000
H	-0.627289000	-3.154940000	1.881473000
H	0.602992000	-3.471407000	0.631661000
H	2.743541000	-1.567614000	2.839187000
H	1.314537000	-0.809779000	3.601854000

H	2.237931000	-3.254668000	4.492616000	H	-4.230091000	4.157911000	-0.409410000
H	0.500931000	-2.907414000	4.425925000	H	-3.299644000	3.869607000	1.072772000
H	2.181432000	-4.311276000	2.276129000	C	-5.263086000	1.752956000	-0.635658000
H	0.662868000	-4.885785000	2.991251000	H	-5.196984000	0.703241000	-0.921933000
Si	4.975570000	0.080343000	-0.354138000	H	-5.316413000	2.357291000	-1.547946000
C	6.250425000	-0.823250000	-1.416444000	H	-6.198734000	1.898372000	-0.069436000
H	6.137407000	-0.557573000	-2.473962000	C	-1.342764000	-1.011231000	-0.714874000
H	7.267844000	-0.549314000	-1.111057000	H	-1.380429000	-2.038308000	-0.299007000
H	6.166470000	-1.912538000	-1.338943000	H	-1.237816000	-1.192585000	-1.794148000
C	5.240984000	-0.425129000	1.458401000	C	-2.751578000	-0.373928000	-0.544844000
H	5.215879000	-1.509138000	1.613498000	H	-2.761857000	0.555285000	-1.131398000
H	6.235124000	-0.083823000	1.774104000	N	0.911604000	1.821410000	1.585958000
H	4.515540000	0.035847000	2.139351000	C	-0.534816000	2.479148000	-0.265278000
C	5.388564000	1.926279000	-0.454933000	C	0.604803000	2.746127000	0.510668000
H	6.380020000	2.072775000	-0.007516000	C	1.393137000	3.861630000	0.203362000
H	5.445976000	2.279639000	-1.489531000	C	1.037939000	4.691043000	-0.859798000
H	4.694117000	2.580826000	0.084382000	H	1.652730000	5.559621000	-1.078917000
N	-4.091151000	2.157663000	0.143954000	C	-0.091295000	4.422421000	-1.632595000
C	-3.089428000	0.029913000	0.882111000	C	-0.876831000	3.313304000	-1.330490000
C	-3.733291000	1.261147000	1.187148000	H	-1.777679000	3.096543000	-1.896154000
C	-3.961792000	1.593422000	2.533329000	C	-0.152501000	1.831701000	2.622626000
C	-3.604823000	0.736170000	3.571556000	H	-0.202922000	2.816993000	3.105393000
H	-3.819110000	1.016878000	4.599639000	H	0.076382000	1.078635000	3.380476000
C	-3.002592000	-0.483906000	3.280056000	H	-1.126647000	1.598744000	2.191723000
H	-2.742230000	-1.176913000	4.076302000	C	2.211726000	2.058563000	2.230069000
C	-2.747471000	-0.811103000	1.948746000	H	2.387023000	1.271407000	2.966874000
H	-2.270975000	-1.759800000	1.719130000	H	2.237255000	3.023671000	2.755523000
C	-4.185403000	3.562342000	0.508922000	H	3.014479000	2.029265000	1.490806000
H	-5.084073000	3.808405000	1.101420000	H	-1.219513000	1.672034000	-0.003662000

H	-0.360310000	5.079536000	-2.454242000
C	-3.801782000	-1.286799000	-1.189011000
C	-3.968757000	-1.241728000	-2.581432000
C	-4.566373000	-2.209758000	-0.466430000
C	-4.854722000	-2.095558000	-3.232510000
H	-3.396864000	-0.518884000	-3.162125000
C	-5.455150000	-3.068412000	-1.115423000
H	-4.484960000	-2.251257000	0.616437000
C	-5.601631000	-3.018560000	-2.499585000
H	-4.968958000	-2.035080000	-4.312052000
H	-6.042085000	-3.773153000	-0.531421000
H	-6.297702000	-3.684256000	-3.002636000
H	-4.452239000	2.531241000	2.774218000
H	2.273346000	4.105111000	0.787504000

TS3-st (C-H activation), Imaginary frequency = 1340.97i

Y	1.005355000	-0.177500000	0.043674000
C	3.364252000	-0.350826000	-1.015251000
C	2.938196000	-1.722714000	-0.843784000
C	1.864429000	-1.986262000	-1.734876000
C	1.612168000	-0.794601000	-2.483178000
C	2.531508000	0.201494000	-2.056567000
C	3.625619000	-2.764126000	-0.003595000
H	4.500864000	-3.167212000	-0.529871000
H	3.982811000	-2.369089000	0.951372000
H	2.974152000	-3.617031000	0.212205000
C	1.193044000	-3.308848000	-1.979960000
H	1.528054000	-3.739470000	-2.932675000
H	1.428757000	-4.043901000	-1.204018000
H	0.101007000	-3.226299000	-2.037346000
C	0.671812000	-0.693436000	-3.649756000
H	1.204961000	-0.900277000	-4.587608000
H	-0.145248000	-1.418639000	-3.583153000
H	0.229092000	0.302622000	-3.747012000
C	2.667791000	1.547319000	-2.712762000
H	3.490622000	1.540034000	-3.440089000
H	1.758533000	1.816159000	-3.256142000
H	2.868799000	2.351538000	-2.001167000
C	0.479758000	-2.894783000	2.072914000
O	1.091184000	-1.567784000	2.034124000
C	1.906849000	-1.373153000	3.227772000
C	1.542816000	-2.519164000	4.159303000
C	1.210462000	-3.642774000	3.175066000
H	-0.582847000	-2.764767000	2.301115000
H	0.586518000	-3.343913000	1.082265000
H	2.959401000	-1.406022000	2.928940000
H	1.674465000	-0.386376000	3.634658000
H	2.363107000	-2.770006000	4.837076000
H	0.665746000	-2.262287000	4.763608000
H	2.126236000	-4.103730000	2.789465000
H	0.590007000	-4.428679000	3.613986000
Si	5.036060000	0.328537000	-0.419402000
C	6.401172000	-0.597548000	-1.339238000
H	6.296483000	-0.477137000	-2.423718000
H	7.387852000	-0.211632000	-1.054897000
H	6.392651000	-1.671637000	-1.123123000
C	5.304553000	0.093413000	1.446978000
H	5.435764000	-0.955492000	1.733969000
H	6.224384000	0.616262000	1.738630000

H	4.493465000	0.517044000	2.051744000
C	5.259763000	2.176720000	-0.755581000
H	6.220391000	2.484858000	-0.323393000
H	5.297935000	2.408291000	-1.824612000
H	4.485802000	2.808411000	-0.304770000
N	-4.293799000	2.129348000	-0.158895000
C	-3.286106000	0.132983000	0.851818000
C	-3.948212000	1.380866000	0.993825000
C	-4.218425000	1.862579000	2.284882000
C	-3.894952000	1.125740000	3.421270000
H	-4.138717000	1.518616000	4.405105000
C	-3.294360000	-0.122934000	3.287976000
H	-3.067341000	-0.724552000	4.164428000
C	-2.990902000	-0.594468000	2.011158000
H	-2.510374000	-1.565038000	1.910368000
C	-4.386640000	3.569424000	0.005859000
H	-5.295534000	3.899168000	0.539991000
H	-4.405065000	4.032818000	-0.986543000
H	-3.509072000	3.944282000	0.540118000
C	-5.438818000	1.608784000	-0.905031000
H	-5.357479000	0.528414000	-1.037066000
H	-5.468939000	2.075815000	-1.895733000
H	-6.393657000	1.820564000	-0.393629000
C	-1.374420000	-0.807426000	-0.555321000
H	-1.236795000	-1.647394000	0.152211000
H	-1.178526000	-1.230753000	-1.549868000
C	-2.866797000	-0.376048000	-0.516531000
H	-2.962116000	0.485853000	-1.189941000
N	0.895827000	1.916777000	1.482835000
C	-0.244991000	1.851900000	-0.653421000
C	0.442512000	2.634216000	0.290026000
C	0.679576000	3.997119000	0.089756000
C	0.187839000	4.604479000	-1.064941000
H	0.359006000	5.665644000	-1.224707000
C	-0.525493000	3.860552000	-2.005850000
C	-0.729371000	2.497492000	-1.799386000
H	-1.277682000	1.925301000	-2.545086000
C	-0.208339000	1.822049000	2.467262000
H	-0.469606000	2.818358000	2.849731000
H	0.101813000	1.193532000	3.307524000
H	-1.096652000	1.386196000	2.005301000
C	2.082004000	2.497483000	2.129301000
H	2.429132000	1.822176000	2.916672000
H	1.862878000	3.466625000	2.598161000
H	2.882902000	2.630356000	1.398961000
H	-0.835747000	0.563311000	-0.516317000
H	-0.913120000	4.344696000	-2.898326000
C	-3.750756000	-1.484055000	-1.095875000
C	-3.766821000	-1.664854000	-2.487613000
C	-4.526222000	-2.345641000	-0.313056000
C	-4.511396000	-2.682739000	-3.076510000
H	-3.193676000	-0.987898000	-3.120144000
C	-5.274378000	-3.368277000	-0.900007000
H	-4.568641000	-2.209361000	0.763580000
C	-5.266840000	-3.545245000	-2.280970000
H	-4.511846000	-2.796125000	-4.157724000
H	-5.873535000	-4.021719000	-0.270770000
H	-5.853495000	-4.338405000	-2.736369000

H	-4.715964000	2.820193000	2.402640000
H	1.229430000	4.591939000	0.813144000

7-st

N	-1.613650000	1.706844000	0.098221000
C	-0.877318000	-0.615101000	0.403046000
C	-1.835621000	0.311822000	-0.076094000
C	-3.008906000	-0.165362000	-0.677563000
C	-3.243360000	-1.529286000	-0.829071000
H	-4.161700000	-1.870429000	-1.300913000
C	-2.295822000	-2.444735000	-0.383166000
H	-2.462368000	-3.513028000	-0.494722000
C	-1.133605000	-1.979590000	0.229472000
H	-0.414240000	-2.702648000	0.605762000
C	-2.796068000	2.533926000	0.239736000
H	-3.335229000	2.713983000	-0.709255000
H	-2.495276000	3.512044000	0.633599000
H	-3.489396000	2.075950000	0.951206000
C	-0.661270000	2.293303000	-0.836980000
H	0.237618000	1.677730000	-0.905701000
H	-0.369415000	3.289412000	-0.483325000
H	-1.084101000	2.398581000	-1.852985000
C	0.593323000	-0.958594000	2.443392000
H	0.896343000	-1.987595000	2.222374000
H	1.396956000	-0.504937000	3.033727000
C	0.352697000	-0.141146000	1.158454000
H	0.130472000	0.887696000	1.469829000
H	-0.312332000	-0.990215000	3.059089000
C	1.640914000	-0.088923000	0.337704000
C	2.672457000	0.764765000	0.755441000
C	1.862365000	-0.883097000	-0.792297000
C	3.886818000	0.817069000	0.076516000
H	2.515963000	1.400002000	1.625831000
C	3.077888000	-0.833025000	-1.476628000
H	1.073171000	-1.538674000	-1.149484000
C	4.095838000	0.013917000	-1.045046000
H	4.669060000	1.490105000	0.419752000
H	3.224876000	-1.458377000	-2.354152000
H	5.041456000	0.053870000	-1.579853000
H	-3.748283000	0.542978000	-1.039823000

G-st

Y	0.366701000	0.549816000	-0.217447000
C	2.231580000	2.122572000	0.764761000
C	1.763474000	2.751032000	-0.445465000
C	0.425021000	3.192909000	-0.252434000
C	0.030736000	2.833432000	1.070643000
C	1.132005000	2.182855000	1.696286000
C	2.578694000	3.075008000	-1.668353000
H	3.011858000	4.081112000	-1.590894000
H	3.407151000	2.377529000	-1.816025000
H	1.972936000	3.070270000	-2.581681000
C	-0.358677000	4.043087000	-1.209075000
H	-0.080584000	5.099297000	-1.092296000
H	-0.171567000	3.781842000	-2.256653000
H	-1.436669000	3.972867000	-1.041481000
C	-1.267385000	3.200252000	1.729868000
H	-1.159795000	4.128920000	2.306563000
H	-2.060497000	3.364943000	0.995273000

H	-1.620775000	2.430615000	2.424825000
C	1.137503000	1.786483000	3.149262000
H	1.504537000	2.611761000	3.774098000
H	0.128938000	1.553855000	3.508951000
H	1.776519000	0.924252000	3.357009000
C	0.525922000	0.309359000	-3.573859000
O	1.155507000	-0.148511000	-2.332207000
C	2.376566000	-0.895364000	-2.643080000
C	2.334083000	-1.115579000	-4.147190000
C	1.590794000	0.127310000	-4.641710000
H	-0.353307000	-0.317526000	-3.752424000
H	0.206041000	1.343337000	-3.424603000
H	3.227765000	-0.279427000	-2.338611000
H	2.362221000	-1.817601000	-2.057842000
H	3.337016000	-1.213331000	-4.571350000
H	1.771280000	-2.024129000	-4.388861000
H	2.260800000	0.993642000	-4.672011000
H	1.151983000	-0.001477000	-5.634782000
Si	4.075895000	1.878755000	1.154307000
C	4.901427000	3.575152000	1.069929000
H	4.432662000	4.274682000	1.771810000
H	5.963192000	3.499309000	1.334949000
H	4.842830000	4.019476000	0.070639000
C	4.953241000	0.720080000	-0.068169000
H	5.025969000	1.140692000	-1.077470000
H	5.982192000	0.555651000	0.276893000
H	4.476745000	-0.264588000	-0.143302000
C	4.404458000	1.193984000	2.888190000
H	5.492427000	1.134052000	3.021259000
H	4.022521000	1.854090000	3.673602000
H	4.006719000	0.189396000	3.072784000
N	-3.429425000	-0.815452000	2.670785000
C	-2.779659000	-1.219189000	0.329950000
C	-2.988045000	-1.698412000	1.654071000
C	-2.697353000	-3.042498000	1.942908000
C	-2.243592000	-3.923193000	0.962416000
H	-2.057897000	-4.963092000	1.218850000
C	-2.059888000	-3.469193000	-0.340641000
H	-1.740205000	-4.149062000	-1.126623000
C	-2.316024000	-2.127437000	-0.633317000
H	-2.174536000	-1.777022000	-1.653057000
C	-3.091053000	-1.177412000	4.037766000
H	-3.696760000	-2.008264000	4.440493000
H	-3.265130000	-0.307225000	4.679778000
H	-2.034099000	-1.451582000	4.110486000
C	-4.834563000	-0.412960000	2.579913000
H	-5.099230000	-0.145297000	1.556950000
H	-5.003361000	0.459144000	3.220753000
H	-5.509028000	-1.221416000	2.909363000
C	-1.885384000	0.816721000	-0.929753000
H	-1.945115000	0.340471000	-1.927770000
H	-2.137502000	1.867317000	-1.127431000
C	-3.021921000	0.234328000	-0.047743000
H	-3.023647000	0.795670000	0.895748000
C	-4.384875000	0.484087000	-0.703486000
C	-4.971513000	1.751819000	-0.571986000
C	-5.055097000	-0.471331000	-1.475575000
C	-6.174774000	2.061215000	-1.199769000

H	-4.477784000	2.505784000	0.039551000
C	-6.261919000	-0.165378000	-2.106760000
H	-4.645834000	-1.472788000	-1.576772000
C	-6.825483000	1.101346000	-1.975626000
H	-6.609778000	3.049967000	-1.076536000
H	-6.765798000	-0.926909000	-2.696916000
H	-7.767005000	1.337166000	-2.464116000
H	-2.854410000	-3.417199000	2.949266000
C	0.584935000	-1.531016000	1.606615000
C	1.904228000	-1.790700000	1.433525000
H	2.616275000	-1.135964000	1.936633000
H	-0.200247000	-2.185482000	1.232048000
H	0.283049000	-0.768911000	2.327148000
C	2.511696000	-2.880479000	0.670391000
C	3.910545000	-3.020875000	0.698521000
C	1.753515000	-3.809850000	-0.067448000
C	4.538060000	-4.053165000	0.006919000
H	4.506259000	-2.323372000	1.282578000
C	2.383076000	-4.836033000	-0.761265000
H	0.669499000	-3.735856000	-0.085214000
C	3.775693000	-4.960146000	-0.727840000
H	5.618864000	-4.153353000	0.047023000
H	1.788422000	-5.552769000	-1.321075000
H	4.261652000	-5.769564000	-1.265561000

TS4-st (styrene insertion), Imaginary frequency = 165.11i

Y	0.953504000	-0.103010000	-0.026887000
C	3.116192000	-1.232977000	-0.990271000
C	2.674317000	-2.071358000	0.098938000
C	1.440316000	-2.680009000	-0.256864000
C	1.096380000	-2.237080000	-1.573380000
C	2.125384000	-1.366785000	-2.028736000
C	3.446260000	-2.397318000	1.348611000
H	4.072375000	-3.285827000	1.193557000
H	4.111355000	-1.585387000	1.653829000
H	2.790255000	-2.625789000	2.195915000
C	0.713492000	-3.737734000	0.527267000
H	1.031375000	-4.737907000	0.202950000
H	0.921044000	-3.676459000	1.600823000
H	-0.373543000	-3.692416000	0.399606000
C	-0.044599000	-2.762379000	-2.397035000
H	0.278571000	-3.621356000	-3.000695000
H	-0.874687000	-3.106211000	-1.772565000
H	-0.440878000	-2.015705000	-3.093344000
C	2.203648000	-0.853420000	-3.439798000
H	2.727338000	-1.575060000	-4.081085000
H	1.209594000	-0.711557000	-3.877800000
H	2.743604000	0.092247000	-3.518294000
C	0.677430000	-0.482089000	3.360988000
O	1.328851000	0.277237000	2.294493000
C	2.304911000	1.201152000	2.871658000
C	1.954289000	1.263832000	4.347911000
C	1.464837000	-0.160614000	4.622168000
H	-0.361714000	-0.141466000	3.423841000
H	0.692552000	-1.539275000	3.083402000
H	3.302337000	0.781945000	2.705536000
H	2.209224000	2.152428000	2.343565000
H	2.814407000	1.546622000	4.960788000

H	1.153319000	1.990783000	4.523748000
H	2.313662000	-0.844520000	4.731957000
H	0.845207000	-0.238797000	5.519489000
Si	4.884276000	-0.538134000	-1.124325000
C	6.087143000	-1.993033000	-1.067463000
H	5.881178000	-2.705119000	-1.875115000
H	7.117379000	-1.637735000	-1.192622000
H	6.041311000	-2.541956000	-0.120893000
C	5.294574000	0.657914000	0.291702000
H	5.324108000	0.172056000	1.273510000
H	6.291879000	1.083053000	0.120586000
H	4.590640000	1.497468000	0.343628000
C	5.210953000	0.400830000	-2.733152000
H	6.255790000	0.736821000	-2.715598000
H	5.091460000	-0.228733000	-3.620847000
H	4.591609000	1.295591000	-2.864750000
N	-4.740760000	0.513111000	-2.221092000
C	-3.632557000	0.877746000	-0.062759000
C	-4.506911000	1.331752000	-1.083098000
C	-5.088726000	2.602790000	-0.964915000
C	-4.848507000	3.414445000	0.139703000
H	-5.321986000	4.390353000	0.205488000
C	-4.018154000	2.962381000	1.160575000
H	-3.835449000	3.576614000	2.038237000
C	-3.417679000	1.709496000	1.043631000
H	-2.753504000	1.378178000	1.839429000
C	-5.036826000	1.209632000	-3.463290000
H	-6.058448000	1.625906000	-3.507604000
H	-4.937808000	0.500718000	-4.292734000
H	-4.321434000	2.022451000	-3.618567000
C	-5.691622000	-0.574443000	-1.990690000
H	-5.456135000	-1.103832000	-1.065495000
H	-5.635618000	-1.287573000	-2.820770000
H	-6.729804000	-0.206622000	-1.920455000
C	-1.402903000	-0.340578000	0.102702000
H	-1.261568000	0.140909000	1.092104000
H	-1.008775000	-1.379736000	0.185645000
C	-2.901276000	-0.449927000	-0.210700000
H	-2.994922000	-0.724265000	-1.269615000
C	-3.507588000	-1.605756000	0.596051000
C	-3.502281000	-2.894868000	0.044018000
C	-4.027411000	-1.442840000	1.885175000
C	-3.982324000	-3.989892000	0.760449000
H	-3.138964000	-3.038133000	-0.972304000
C	-4.508840000	-2.536459000	2.605422000
H	-4.086845000	-0.451394000	2.324465000
C	-4.483161000	-3.814614000	2.050054000
H	-3.979373000	-4.976870000	0.304792000
H	-4.919052000	-2.384186000	3.600623000
H	-4.865449000	-4.663793000	2.609747000
H	-5.751730000	2.958830000	-1.747303000
C	-0.844991000	1.148045000	-1.640446000
C	0.455732000	1.638613000	-1.710127000
H	1.094641000	1.327536000	-2.533934000
H	-1.633012000	1.705282000	-1.147656000
H	-1.195507000	0.470973000	-2.414087000
C	0.993389000	2.650368000	-0.795658000
C	2.329782000	3.091761000	-0.936231000

C	0.238767000	3.181622000	0.277265000
C	2.865375000	4.055353000	-0.086825000
H	2.928945000	2.703623000	-1.756811000
C	0.783326000	4.147975000	1.123131000
H	-0.800980000	2.892085000	0.410138000
C	2.093431000	4.594498000	0.945175000
H	3.884536000	4.399327000	-0.241502000
H	0.168384000	4.570111000	1.914009000
H	2.505667000	5.361189000	1.594933000

H (styrene insertion product)

Y	1.579050000	0.199080000	-0.038967000
C	3.820488000	-0.402765000	-1.138305000
C	3.232987000	-1.666429000	-0.755455000
C	2.077733000	-1.892816000	-1.549027000
C	1.935461000	-0.788666000	-2.451311000
C	3.012613000	0.108755000	-2.218487000
C	3.804155000	-2.660746000	0.219461000
H	4.438318000	-3.388896000	-0.302965000
H	4.425196000	-2.187504000	0.984087000
H	3.027557000	-3.235042000	0.736494000
C	1.238895000	-3.140888000	-1.580142000
H	1.415306000	-3.694406000	-2.511746000
H	1.487711000	-3.819405000	-0.758705000
H	0.161309000	-2.940574000	-1.533052000
C	0.931236000	-0.715824000	-3.565487000
H	1.317173000	-1.216661000	-4.463960000
H	-0.009327000	-1.212057000	-3.303451000
H	0.694155000	0.314069000	-3.848278000
C	3.282920000	1.318637000	-3.068424000
H	3.933745000	1.052193000	-3.912106000
H	2.362800000	1.727494000	-3.498041000
H	3.784234000	2.121293000	-2.523831000
C	0.076467000	-2.168369000	2.006719000
O	0.909567000	-0.961497000	1.907689000
C	1.325735000	-0.536619000	3.245843000
C	0.408640000	-1.289098000	4.194328000
C	0.195152000	-2.612798000	3.455694000
H	-0.946935000	-1.889186000	1.740060000
H	0.457181000	-2.892534000	1.284010000
H	2.375264000	-0.822172000	3.379532000
H	1.226899000	0.551503000	3.296449000
H	0.860164000	-1.416446000	5.181858000
H	-0.539794000	-0.754223000	4.316351000
H	1.058166000	-3.274025000	3.592164000
H	-0.700243000	-3.146571000	3.784184000
Si	5.500788000	0.210753000	-0.486447000
C	6.822901000	-1.062499000	-0.924220000
H	6.876354000	-1.209858000	-2.009331000
H	7.808485000	-0.717688000	-0.588037000
H	6.641562000	-2.040101000	-0.465469000
C	5.436330000	0.447801000	1.398903000
H	5.242167000	-0.475196000	1.956612000
H	6.405022000	0.828512000	1.747202000
H	4.682550000	1.189616000	1.694919000
C	6.031722000	1.872577000	-1.211796000
H	7.013449000	2.124007000	-0.790215000
H	6.147616000	1.843026000	-2.300124000

H	5.356446000	2.699204000	-0.961710000
N	-5.554513000	0.682190000	-2.038194000
C	-4.214192000	0.789868000	0.010869000
C	-5.315567000	1.244674000	-0.755270000
C	-6.125943000	2.267581000	-0.241347000
C	-5.884959000	2.825664000	1.010556000
H	-6.533833000	3.613482000	1.383878000
C	-4.825189000	2.360133000	1.782958000
H	-4.633823000	2.777689000	2.767802000
C	-4.003239000	1.356417000	1.273495000
H	-3.162524000	1.014228000	1.874739000
C	-6.183731000	1.554091000	-3.016179000
H	-7.264147000	1.708062000	-2.846631000
H	-6.069671000	1.105131000	-4.009194000
H	-5.688328000	2.529155000	-3.021788000
C	-6.193432000	-0.631376000	-2.009683000
H	-5.693328000	-1.287184000	-1.293709000
H	-6.126052000	-1.089855000	-3.002824000
H	-7.259556000	-0.569483000	-1.729645000
C	-1.772908000	0.213440000	-0.432290000
H	-1.509436000	0.258177000	0.637035000
H	-1.158965000	-0.592717000	-0.875911000
C	-3.250915000	-0.231511000	-0.567406000
H	-3.466364000	-0.266182000	-1.643765000
C	-3.396712000	-1.654368000	-0.031723000
C	-2.988783000	-2.725427000	-0.842551000
C	-3.883183000	-1.948351000	1.247800000
C	-3.044820000	-4.041479000	-0.388352000
H	-2.648253000	-2.523168000	-1.857390000
C	-3.940779000	-3.266520000	1.707330000
H	-4.252211000	-1.146542000	1.880913000
C	-3.516660000	-4.317218000	0.895943000
H	-2.741822000	-4.853741000	-1.044510000
H	-4.340338000	-3.471231000	2.697734000
H	-3.576284000	-5.343078000	1.249582000
H	-6.966624000	2.626576000	-0.827112000
C	-1.410678000	1.536533000	-1.121819000
C	0.061961000	1.884819000	-0.973646000
H	0.557533000	2.194251000	-1.898187000
H	-2.065410000	2.334915000	-0.744690000
H	-1.646969000	1.441303000	-2.188381000
C	0.563727000	2.568146000	0.193261000
C	1.970945000	2.835102000	0.286623000
C	-0.177695000	2.748098000	1.397349000
C	2.559444000	3.320441000	1.466672000
H	2.564614000	2.826735000	-0.630966000
C	0.422305000	3.222172000	2.557080000
H	-1.249884000	2.572948000	1.385953000
C	1.796360000	3.506413000	2.612626000
H	3.616978000	3.573126000	1.466827000
H	-0.194574000	3.393620000	3.436664000
H	2.245056000	3.891820000	3.522948000

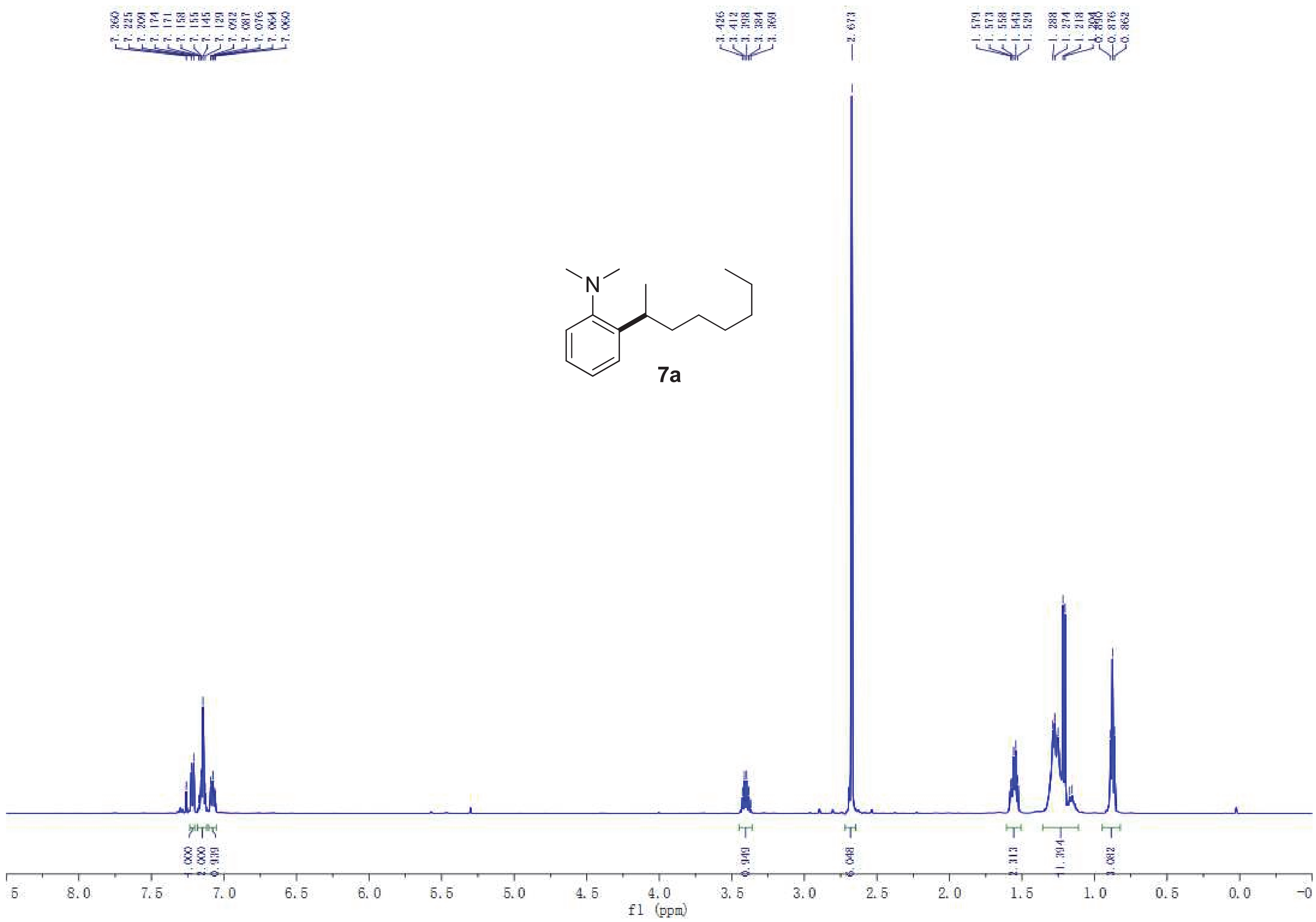
7.260
7.225
7.209
7.174
7.171
7.158
7.155
7.145
7.129
7.092
7.087
7.076
7.064
7.060
3.426
3.412
3.398
3.384
3.369
2.673
1.579
1.573
1.558
1.543
1.529
1.288
1.274
1.218
1.204
0.890
0.876
0.862
7a
1.000
2.000
0.939
0.949
6.048
2.313
1.394
3.082
f1 (ppm)

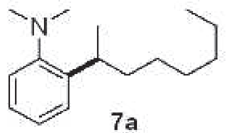

7a

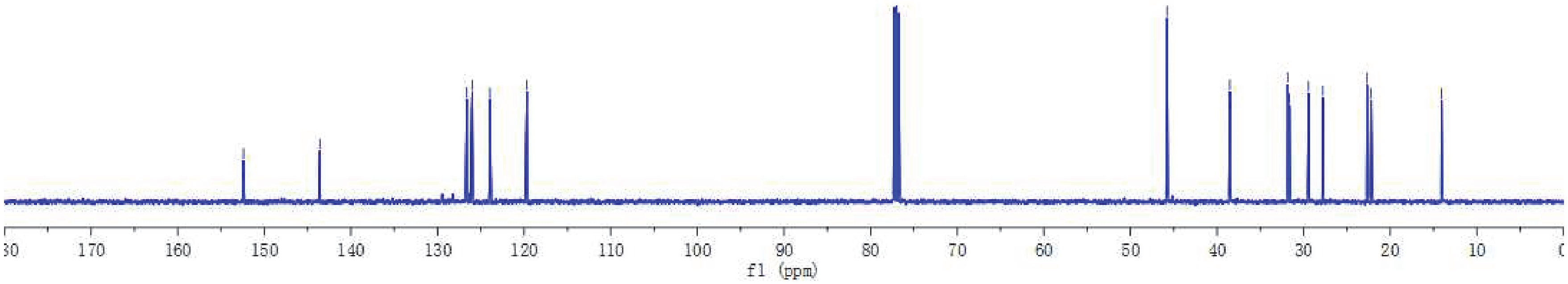

152.399
143.582
126.630
126.006
123.930
119.687
45.762
38.529
31.844
31.629
29.445
27.780
22.686
22.204
14.087
180 170 160 150 140 130 120 110 100 90 80 70 60 50 40 30 20 10 0
f1 (ppm)

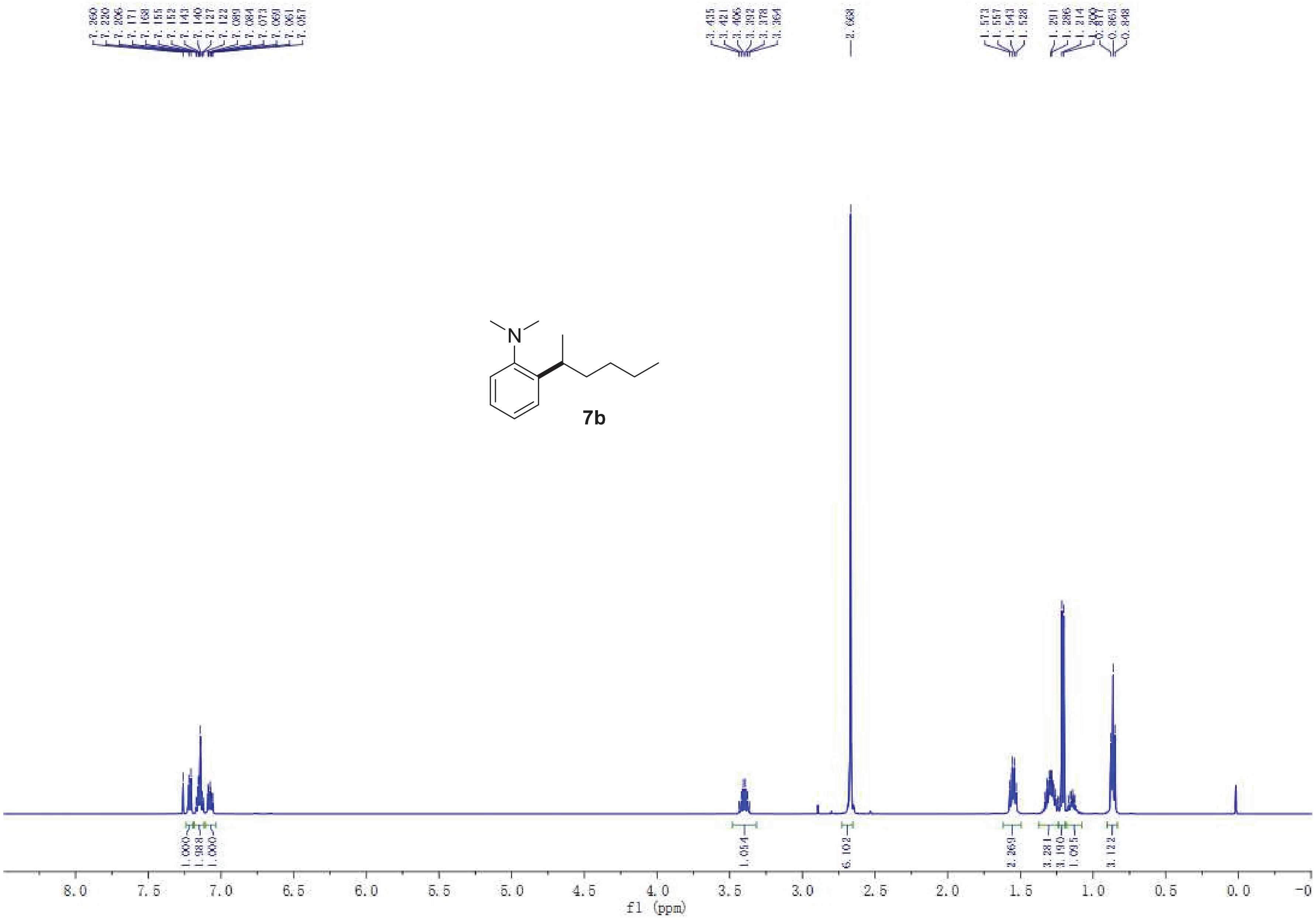
7.260 7.220 7.206 7.171 7.168 7.155 7.152 7.143 7.140 7.127 7.122 7.089 7.084 7.073 7.069 7.061 7.057
3.435 3.421 3.406 3.392 3.378 3.364
2.668
1.573 1.557 1.543 1.528
1.291 1.286 1.214 1.200 0.877 0.863 0.848
7b
1.000 1.988 1.000
1.054
6.102
2.269
3.281 3.190 1.095
3.122
8.0 7.5 7.0 6.5 6.0 5.5 5.0 4.5 4.0 3.5 3.0 2.5 2.0 1.5 1.0 0.5 0.0 -0
f1 (ppm)

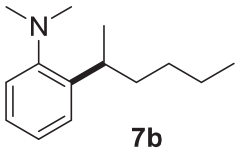

7b

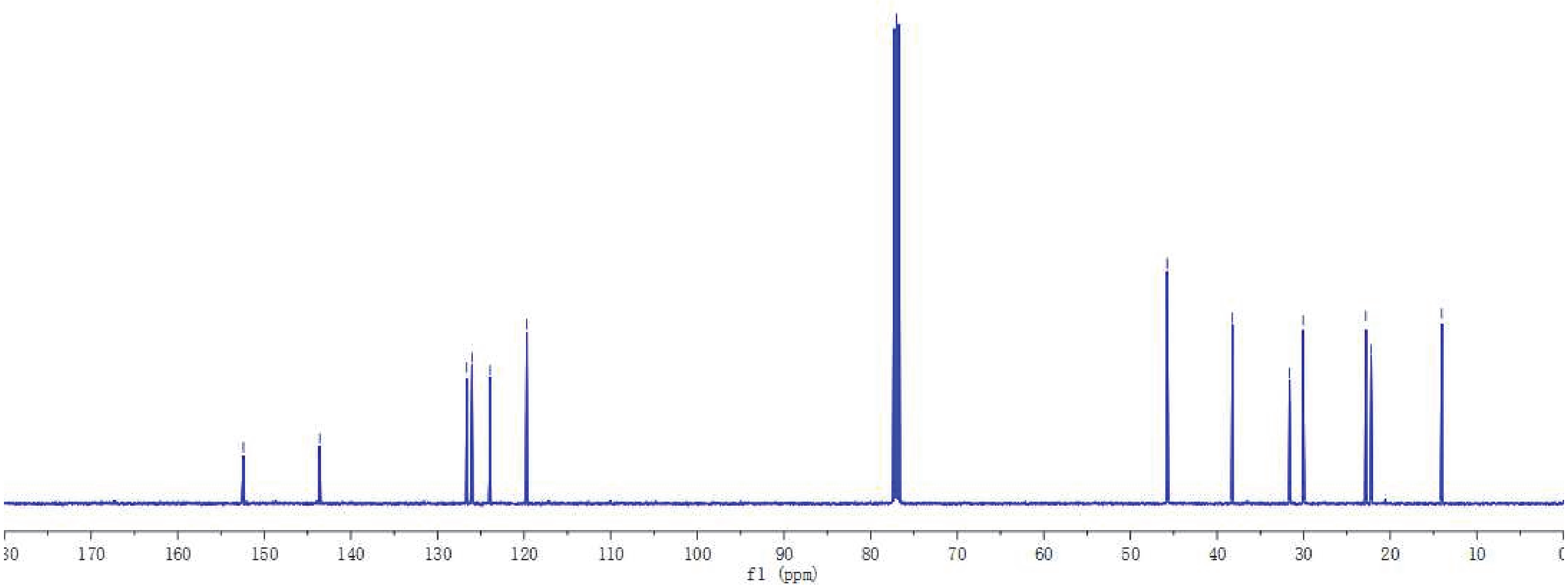

152.411
143.601
126.628
126.012
123.936
119.704
45.765
38.239
31.637
30.055
22.829
22.205
14.051
180 170 160 150 140 130 120 110 100 90 80 70 60 50 40 30 20 10 0
f1 (ppm)

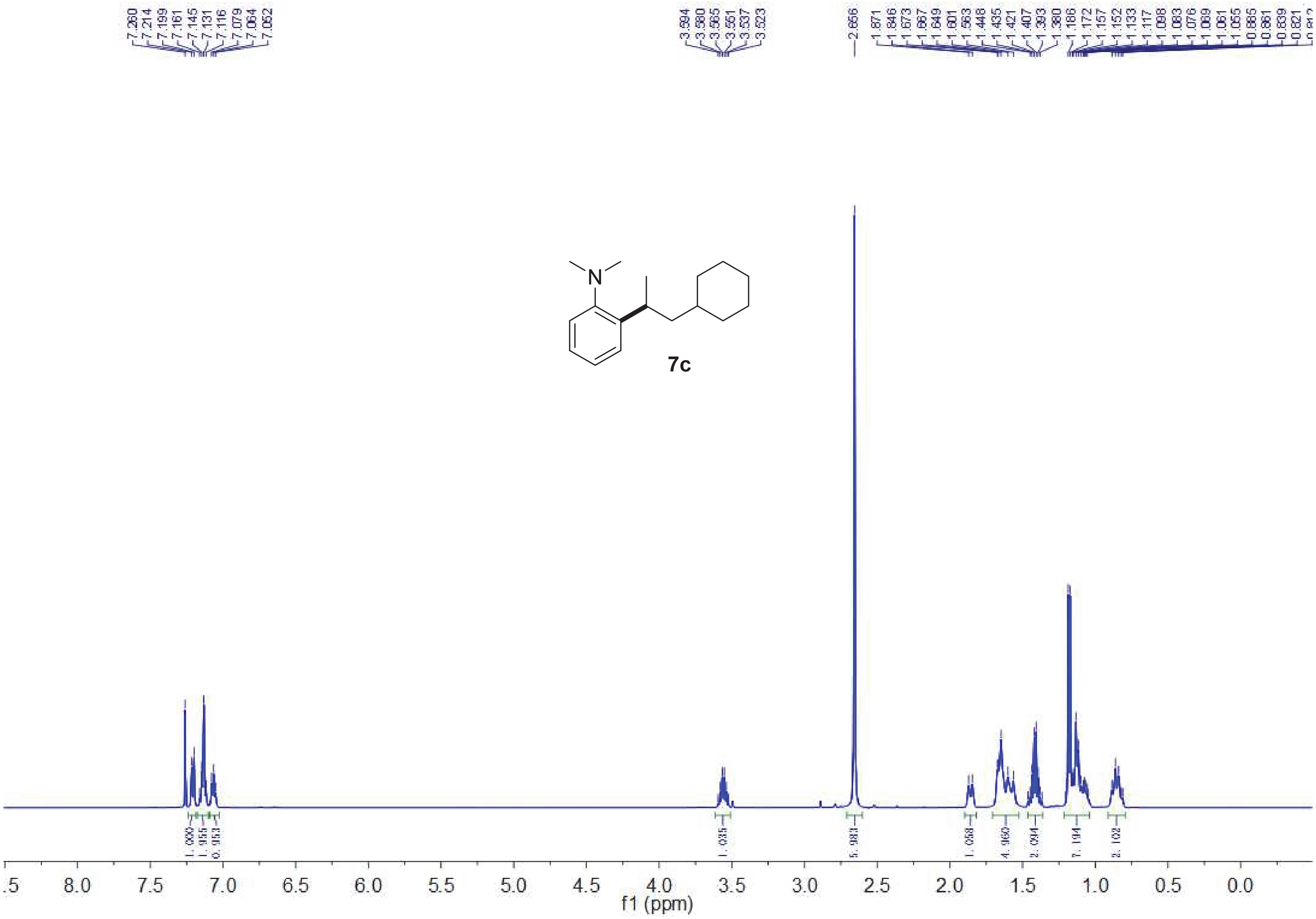
7.260 7.214 7.199 7.161 7.145 7.131 7.116 7.079 7.064 7.052
3.594 3.580 3.565 3.551 3.537 3.523
2.656
1.871 1.846 1.673 1.667 1.649 1.601 1.563 1.448 1.435 1.421 1.407 1.393 1.380 1.186 1.172 1.157 1.152 1.133 1.117 1.098 1.083 1.076 1.069 1.061 1.055 0.885 0.861 0.839 0.821
N
7c
1.000 1.955 0.953 1.035 5.983 1.058 4.960 2.094 7.194 2.102
.5 8.0 7.5 7.0 6.5 6.0 5.5 5.0 4.5 4.0 3.5 3.0 2.5 2.0 1.5 1.0 0.5 0.0
f1 (ppm)

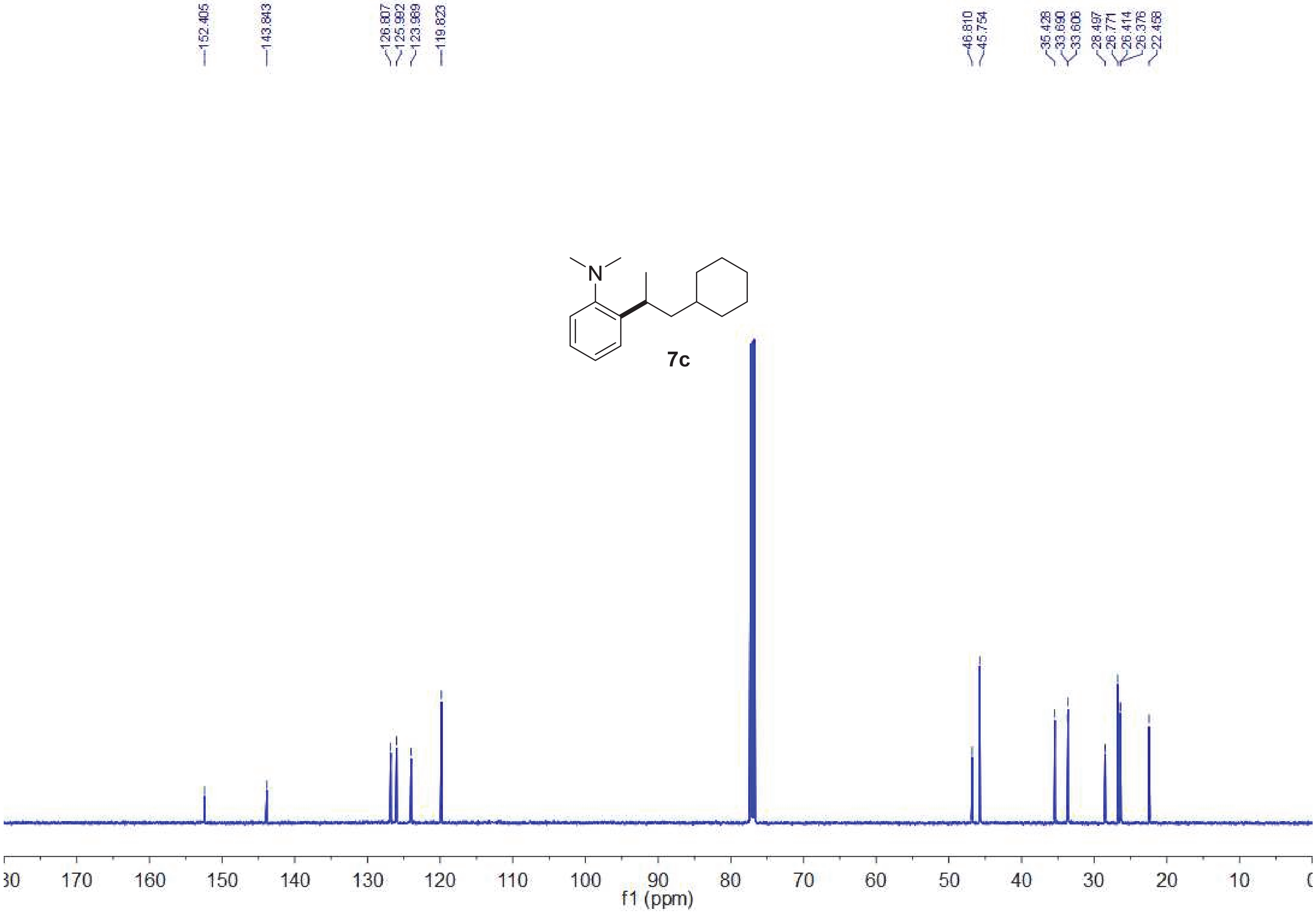
152.405
143.843
126.807
125.992
123.989
119.823
46.810
45.754
35.428
33.690
33.606
28.497
26.771
26.414
26.376
22.458
7c
170
160
150
140
130
120
110
100
90
80
70
60
50
40
30
20
10
f1 (ppm)

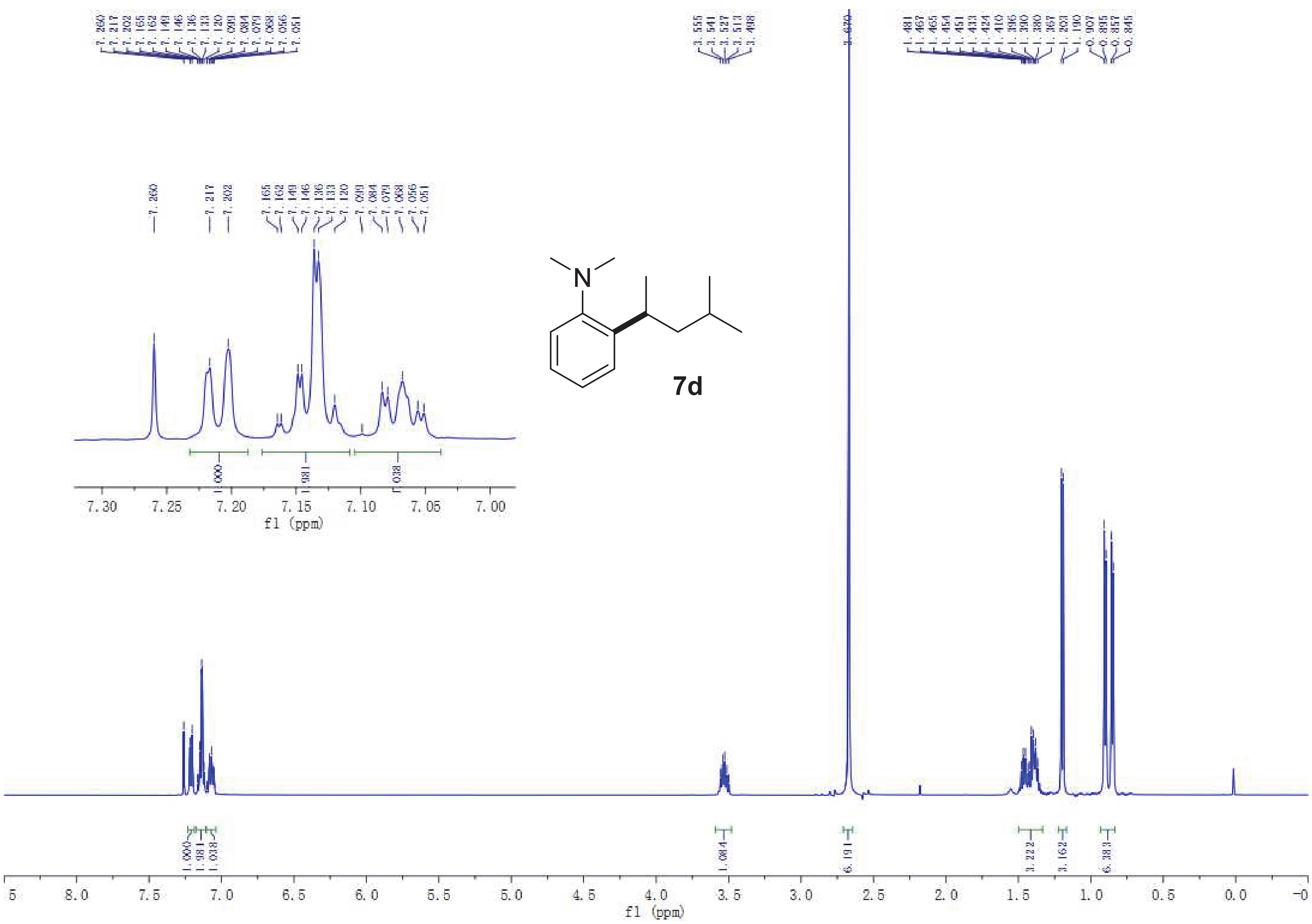
7.260
7.217
7.202
7.165
7.162
7.149
7.146
7.136
7.133
7.120
7.099
7.084
7.079
7.068
7.056
7.051
3.555
3.541
3.527
3.513
3.498
2.670
1.481
1.467
1.465
1.454
1.451
1.433
1.424
1.410
1.396
1.390
1.380
1.367
1.203
1.190
0.907
0.895
0.857
0.845
1.000
1.981
1.038
1.084
6.191
3.222
3.162
6.383
f1 (ppm)
7d

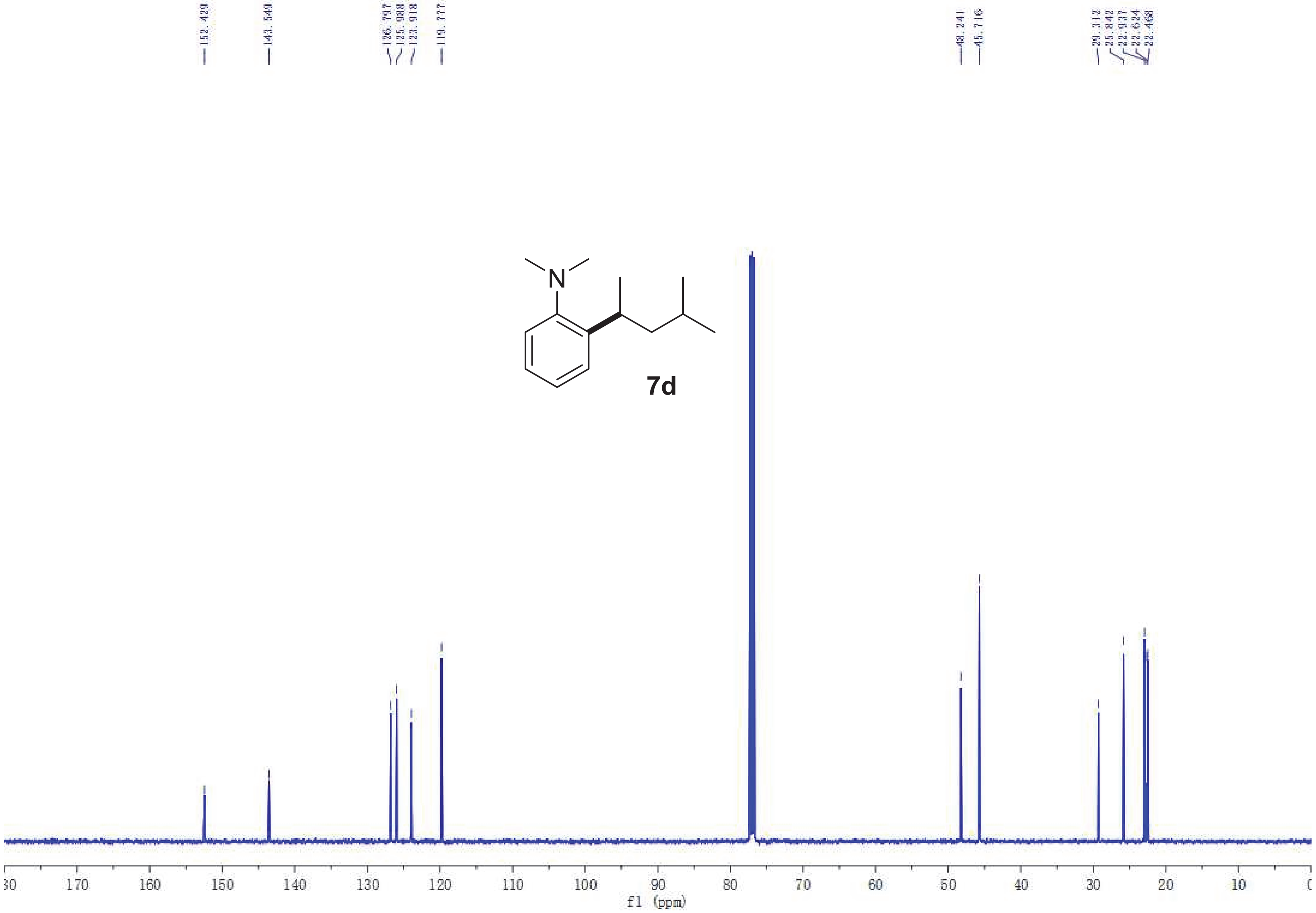
152.429
143.549
126.797
125.988
123.918
119.777
48.241
45.716
29.312
25.842
22.937
22.624
22.468
7d
f1 (ppm)

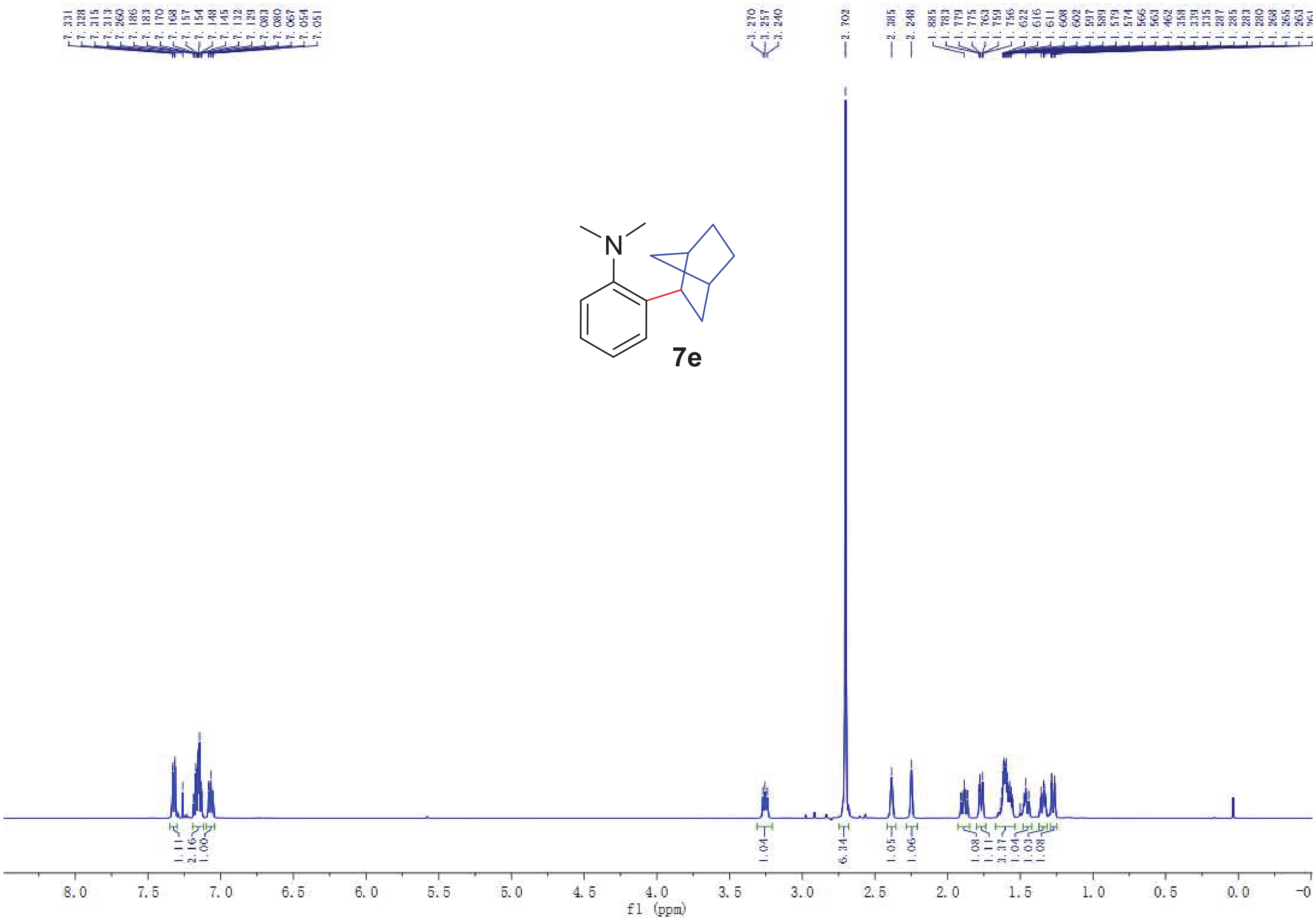
7e
f1 (ppm)

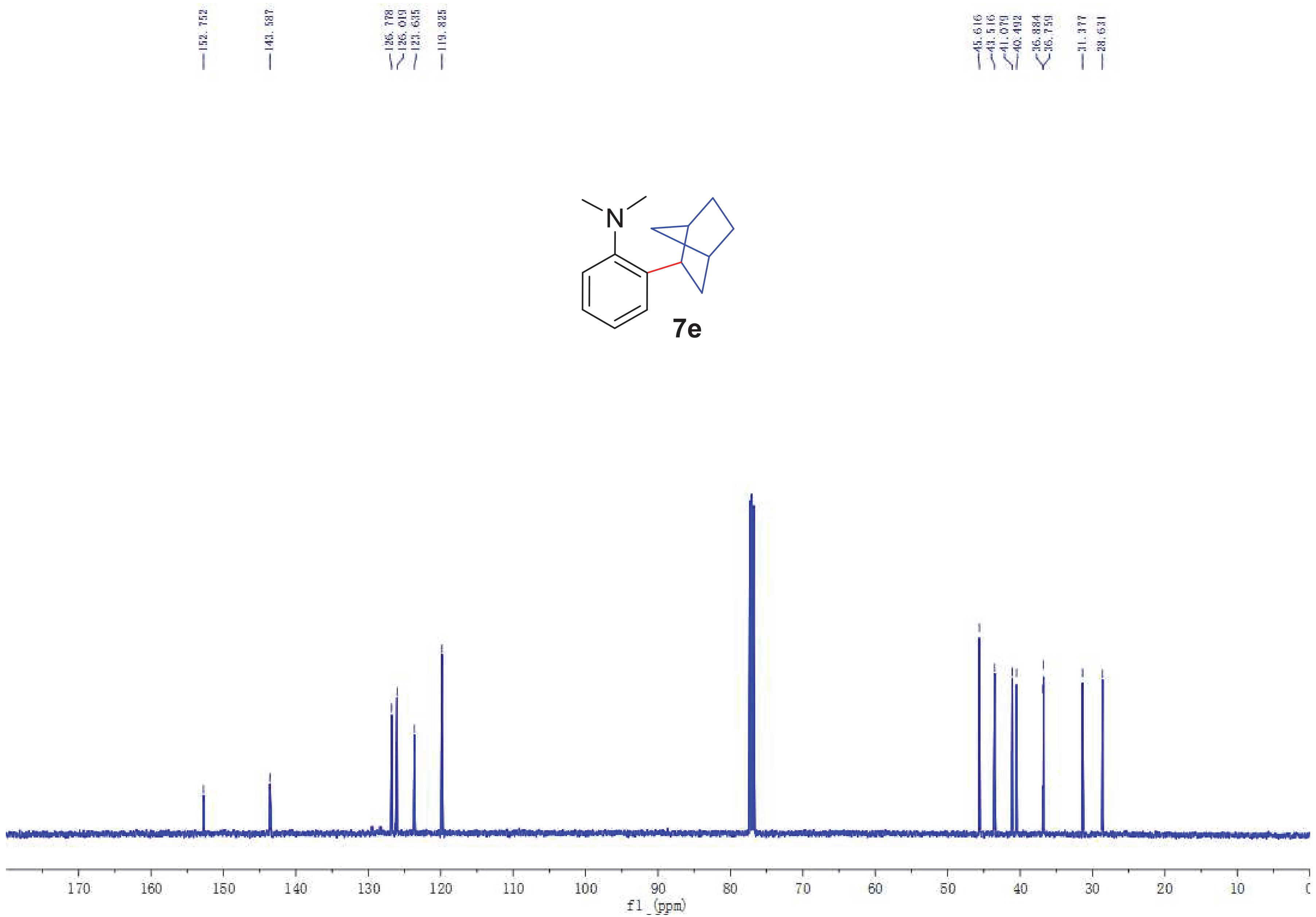
152.752
143.587
126.778
126.019
123.635
119.825
45.616
43.516
41.079
40.492
36.884
36.759
31.377
28.631
N
7e
170 160 150 140 130 120 110 100 90 80 70 60 50 40 30 20 10
f1 (ppm)

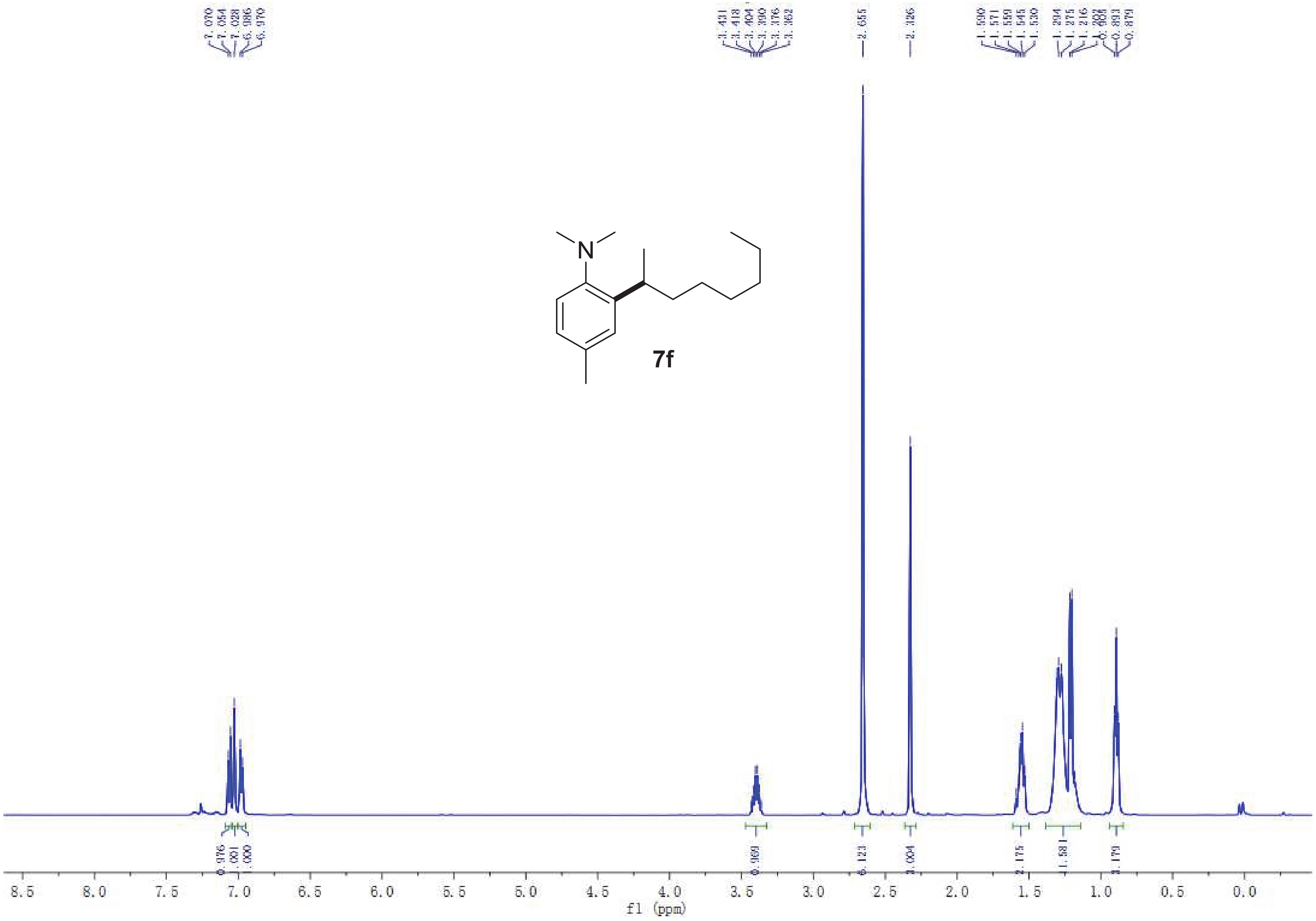
7.070
7.054
7.028
6.986
6.970
3.431
3.418
3.404
3.390
3.376
3.362
2.655
2.326
1.590
1.571
1.559
1.545
1.530
1.294
1.275
1.216
1.202
0.906
0.893
0.879
N
7f
0.976
1.001
1.000
0.969
6.123
3.004
2.175
11.581
3.179
8.5 8.0 7.5 7.0 6.5 6.0 5.5 5.0 4.5 4.0 3.5 3.0 2.5 2.0 1.5 1.0 0.5 0.0
f1 (ppm)

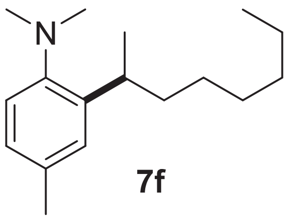

7f

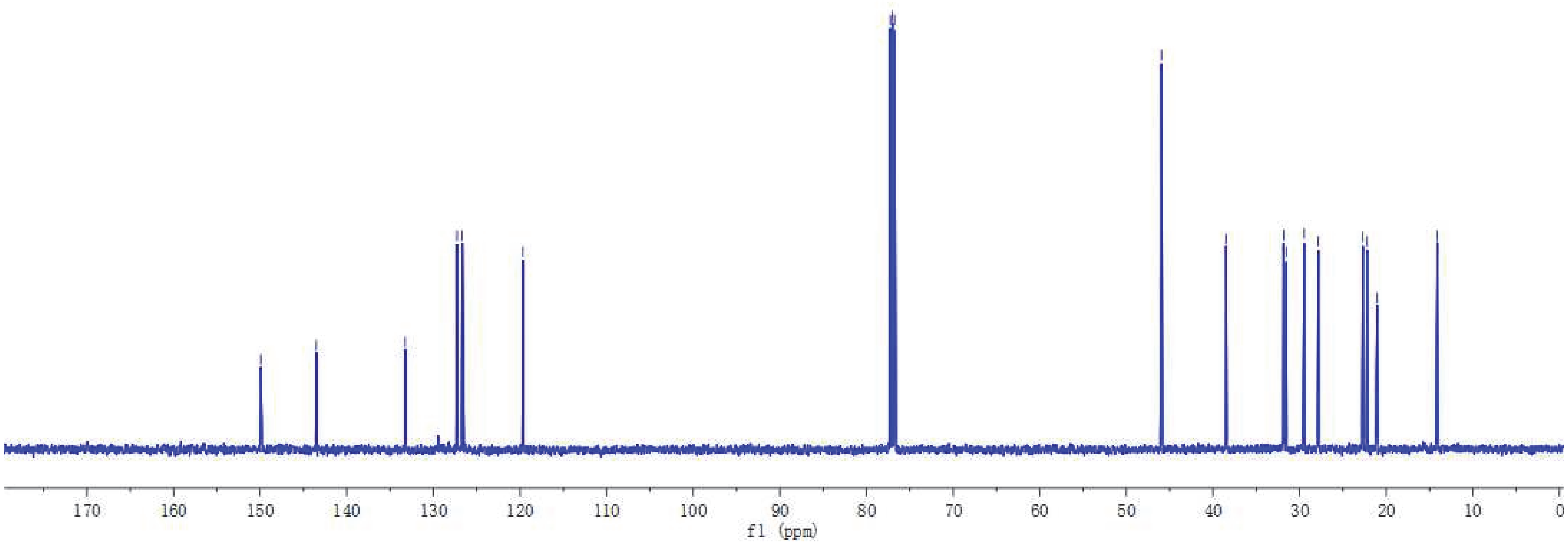
149.914
143.515
133.250
127.271
126.671
119.675
77.254
77.000
76.746
45.953
38.481
31.849
31.539
29.463
27.833
22.699
22.196
21.040
14.099
170
160
150
140
130
120
110
100
90
80
70
60
50
40
30
20
10
0
f1 (ppm)

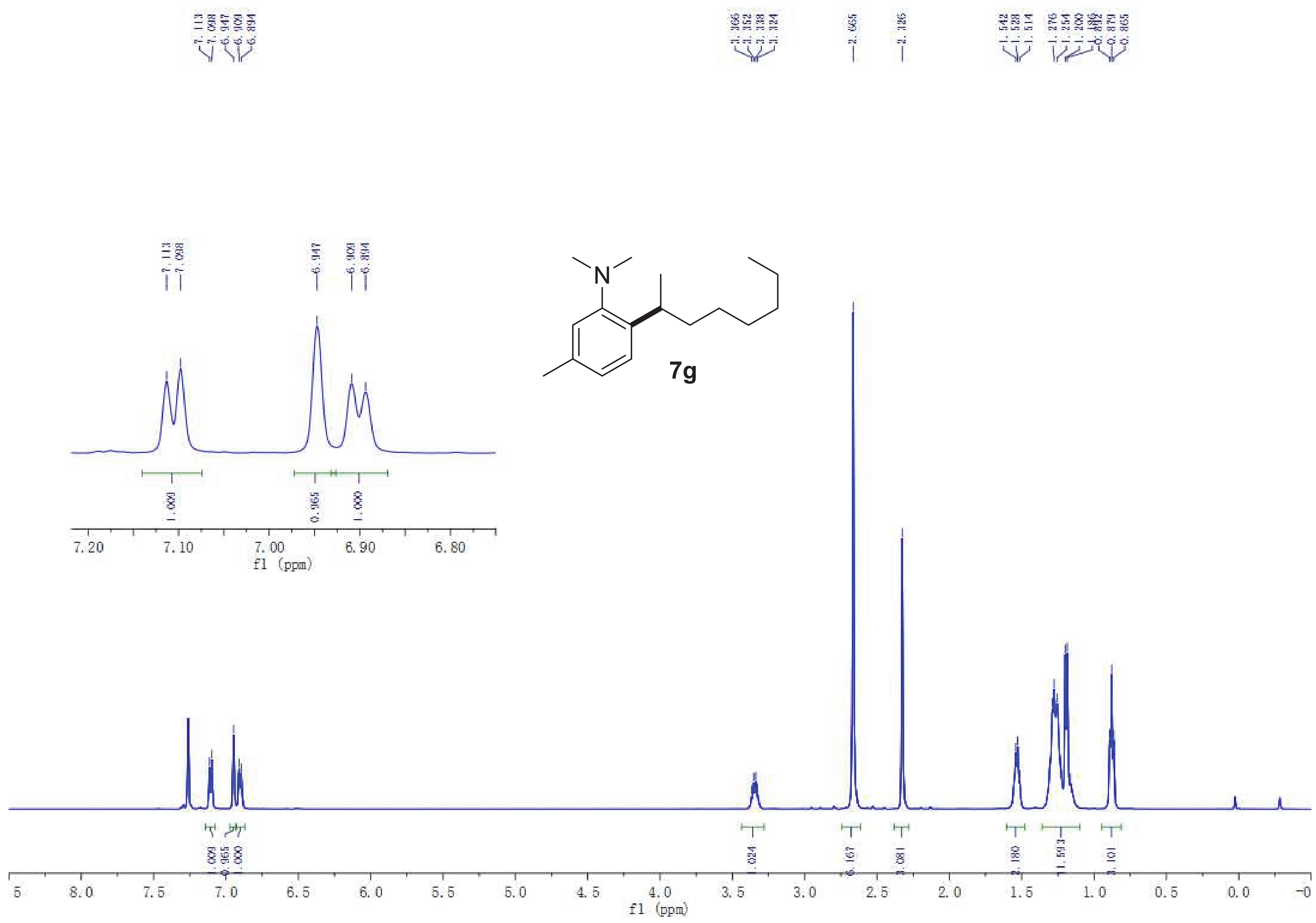
7.113
7.098
6.947
6.909
6.894
3.366
3.352
3.338
3.324
2.665
2.326
1.542
1.528
1.514
1.276
1.254
1.200
1.186
0.892
0.879
0.865
1.009
0.965
1.000
1.024
6.167
3.081
2.180
11.593
3.101
7.20
7.10
7.00
6.90
6.80
f1 (ppm)
8.0
7.5
7.0
6.5
6.0
5.5
5.0
4.5
4.0
3.5
3.0
2.5
2.0
1.5
1.0
0.5
0.0
f1 (ppm)

7g

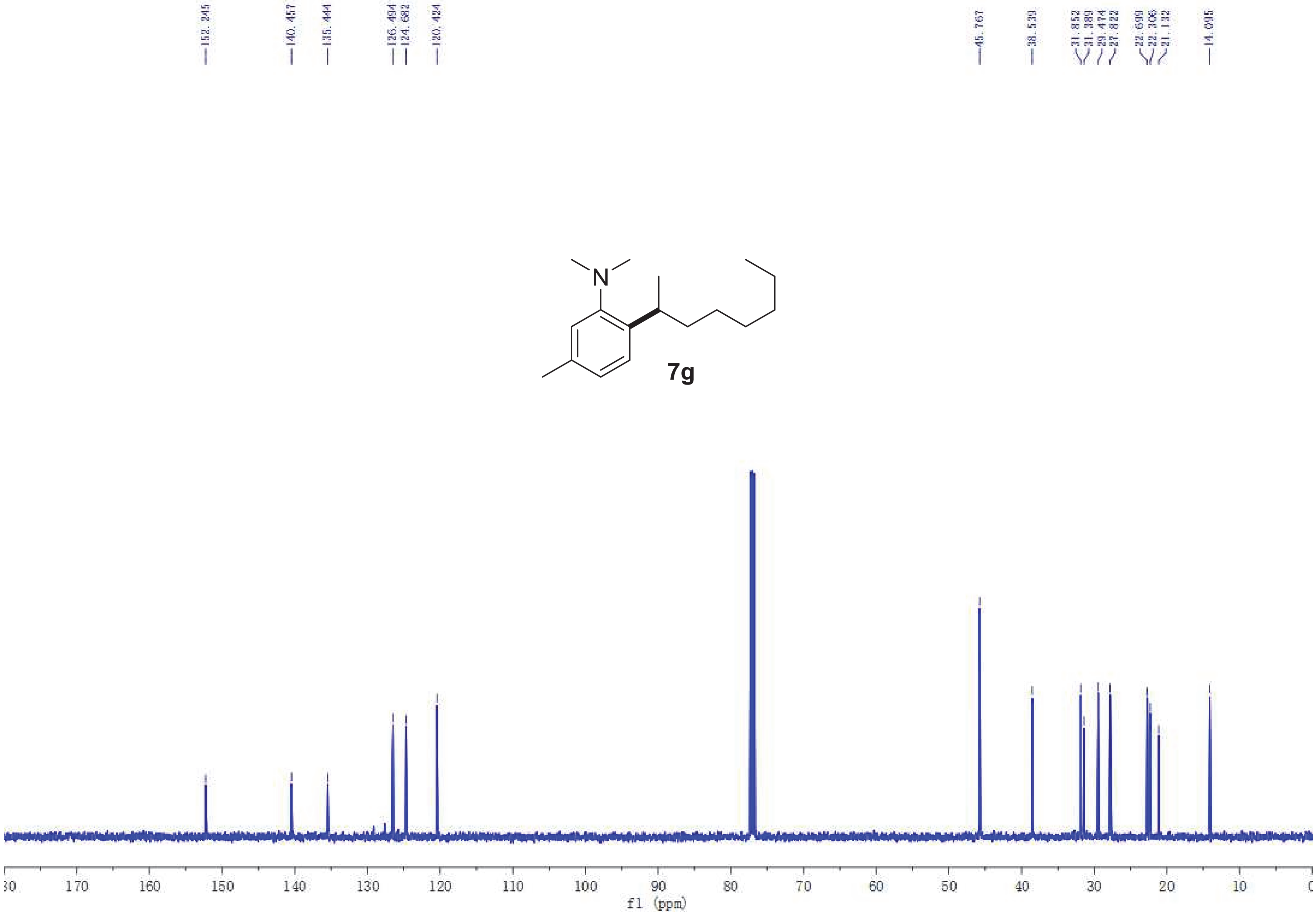

152.245
140.457
135.444
126.494
124.682
120.424
45.767
38.539
31.852
31.389
29.474
27.822
22.699
22.306
21.132
14.095
N
7g
180
170
160
150
140
130
120
110
100
90
80
70
60
50
40
30
20
10
0
f1 (ppm)

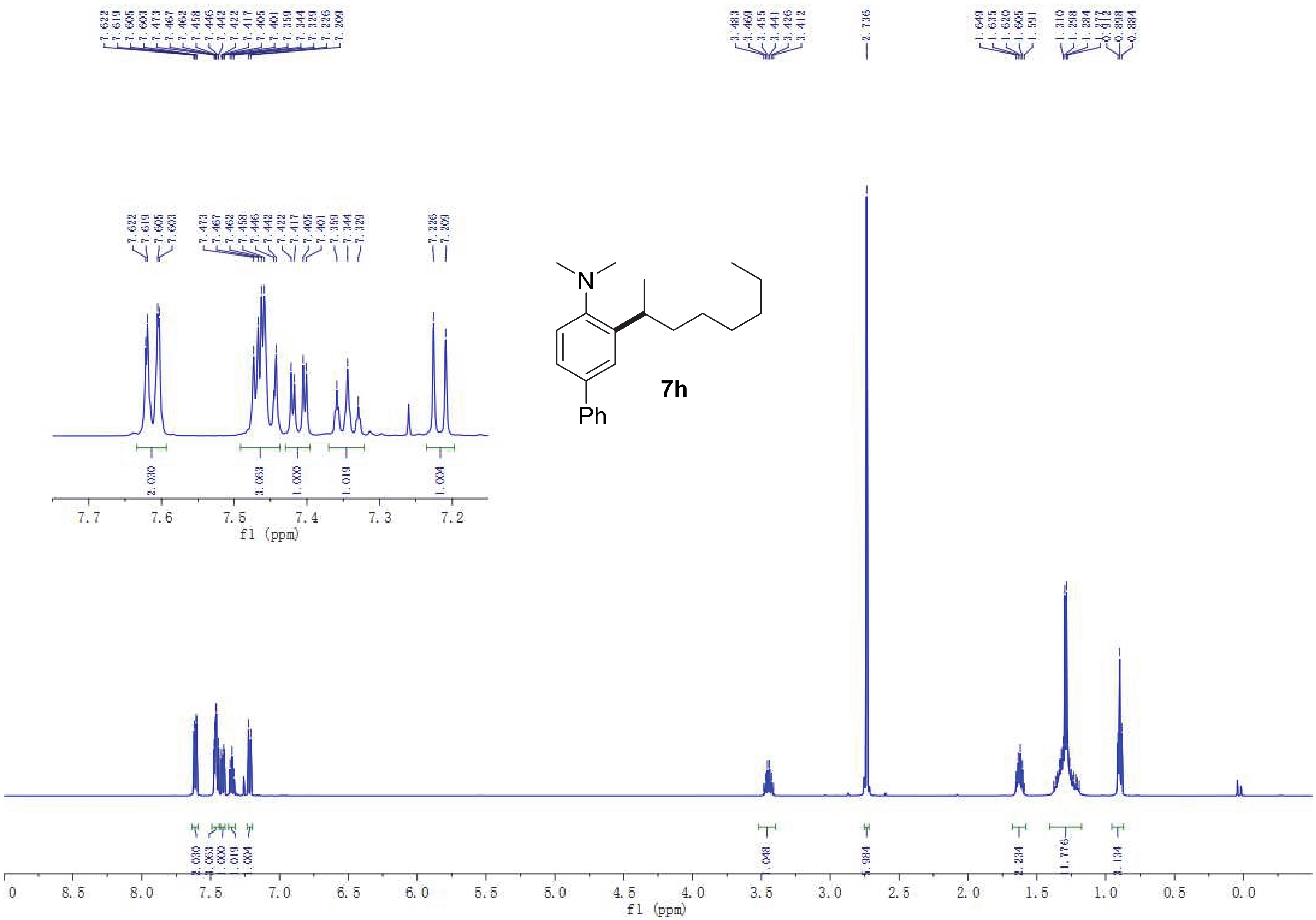

7h

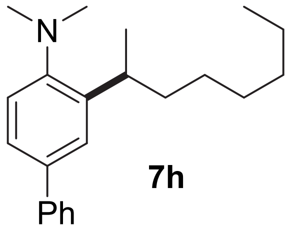

7h

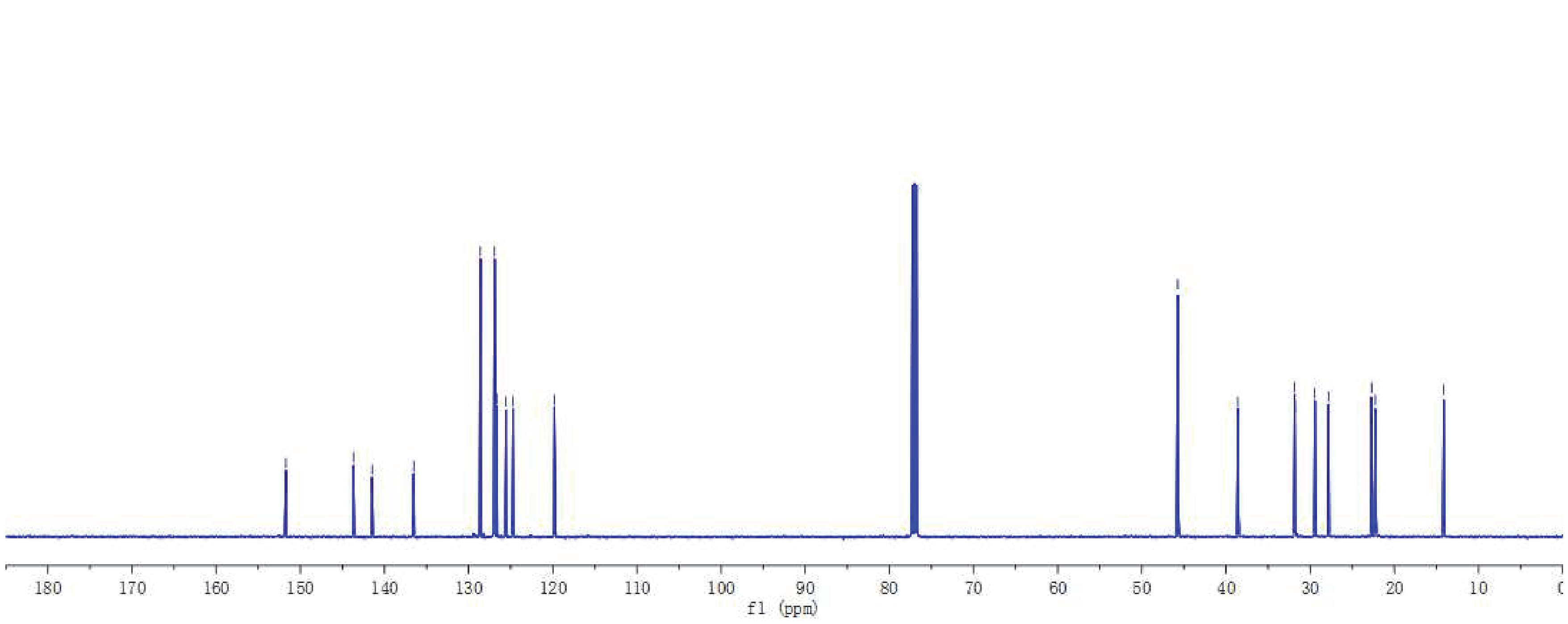
151.727
143.668
141.458
136.521
128.607
126.917
126.664
125.566
124.727
119.815
45.728
38.577
31.835
31.795
29.440
27.799
22.697
22.246
14.128
180
170
160
150
140
130
120
110
100
90
80
70
60
50
40
30
20
10
f1 (ppm)

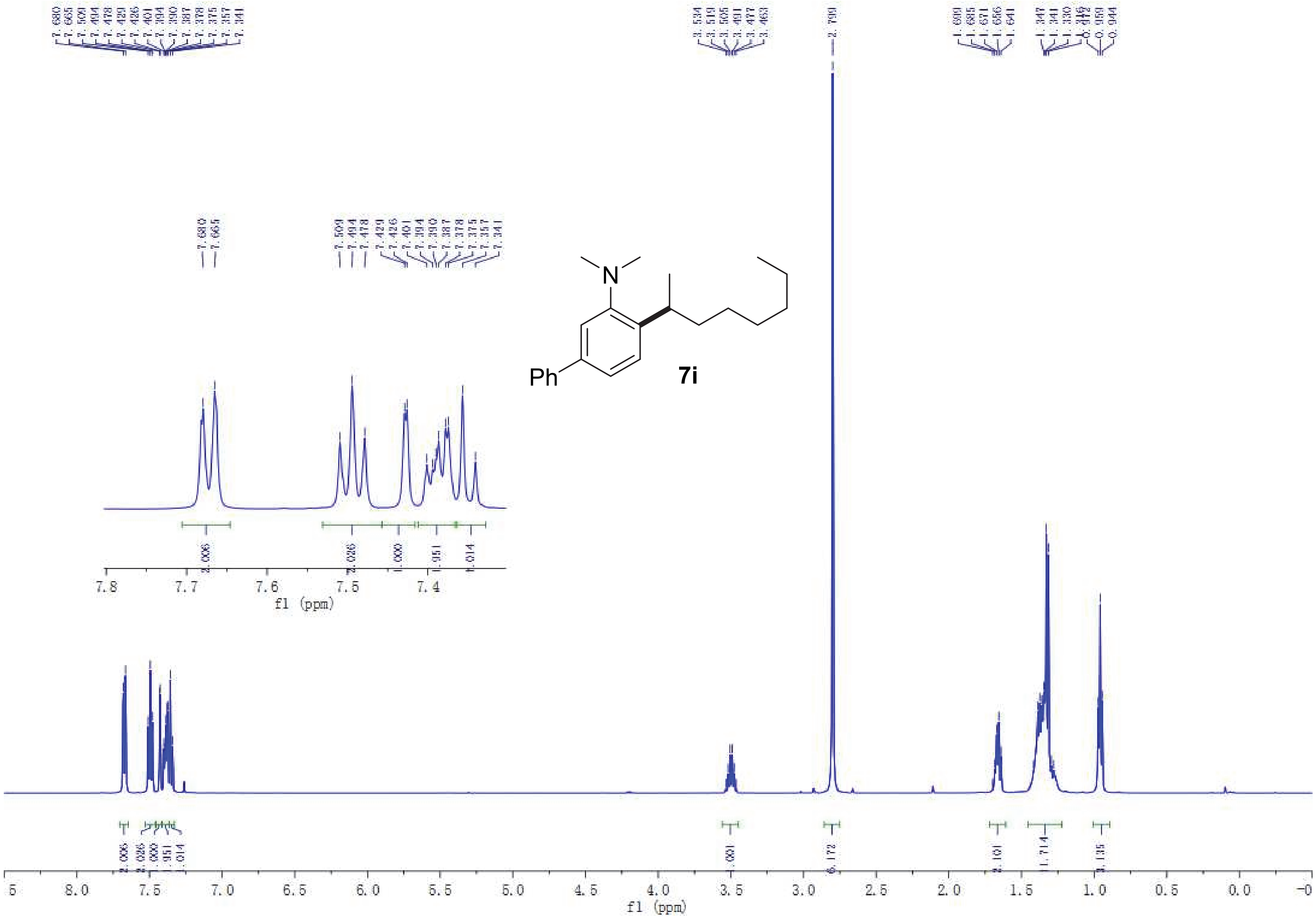
7.680 7.665 7.509 7.494 7.478 7.429 7.426 7.401 7.394 7.390 7.387 7.378 7.375 7.357 7.341
3.534 3.519 3.505 3.491 3.477 3.463
2.799
1.699 1.685 1.671 1.656 1.641
1.347 1.341 1.330 1.316
0.972 0.959 0.944
Ph
N
7i
2.006
2.026
1.000
1.951
1.014
1.001
6.172
2.101
11.714
3.135
f1 (ppm)

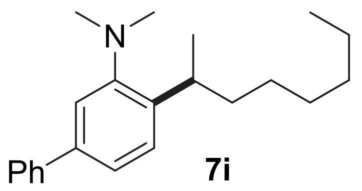
7i

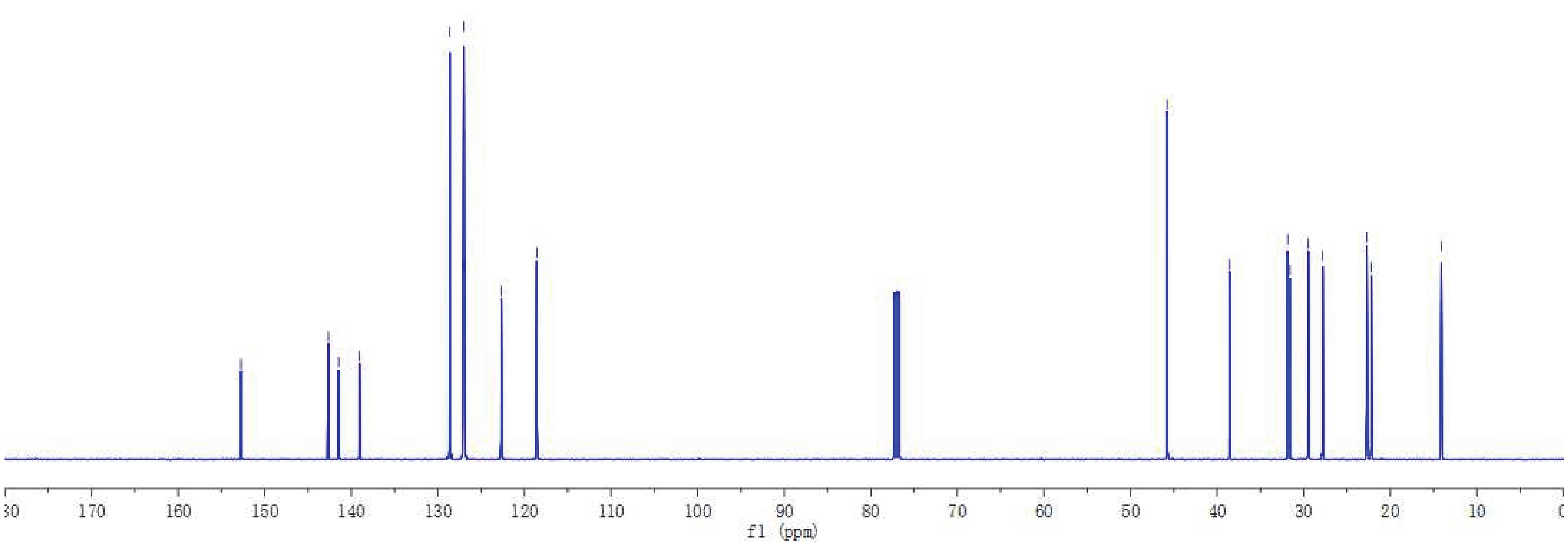
152.730
142.648
141.457
139.051
128.620
127.083
126.988
126.886
122.682
118.576
45.767
38.545
31.858
31.583
29.462
27.815
22.707
22.197
14.107
f1 (ppm)

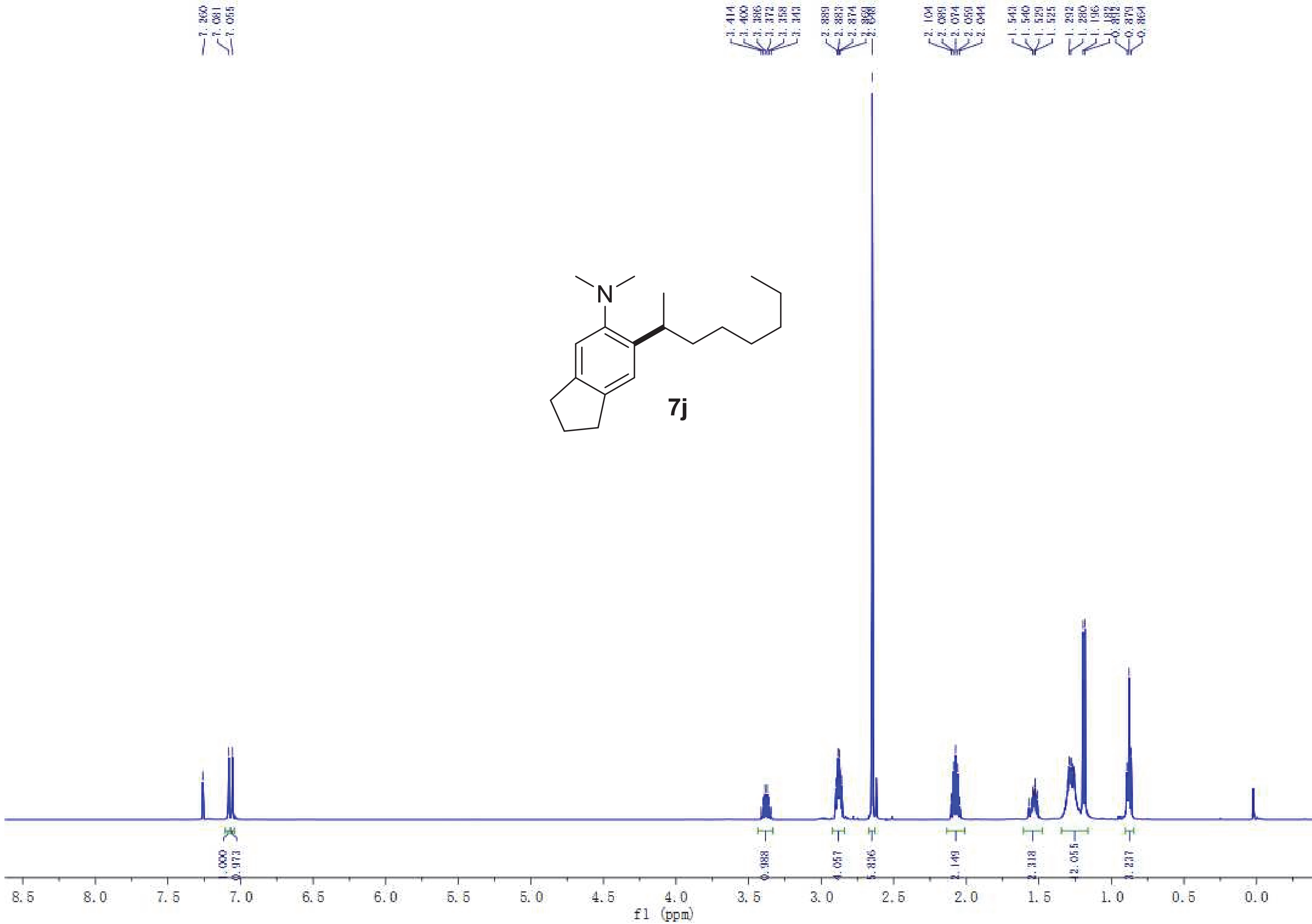
7j
8.5
8.0
7.5
7.0
6.5
6.0
5.5
5.0
4.5
4.0
3.5
3.0
2.5
2.0
1.5
1.0
0.5
0.0
f1 (ppm)

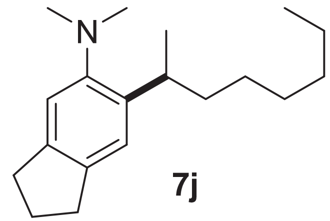

7j

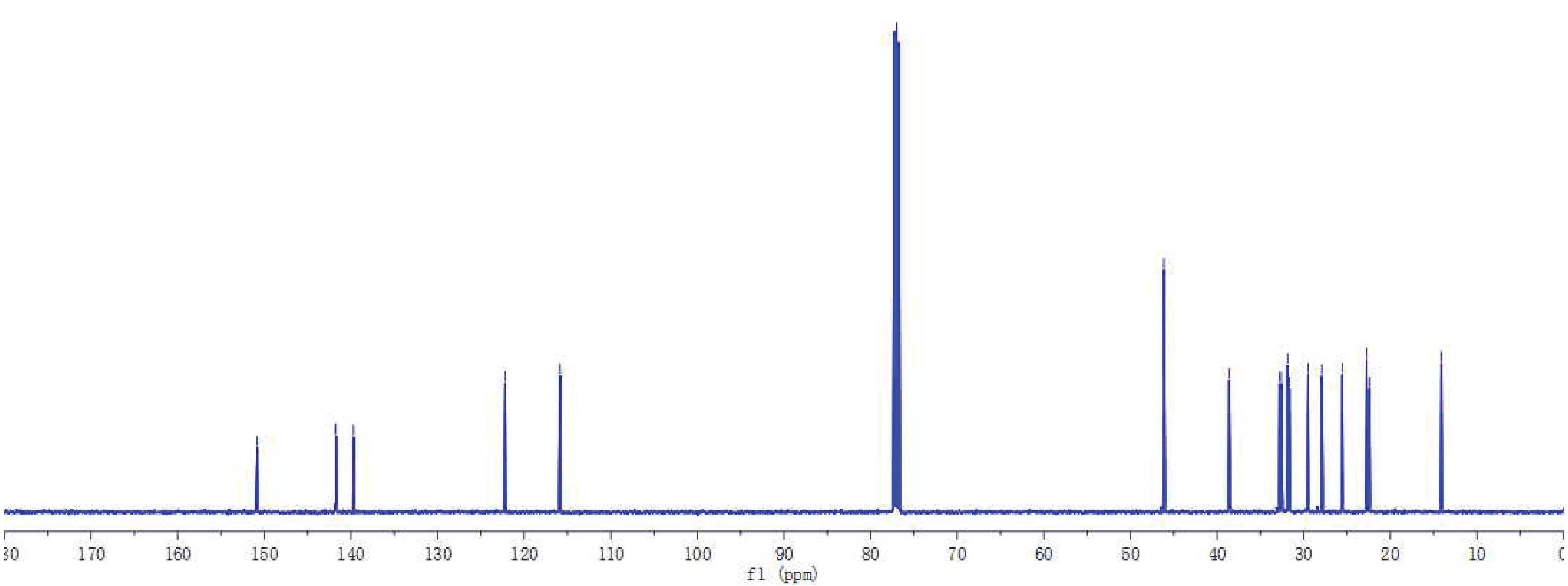
150.810
141.800
141.696
139.688
122.202
115.856
46.125
38.617
32.815
32.566
31.850
31.631
29.499
27.889
25.546
22.711
22.413
14.105
f1 (ppm)

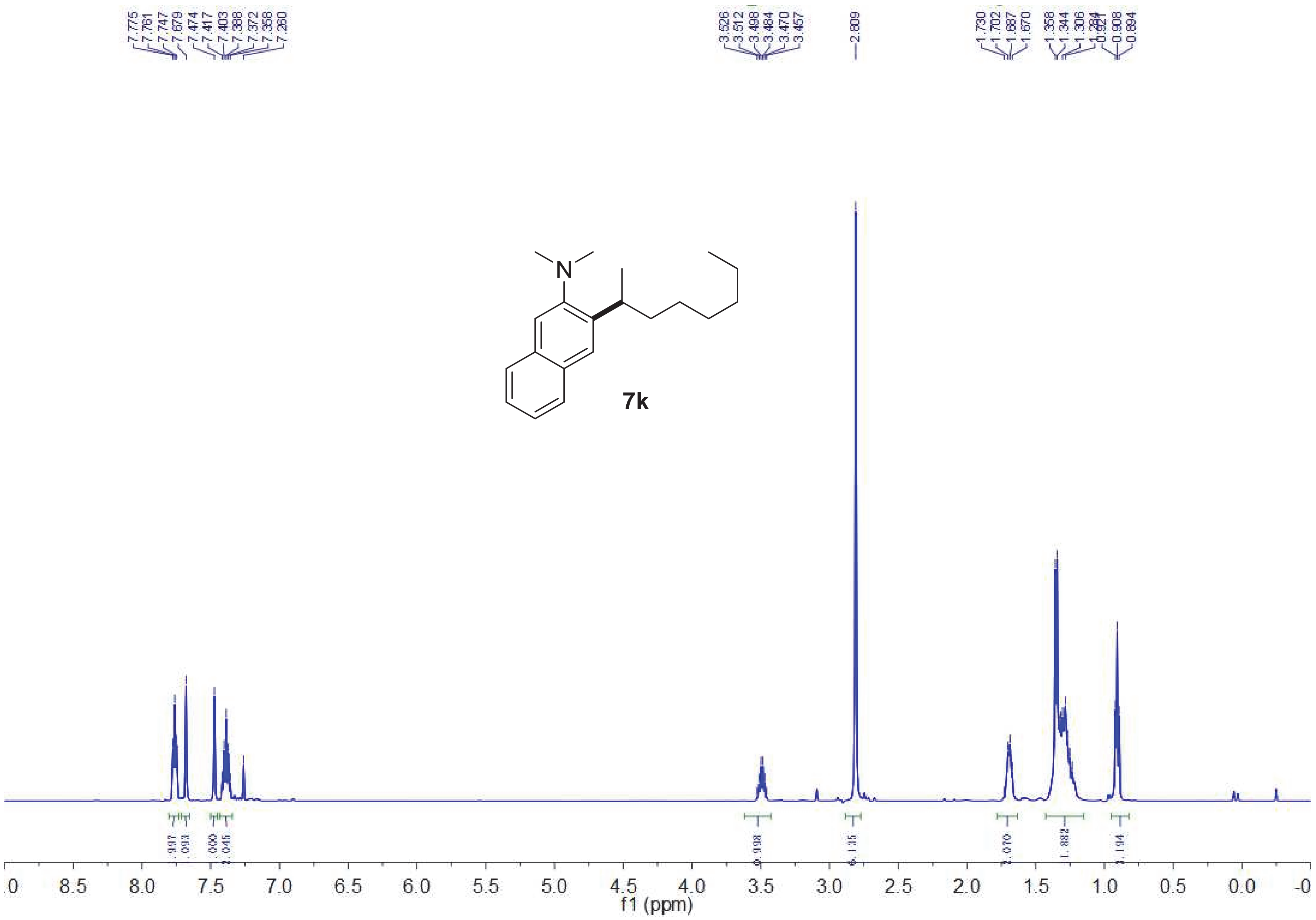
7.775
7.761
7.747
7.679
7.474
7.417
7.403
7.388
7.372
7.358
7.260
3.526
3.512
3.498
3.484
3.470
3.457
2.809
1.730
1.702
1.687
1.670
1.358
1.344
1.306
1.284
0.921
0.908
0.894
.997
.093
.000
2.045
0.998
6.135
2.070
1.882
3.194
.0
8.5
8.0
7.5
7.0
6.5
6.0
5.5
5.0
4.5
4.0
3.5
3.0
2.5
2.0
1.5
1.0
0.5
0.0
-0
f1 (ppm)

7k

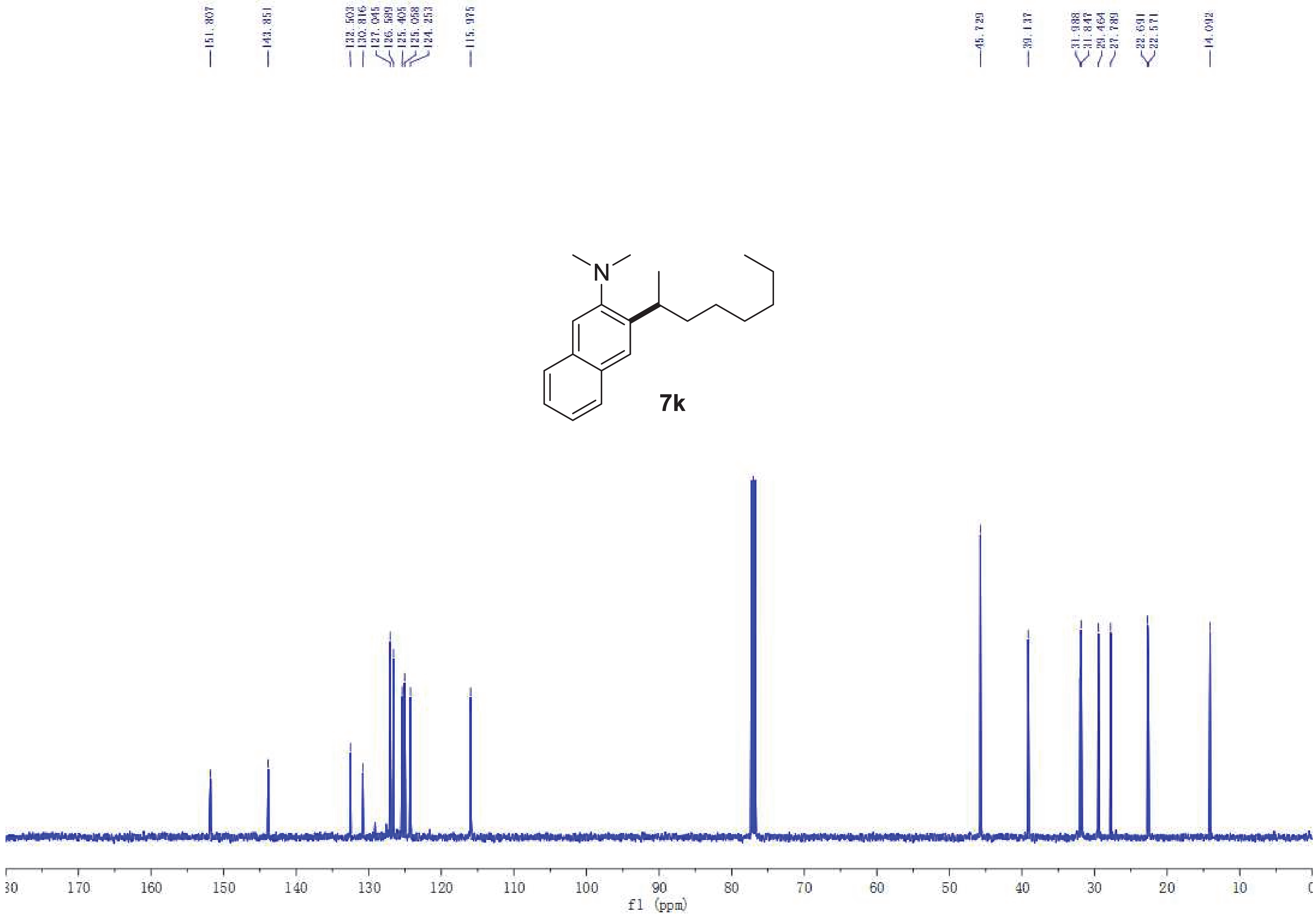
151.807
143.851
132.503
130.816
127.045
126.589
125.405
125.058
124.253
115.975
45.729
39.137
31.988
31.847
29.464
27.789
22.691
22.571
14.092
N
f1 (ppm)

7k

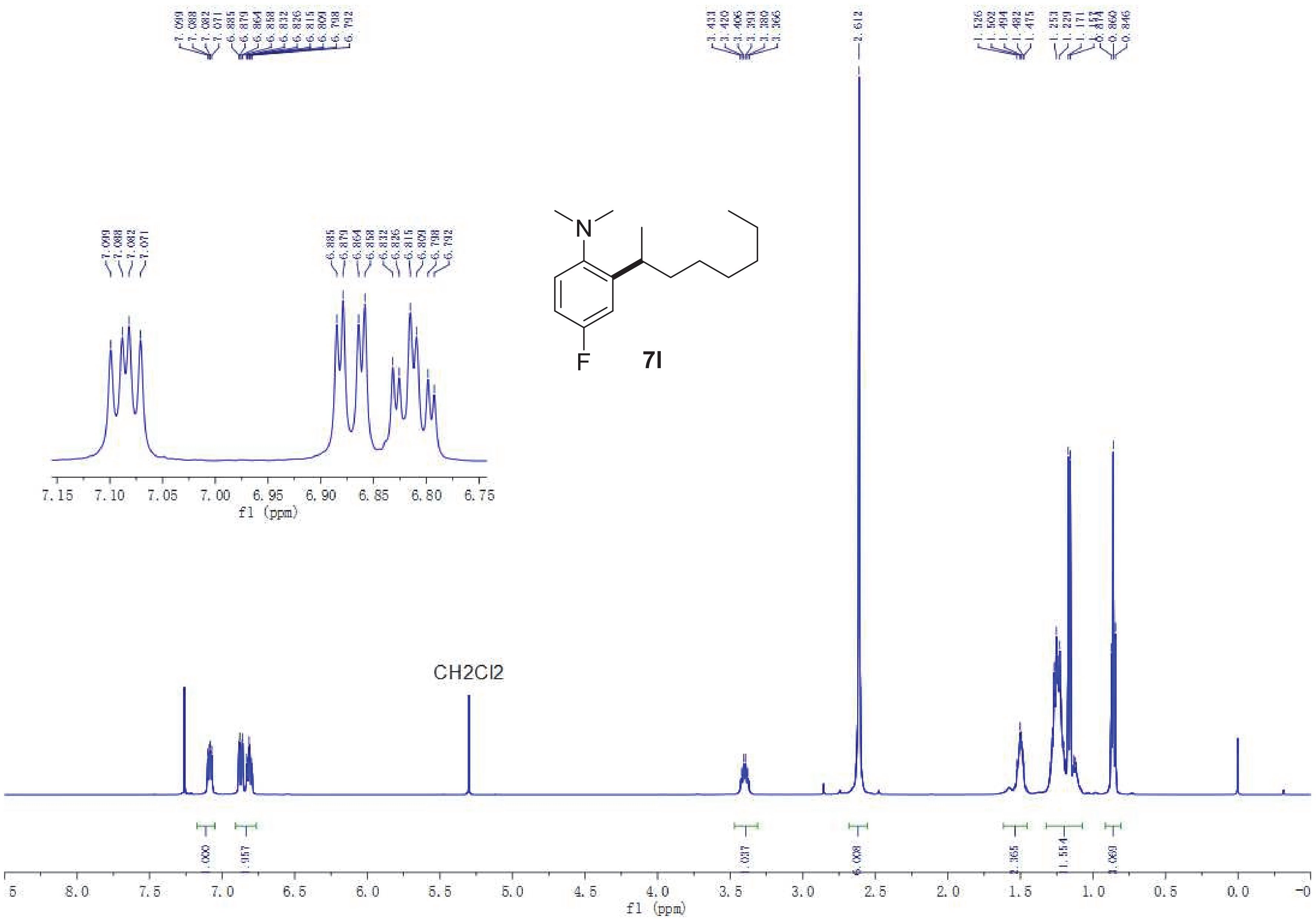
7.099
7.088
7.082
7.071
6.885
6.879
6.864
6.858
6.832
6.826
6.815
6.809
6.798
6.792
3.433
3.420
3.406
3.393
3.380
3.366
2.612
1.526
1.502
1.494
1.482
1.475
1.253
1.229
1.171
1.157
0.874
0.860
0.846
N
F
CH2Cl2
1.000
1.957
1.037
6.008
2.365
11.554
3.060
f1 (ppm)

7l

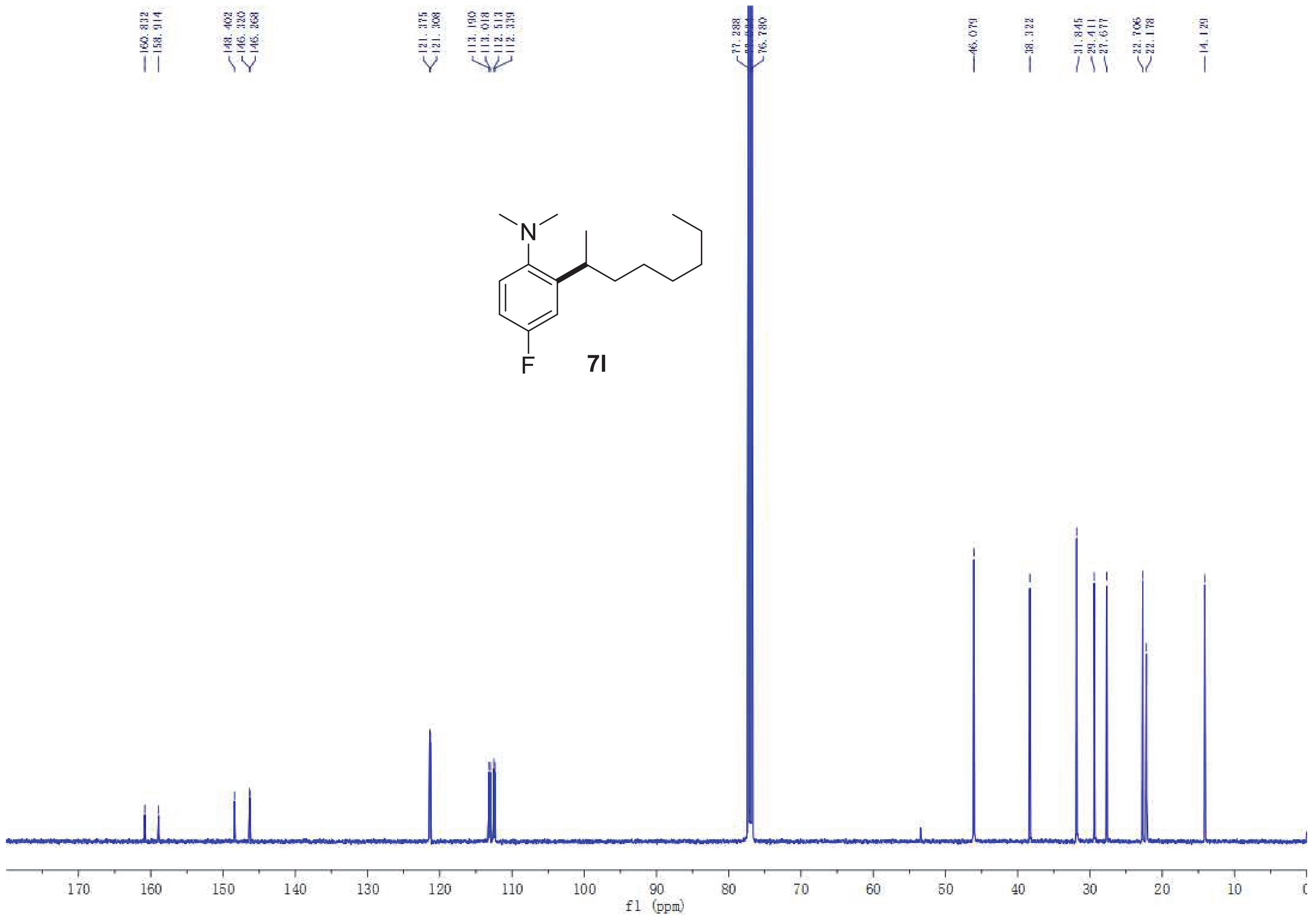

160.832
158.914
148.402
146.320
146.268
121.375
121.308
113.190
113.018
112.513
112.339
77.288
76.780
46.079
38.322
31.845
29.411
27.677
22.706
22.178
14.129
N
F
170 160 150 140 130 120 110 100 90 80 70 60 50 40 30 20 10
f1 (ppm)

7l

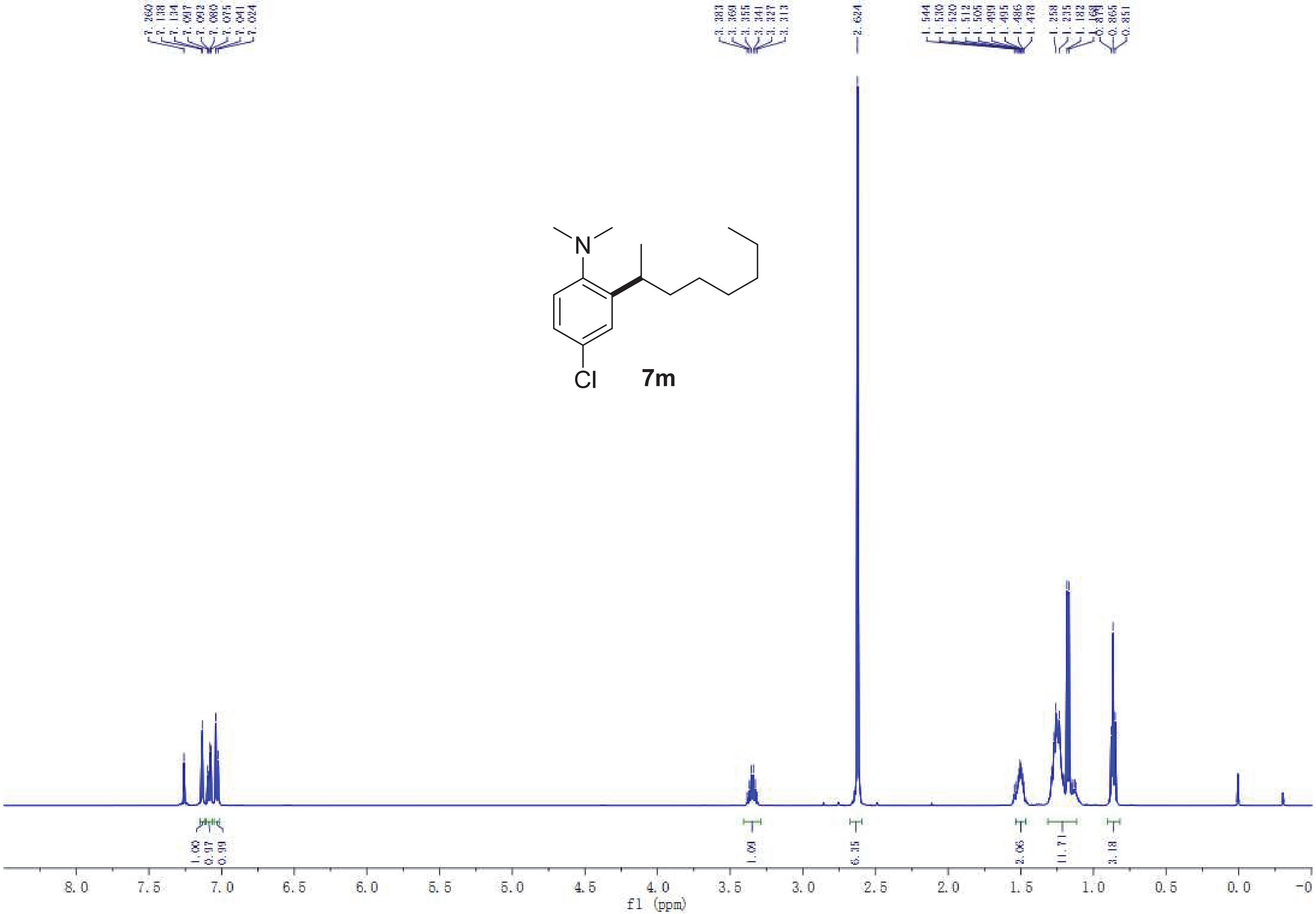

7m

7.260
7.138
7.134
7.097
7.092
7.080
7.075
7.041
7.024
3.383
3.369
3.355
3.341
3.327
3.313
2.624
1.544
1.530
1.520
1.512
1.505
1.499
1.495
1.486
1.478
1.258
1.235
1.182
1.168
0.879
0.865
0.851
1.00
0.97
0.99
1.00
6.15
2.06
11.71
3.18
8.0
7.5
7.0
6.5
6.0
5.5
5.0
4.5
4.0
3.5
3.0
2.5
2.0
1.5
1.0
0.5
0.0
-0
f1 (ppm)

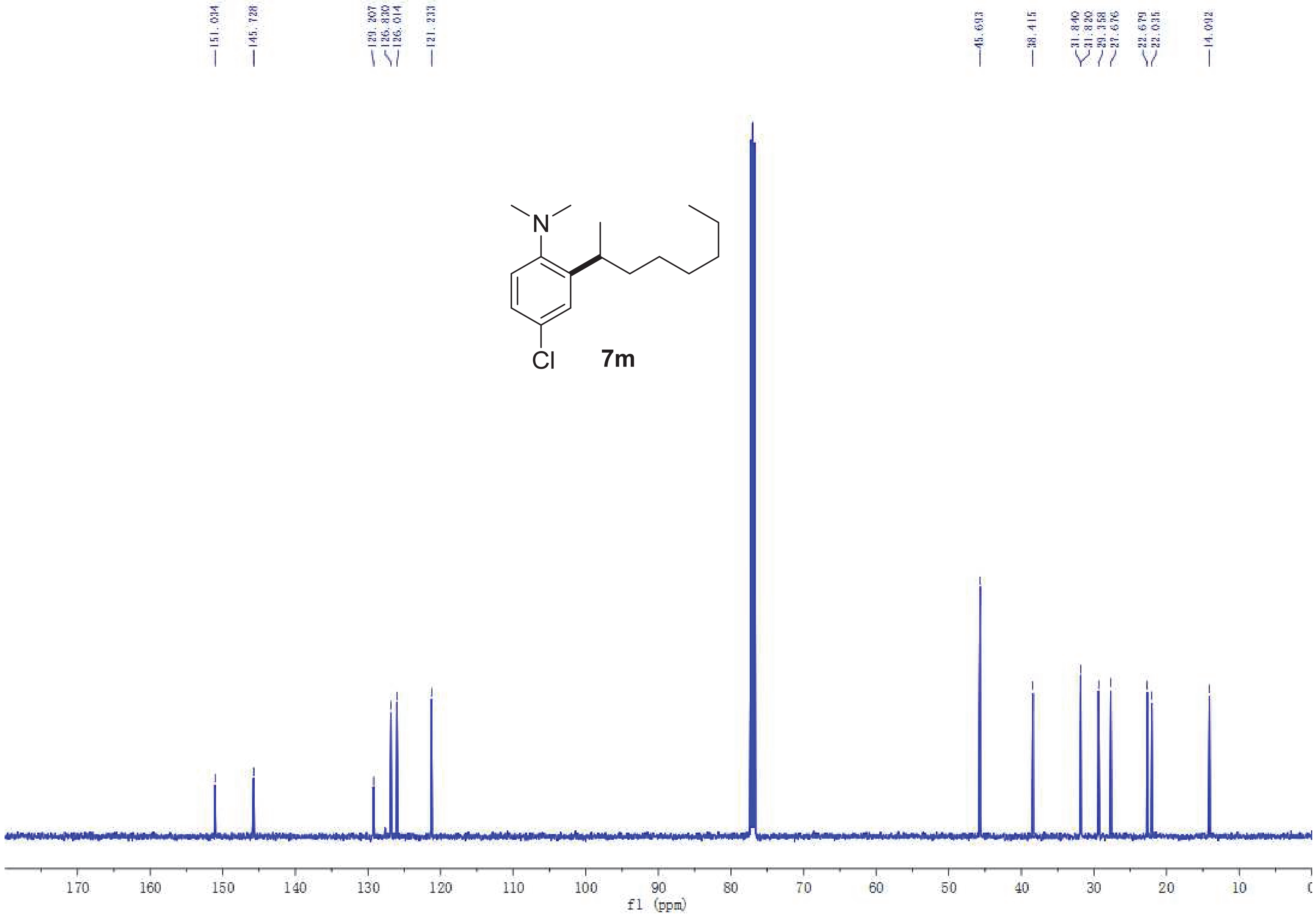
151.034
145.728
129.207
126.830
126.014
121.233
45.693
38.415
31.840
31.820
29.358
27.676
22.679
22.035
14.092
N
Cl
170
160
150
140
130
120
110
100
90
80
70
60
50
40
30
20
10
f1 (ppm)

7m

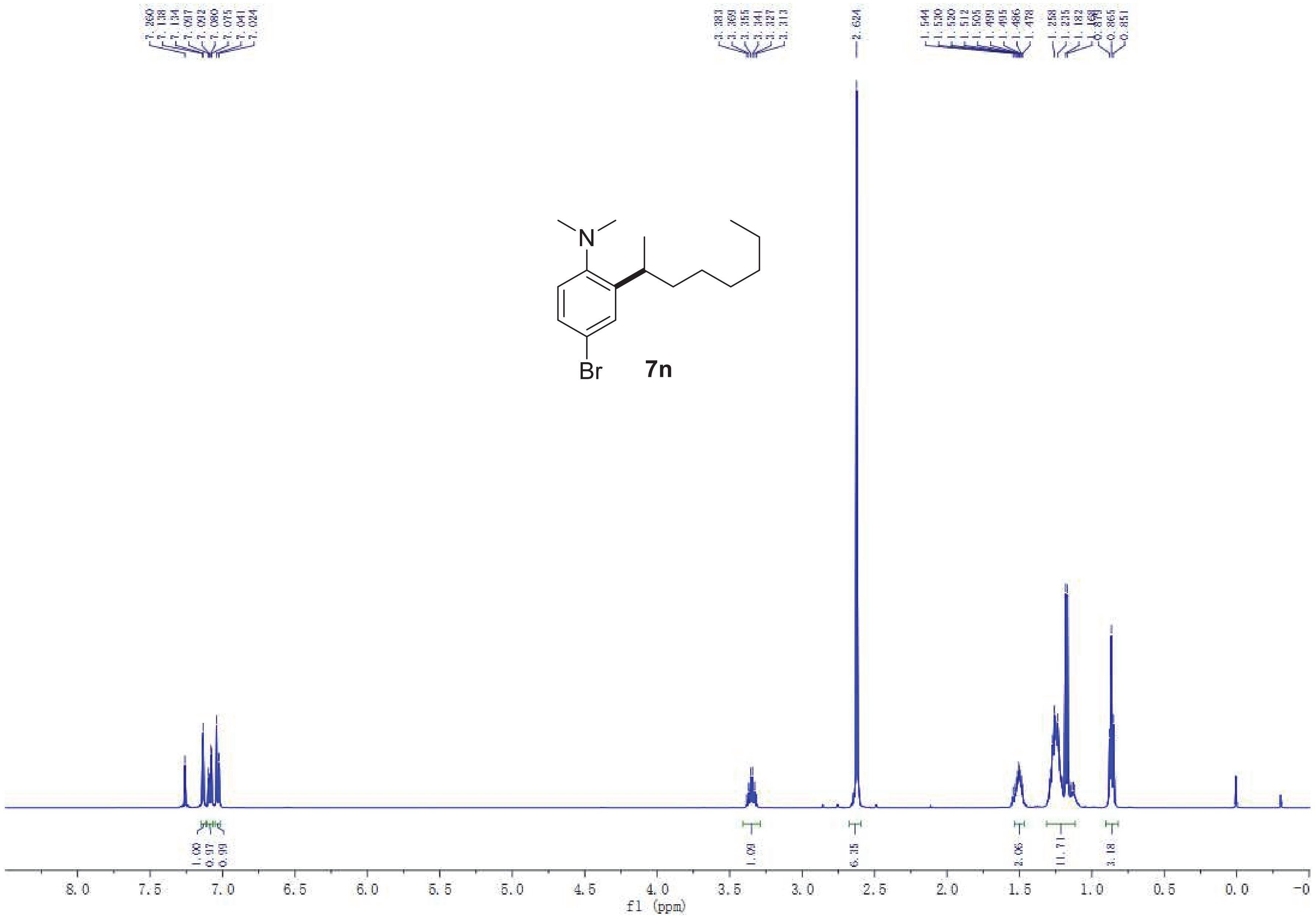
7.260
7.138
7.134
7.097
7.092
7.080
7.075
7.041
7.024
3.383
3.369
3.355
3.341
3.327
3.313
2.624
1.544
1.530
1.520
1.512
1.505
1.499
1.495
1.486
1.478
1.258
1.235
1.182
1.168
0.879
0.865
0.851
1.00
0.97
0.99
1.09
6.35
2.06
11.71
3.18
8.0 7.5 7.0 6.5 6.0 5.5 5.0 4.5 4.0 3.5 3.0 2.5 2.0 1.5 1.0 0.5 0.0 -0
f1 (ppm)

7n

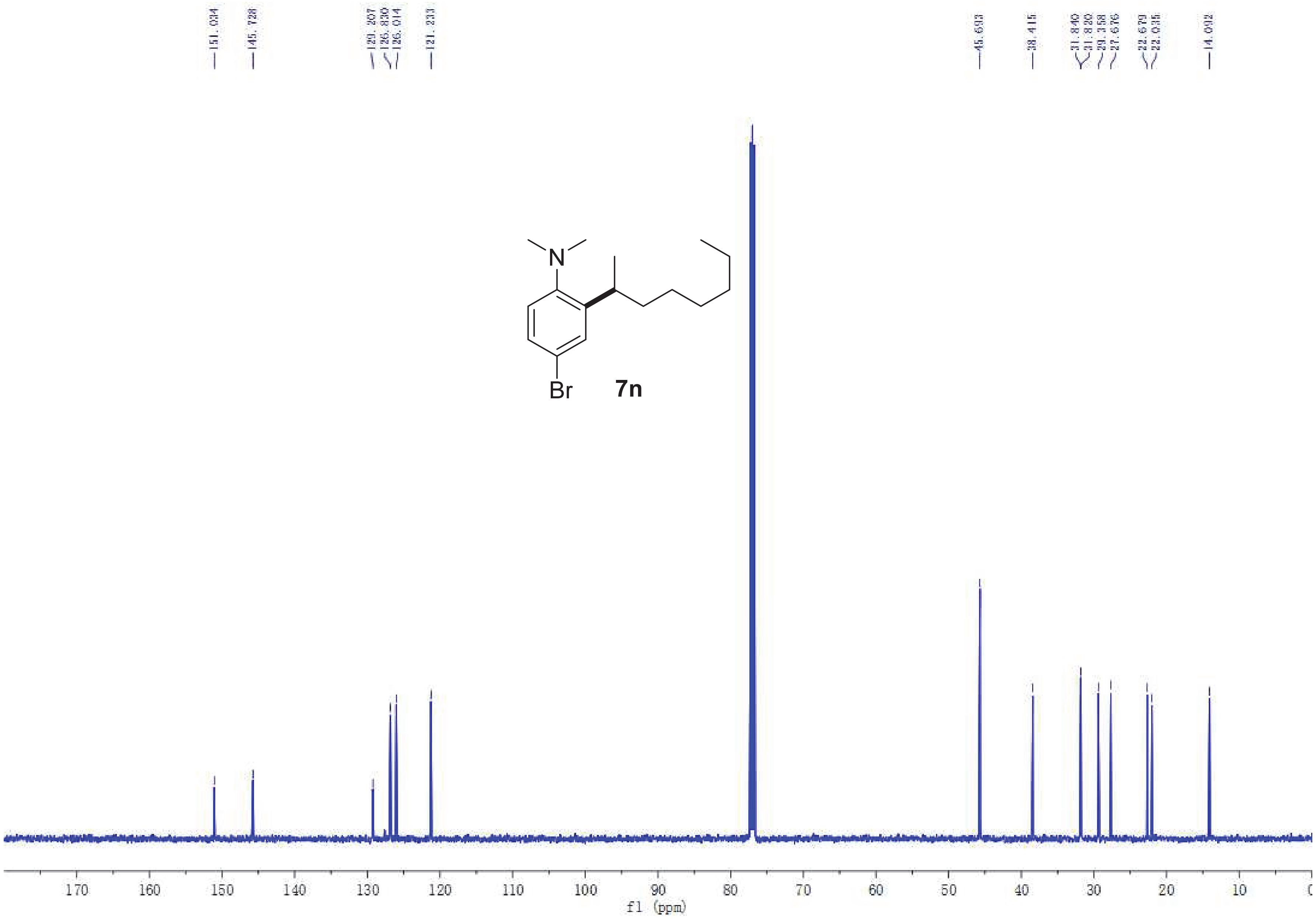
151.034
145.728
129.207
126.830
126.014
121.233
45.693
38.415
31.840
31.820
29.358
27.676
22.679
22.035
14.092
N
Br
7n
170 160 150 140 130 120 110 100 90 80 70 60 50 40 30 20 10
f1 (ppm)

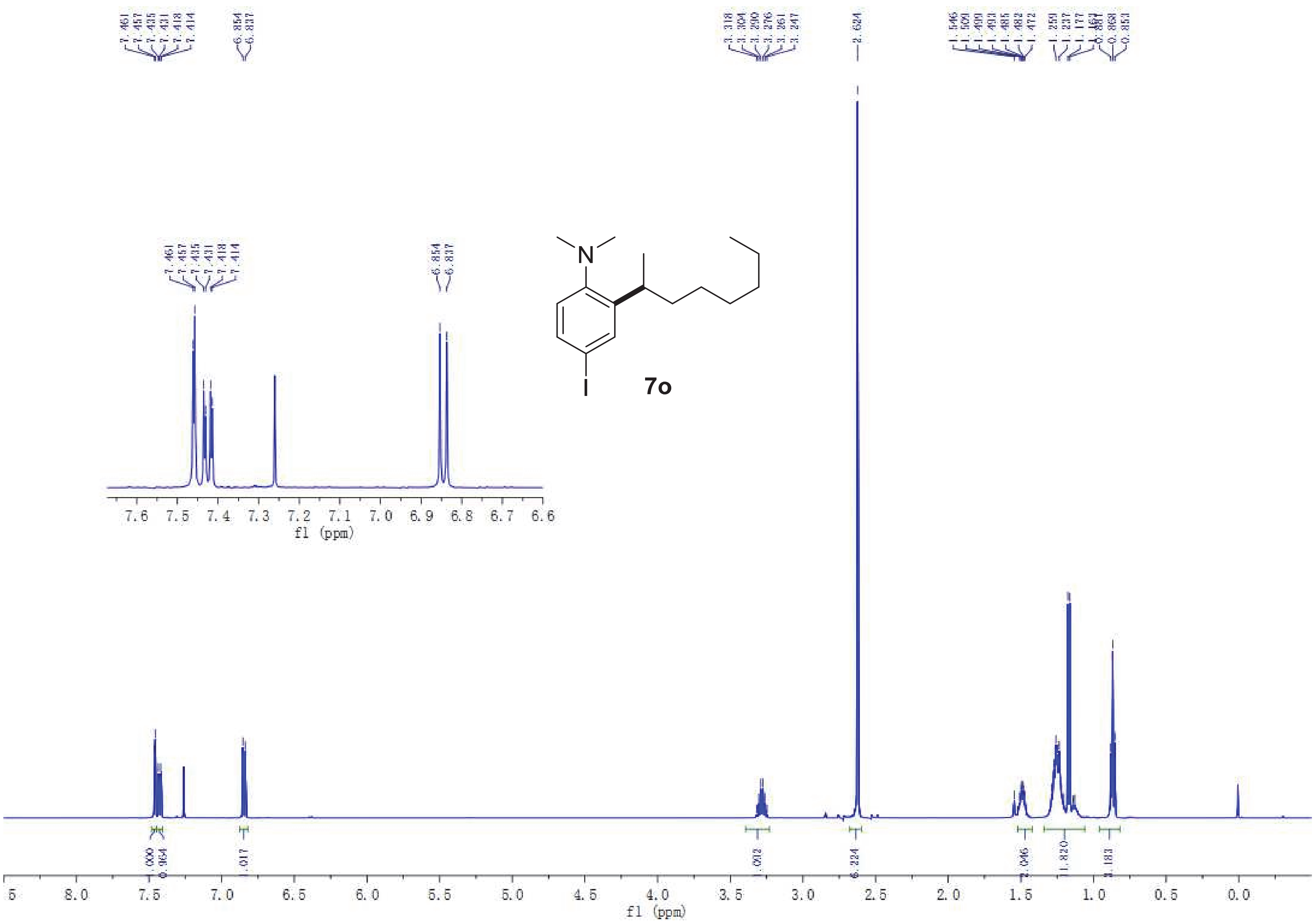
7.461
7.457
7.435
7.431
7.418
7.414
6.854
6.837
3.318
3.304
3.290
3.276
3.261
3.247
2.624
1.546
1.509
1.499
1.493
1.485
1.482
1.472
1.259
1.237
1.177
1.163
0.881
0.868
0.853
N
I
7o
f1 (ppm)
1.000
0.964
1.017
1.003
6.224
2.046
11.820
3.183

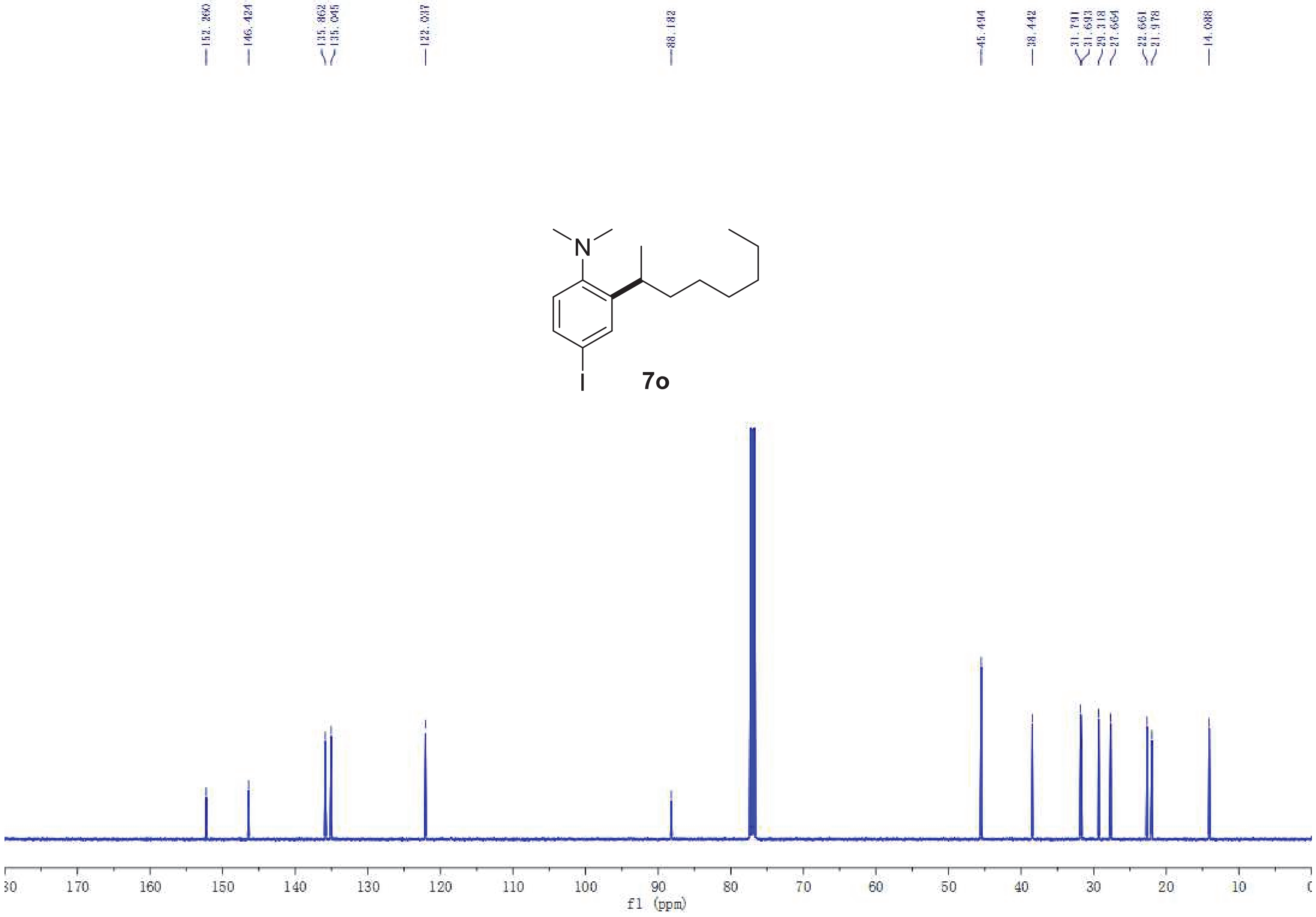
152.260
146.424
135.862
135.045
122.037
88.182
45.494
38.442
31.791
31.693
29.318
27.664
22.661
21.978
14.088
N
I
7o
f1 (ppm)

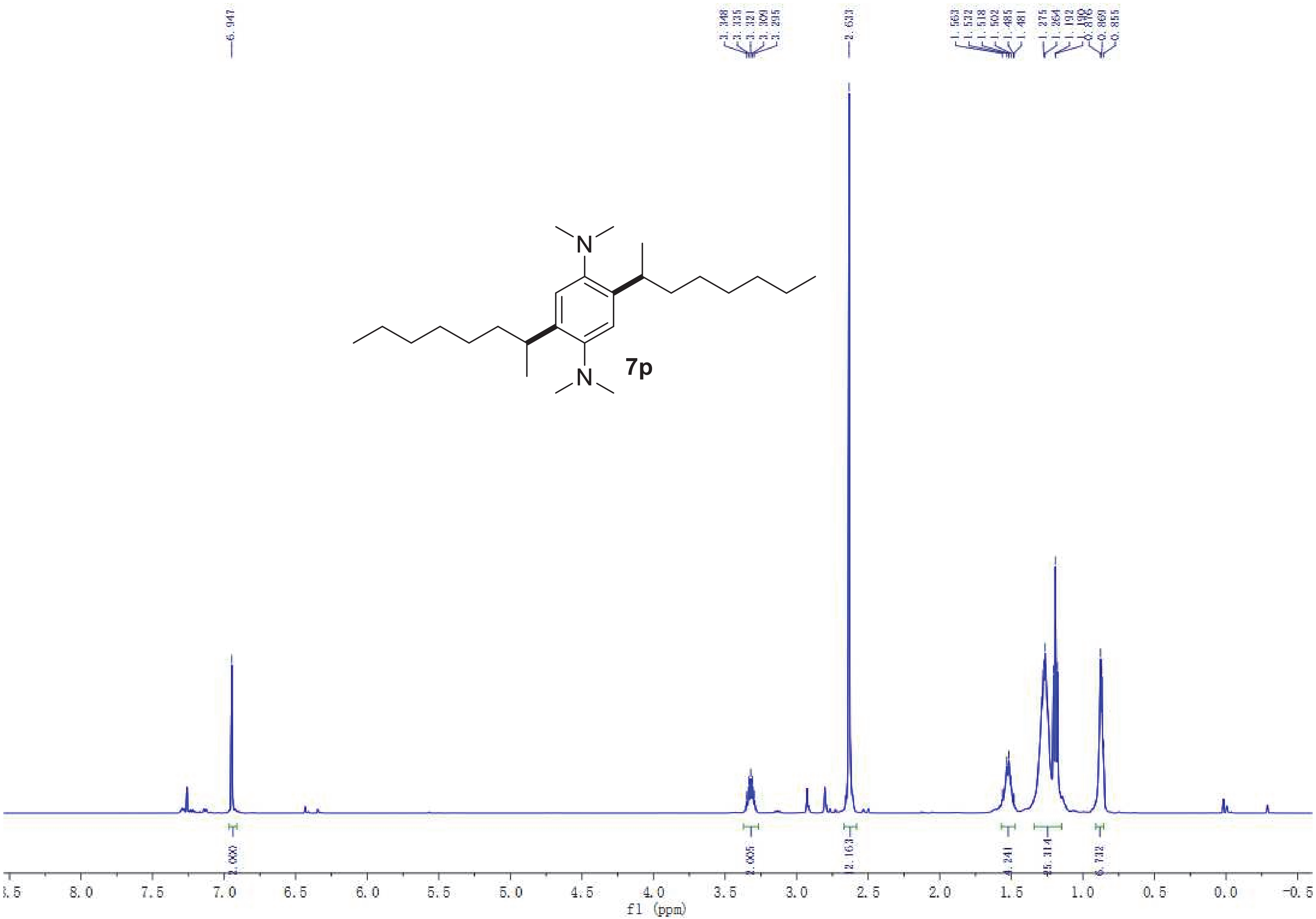
6.947
3.348
3.335
3.321
3.309
3.295
2.633
1.563
1.532
1.518
1.502
1.485
1.481
1.275
1.264
1.192
1.180
0.876
0.869
0.855
N
N
7p
2.000
2.005
12.163
4.241
25.314
6.732
f1 (ppm)

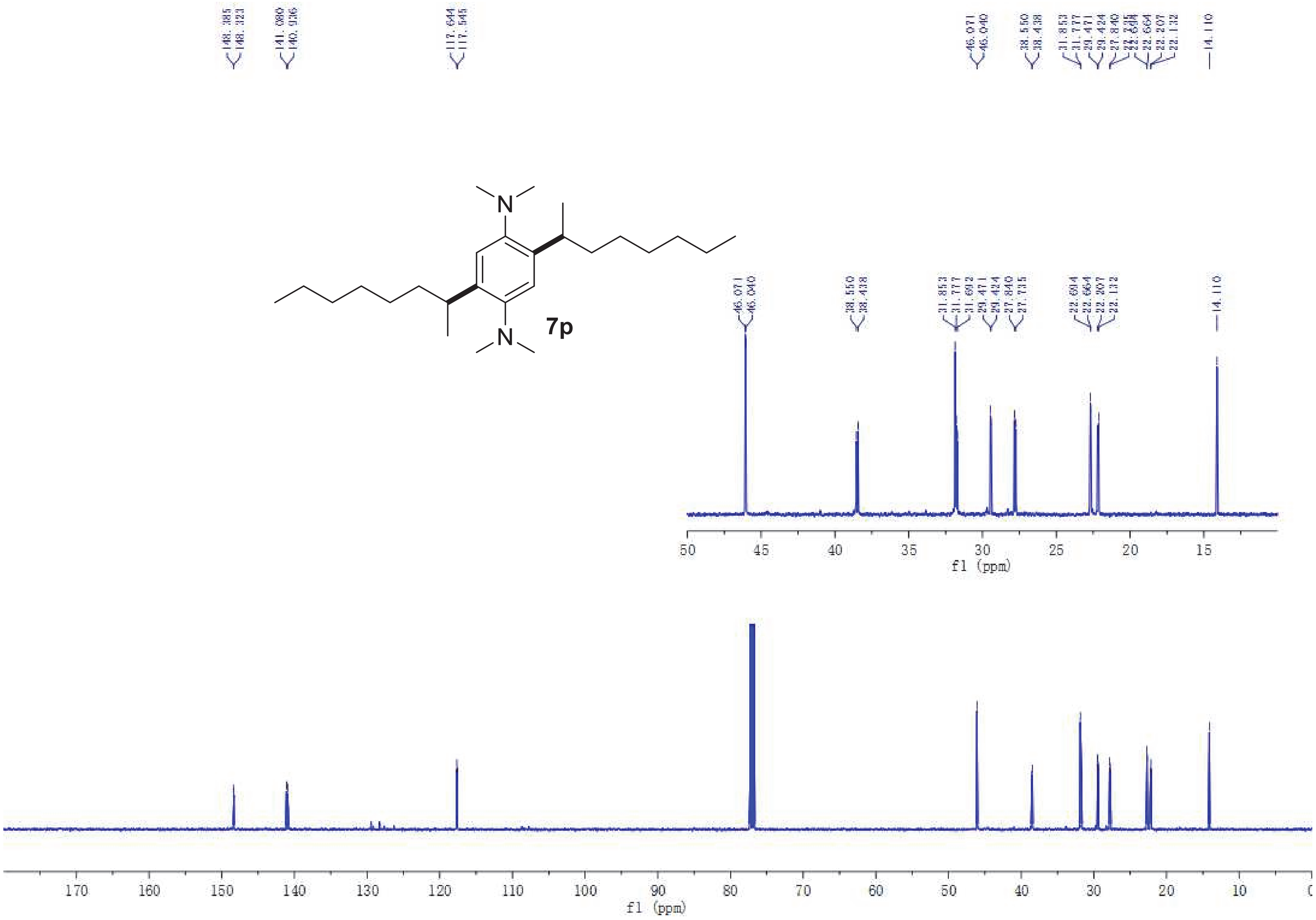
7p
148.385
148.323
141.080
140.936
117.644
117.545
46.071
46.040
38.550
38.438
31.853
31.777
31.692
29.471
29.424
27.840
27.735
22.694
22.664
22.207
22.132
14.110
f1 (ppm)
f1 (ppm)

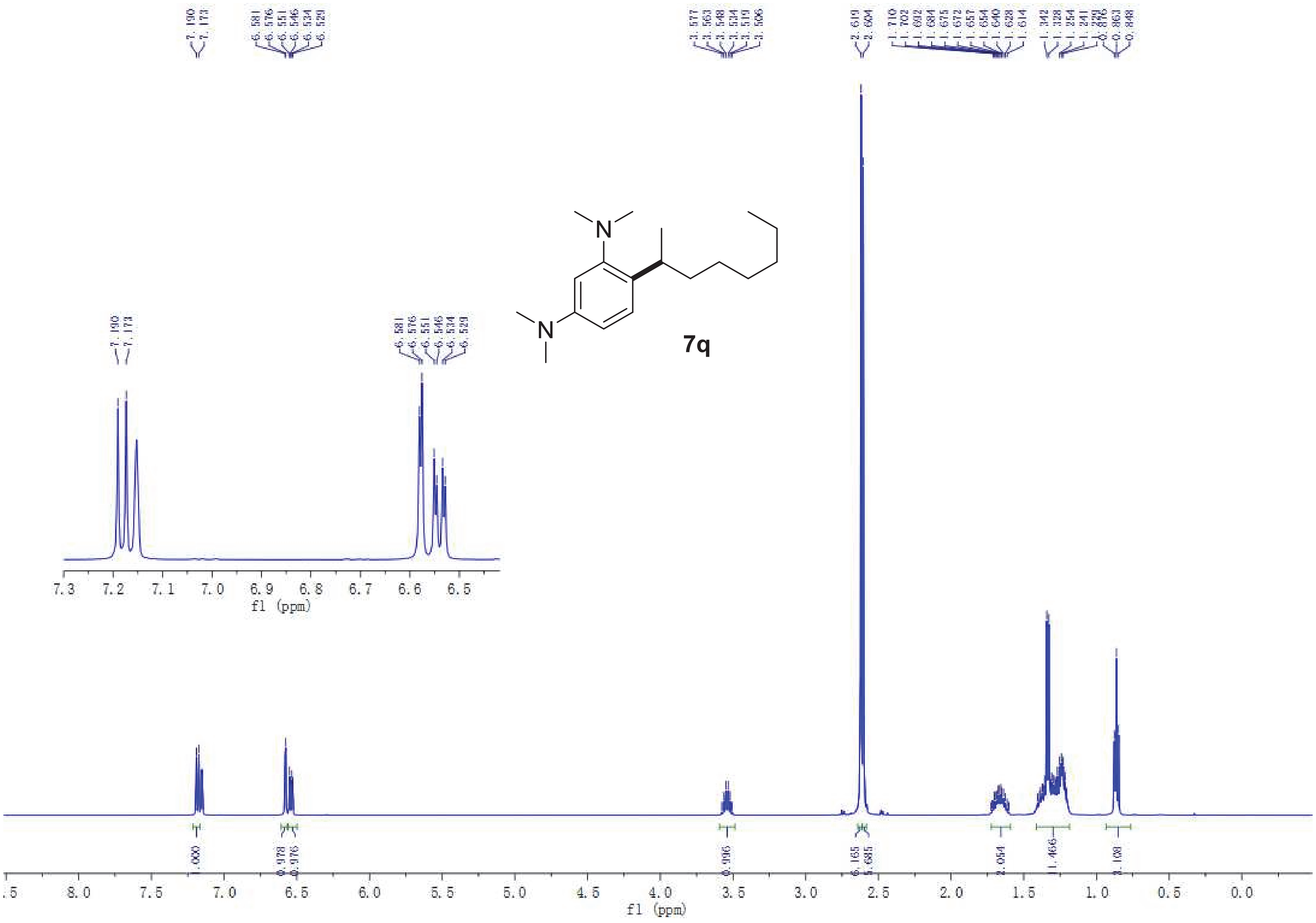

7q

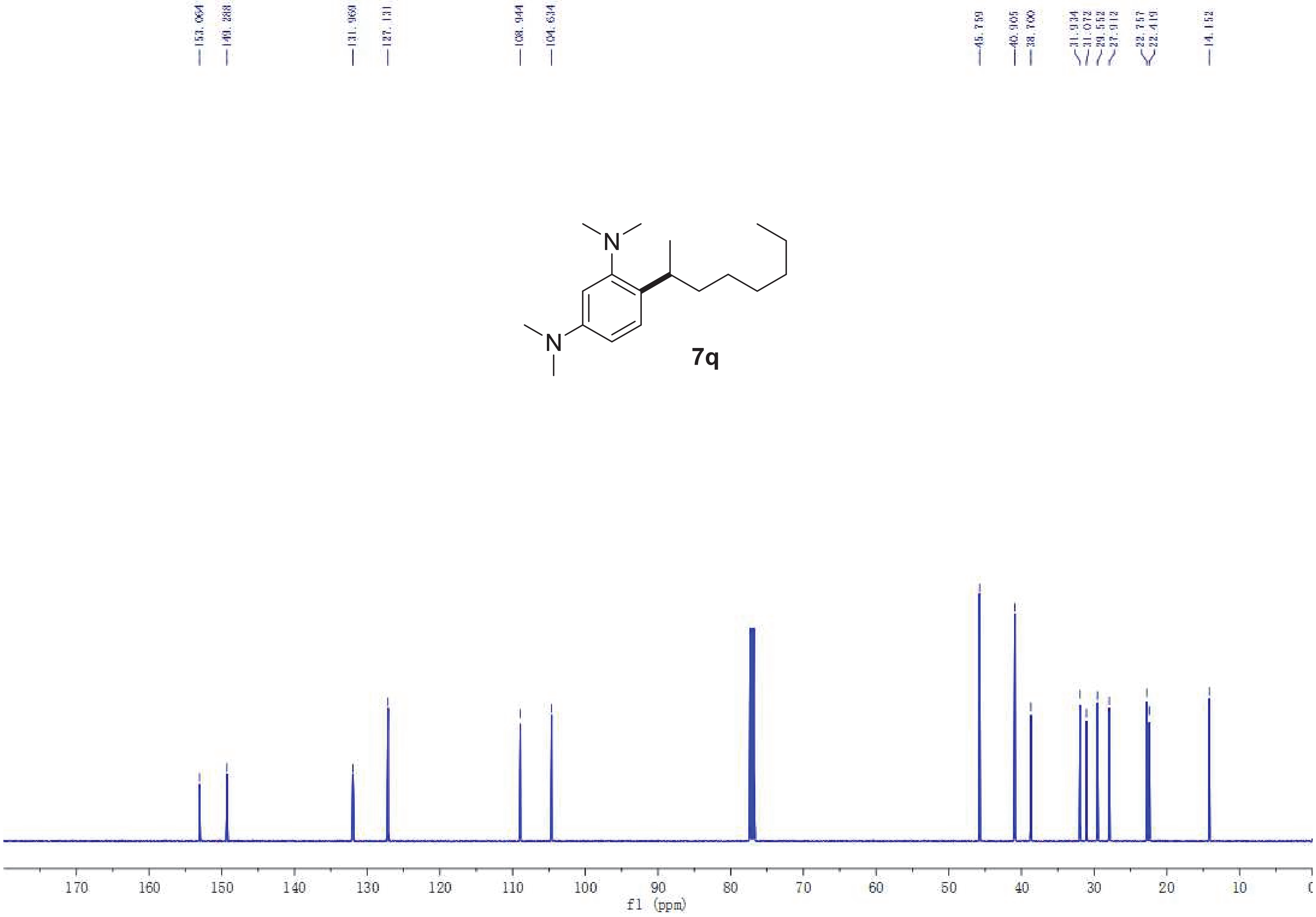
153.064
149.288
131.960
127.131
108.944
104.634
45.759
40.905
38.700
31.934
31.072
29.552
27.912
22.757
22.419
14.152
N
N
7q
170
160
150
140
130
120
110
100
90
80
70
60
50
40
30
20
10
f1 (ppm)

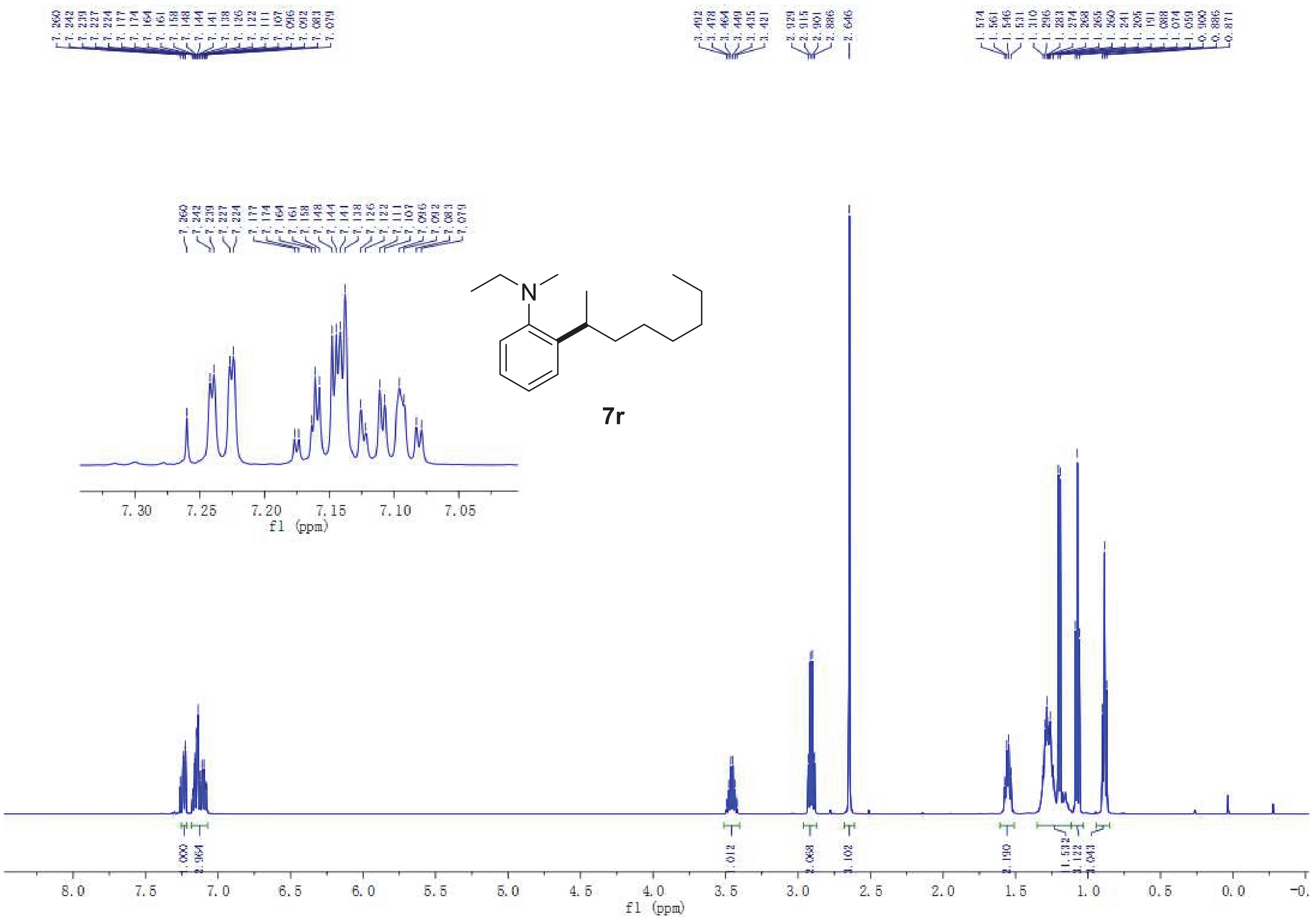
7.260 7.242 7.239 7.227 7.224 7.177 7.174 7.164 7.161 7.158 7.148 7.144 7.141 7.138 7.126 7.122 7.111 7.107 7.096 7.092 7.083 7.079
3.492 3.478 3.464 3.449 3.435 3.421
2.929 2.915 2.901 2.886
2.646
1.574 1.561 1.546 1.531 1.310 1.296 1.283 1.274 1.268 1.265 1.260 1.241 1.205 1.191 1.088 1.074 1.059 0.900 0.886 0.871
N
7r
f1 (ppm)

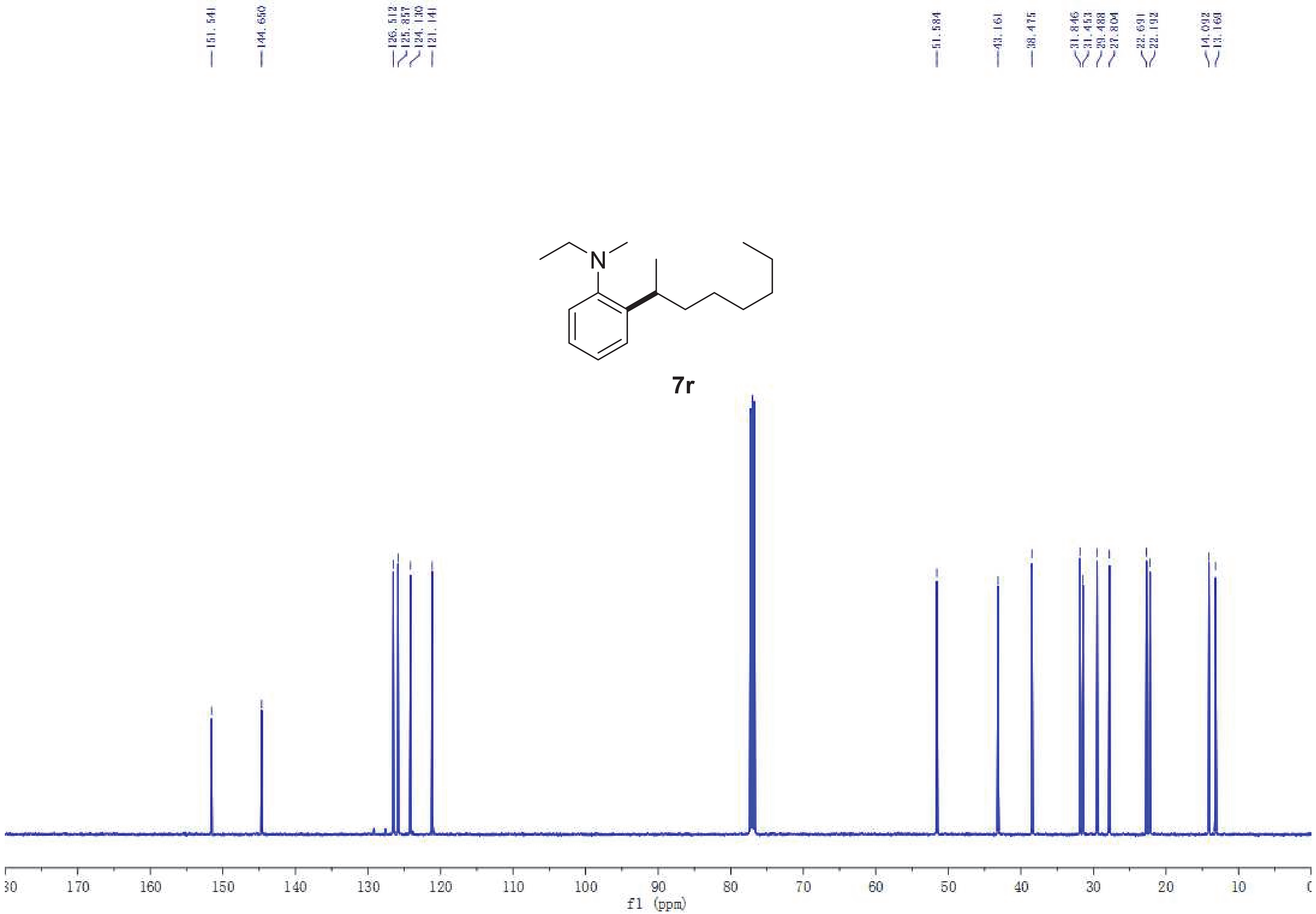

151.541
144.650
126.512
125.857
124.130
121.141
51.584
43.161
38.475
31.846
31.453
29.488
27.804
22.691
22.192
14.092
13.160
N
7r
170
160
150
140
130
120
110
100
90
80
70
60
50
40
30
20
10
f1 (ppm)
